# Cardiovascular mortality attributable to dietary risk factors in 51 countries in the WHO European Region from 1990 to 2016: a systematic analysis of the Global Burden of Disease Study

**DOI:** 10.1007/s10654-018-0473-x

**Published:** 2018-12-14

**Authors:** Toni Meier, Kira Gräfe, Franziska Senn, Patrick Sur, Gabriele I. Stangl, Christine Dawczynski, Winfried März, Marcus E. Kleber, Stefan Lorkowski

**Affiliations:** 1 Institute for Agricultural and Nutritional Sciences, Martin Luther University Halle-Wittenberg, Von-Danckelmannplatz 2, 06120 Halle (Saale), Germany; 2 Competence Cluster for Nutrition and Cardiovascular Health (nutriCARD), Halle-Jena-Leipzig, Germany; 3 Institute of Nutritional Sciences, Friedrich Schiller University Jena, Jena, Germany; 4 Fifth Department of Medicine, Medical Faculty Mannheim, Heidelberg University, Heidelberg, Germany; 5 Clinical Institute of Medical and Chemical Laboratory Diagnostics, Medical University Graz, Graz, Austria; 6 Synlab Academy, Synlab Holding Deutschland GmbH, Mannheim, Germany; 7 Institute of Health Metrics Evaluation (IHME), University of Washington, Seattle, USA

**Keywords:** Nutrition, Public health, Epidemiology, Cardiovascular diseases, European countries, Global Burden of Disease Study

## Abstract

This study was performed to highlight the relationship between single dietary risk factors and cardiovascular diseases (CVDs) in the WHO European Region. We used the comparative risk assessment framework of the Global Burden of Disease Study to estimate CVD mortality attributable to diet; comprising eleven forms of CVDs, twelve food and nutrient groups and 27 risk-outcome pairs in four GBD regions including 51 countries by age and sex between 1990 and 2016. In 2016, dietary risks were associated with 2.1 million cardiovascular deaths (95% uncertainty interval (UI), 1.7–2.5 million) in the WHO European Region, accounting for 22.4% of all deaths and 49.2% of CVD deaths. In terms of single dietary risks, a diet low in whole grains accounted for approximately 429,000 deaths, followed by a diet low in nuts and seeds (341,000 deaths), a diet low in fruits (262,000 deaths), a diet high in sodium (251,000 deaths), and a diet low in omega-3 fatty acids (227,000 deaths). Thus, with an optimized, i.e. balanced diet, roughly one in every five premature deaths could be prevented. Although age-standardized death rates decreased over the last 26 years, the absolute number of diet-related cardiovascular deaths increased between 2010 and 2016 by 25,600 deaths in Western Europe and by 4300 deaths in Central Asia. In 2016, approximately 601,000 deaths (28.6% of all diet-related CVD deaths) occurred among adults younger than 70 years. Compared to other behavioural risk factors, a balanced diet is a potential key lever to avoid premature deaths.

**Electronic supplementary material:**

The online version of this article (10.1007/s10654-018-0473-x) contains supplementary material, which is available to authorized users.

## Introduction

With the number of attributed deaths rising from 12.3 million in the year 1990 to more than 17.6 million in 2016, cardiovascular diseases (CVDs) are the leading cause of death worldwide [1]. In addition to lifestyle factors such as physical inactivity, smoking and the abuse of alcohol, a suboptimal diet constitutes a major risk for developing CVDs [2]. According to the Global Burden of Disease Study (GBD) 2016 [3], more than 9.1 million premature deaths from CVDs worldwide are attributable to dietary risks, which equals 52% of all CVD-related deaths in the year 2016. Therefore, optimized dietary patterns might be an effective lever to overcome the burden of CVDs. Although several studies have elucidated the roles of distinct dietary and metabolic risk factors in CVDs [4–12], a corresponding analysis of countries in the WHO European Region, differentiating age and sex groups and including several key food and nutrient groups, has not yet been reported. The aim of this study is therefore (1) to describe the status of cardiovascular deaths attributable to dietary risks at a national level in 2016; (2) to review corresponding mortality trends of the last 26 years, with a particular focus on the period from 2010 to 2016; and (3) to discuss possible policy options to address the burden of diet-related CVDs.

## Methods

### Data input, scope and modelling

For this analysis, data from the comparative risk assessment (CRA) framework of the GBD was used to quantify CVD deaths attributable to twelve dietary risks, grouped by age and sex, between 1990 and 2016 for 51 countries in four GBD regions (Central Europe: Albania, Bosnia and Herzegovina, Bulgaria, Croatia, Czech Republic, Hungary, Macedonia, Montenegro, Poland, Romania, Serbia, Slovakia, Slovenia; Eastern Europe: Belarus, Estonia, Latvia, Lithuania, Moldova, Russia, Ukraine; Western Europe: Andorra, Austria, Belgium, Cyprus, Denmark, Finland, France, Germany, Greece, Iceland, Ireland, Israel, Italy, Luxembourg, Malta, Netherlands, Norway, Portugal, Spain, Sweden, Switzerland, United Kingdom; Central Asia: Armenia, Azerbaijan, Georgia, Kazakhstan, Tajikistan, Turkmenistan, Turkey, Uzbekistan); see Supplemental Table 1. Monaco and San Marino were not considered in the assessment.

Of the 15 dietary risk factors covered in the GBD CRA framework, twelve are relevant to the development of CVDs, composing 27 diet-disease pairs (Supplementary Appendix and in Appendix Table 4). The twelve dietary factors are as follows: a diet low in fibre, fruits, legumes, nuts and seeds, polyunsaturated fatty acids (PUFA), seafood omega-3 fatty acids, vegetables, and whole grains and a diet high in processed meat, sodium, sugar-sweetened beverages (SSB) and trans fatty acids. The CVDs considered are as follows: aortic aneurysm, atrial fibrillation and flutter, cardiomyopathy and myocarditis, endocarditis, haemorrhagic stroke, hypertensive heart disease, ischaemic heart disease, ischaemic stroke, peripheral vascular disease, rheumatic heart disease, and other cardiovascular and circulatory diseases.

The inputs to this analysis included the exposure level (consumption) of each risk factor, the effect size of the risk factor on each disease endpoint, the risk factor level associated with the lowest risk (TMREL), and the total number of deaths from each disease endpoint. Consumption data were gathered from multiple sources including nutrition surveys, household budget surveys, and United Nations FAO Food Balance Sheets and Supply and Utilization Accounts. Furthermore, for sodium and trans fatty acids, data on 24-hour urinary sodium and availability of partially hydrogenated vegetable oil in packaged foods were used, respectively. All dietary data (other than sodium and SSB) were standardized to 2000 kcal/day. We modelled missing country-year data from FAO using a space–time Gaussian process regression and lag-distributed country income as covariate. For each dietary factor, we estimated the global age pattern of consumption based on nutrition surveys (i.e., 24-hour diet recall) and applied that age pattern to the FAO data.

Whereas the intake levels of fibre, seafood omega-3 PUFA, PUFA, and saturated fatty acids were assessed using data from United Nations FAO Supply and Utilization Accounts, the intake levels of fruits and whole grains were derived from the United Nations FAO Food Balance Sheets. Further details can be found in Table 1 and the Appendix.

**Table 1:** Dietary risk factor, exposure definition and optimal level (theoretical minimum risk exposure level)

No	Dietary risk factor	Exposure definition	Theoretical minimum risk exposure level (TMREL) per person
1	Diet low in fiber	Average daily intake of fibre from all sources including fruits, vegetables, grains, legumes, and pulses	Intake of fiber between 19 and 28 grams per day
2	Diet low in fruits	Average daily intake of fruits (fresh, frozen, cooked, canned, or dried fruits, excluding fruit juices and salted or pickled fruits)	Intake of fruits between 200 and 300 grams per day
3	Diet low in legumes	Average daily intake of legumes (fresh, frozen, cooked, canned, or dried legumes)	Intake of legumes between 50 and 70 grams per day
4	Diet low in nuts and seeds	Average daily intake of nuts and seeds	Intake of nuts and seeds between 16 and 25 grams per day
5	Diet low in polyunsaturated fatty acids	Average daily intake of omega-6 fatty acids from all sources, mainly liquid vegetable oils, including soybean oil, corn oil, and safflower oil	Intake of polyunsaturated fatty acids between 9 and 13% of total daily energy
6	Diet low in seafood omega-3 fatty acids	Average daily intake of eicosapentaenoic acid and docosahexaenoic acid	Intake of seafood omega-3 fatty acids between 200 and 300 milligrams per day
7	Diet low in vegetables	Average daily intake of vegetables (fresh, frozen, cooked, canned, or dried vegetables, excluding legumes and salted or pickled vegetables, juices, nuts, and seeds, and starchy vegetables such as potatoes or corn)	Intake of vegetables between 290 and 430 grams per day
8	Diet low in whole grains	Average daily intake of whole grains (bran, germ, and endosperm in their natural proportion) from breakfast cereals, bread, rice, pasta, biscuits, muffins, tortillas, pancakes, and other sources	Intake of whole grains between 100 and 150 grams per day
9	Diet high in processed meat	Average daily intake of meat preserved by smoking, curing, salting, or addition of chemical preservatives	Intake of processed meat between 0 and 4 grams per day
10	Diet high in sodium	24 h urinary sodium measured in g/day	24 h urinary sodium between 1 and 5 grams per day
11	Diet high in sugar-sweetened beverages	Average daily intake of beverages with ≥ 50 kcal per 226.8 g serving, including carbonated beverages, sodas, energy drinks, fruit drinks, but excluding 100% fruit and vegetable juices	Intake of sugar-sweetened beverages between 0 and 5 grams per day
12	Diet high in trans fatty acids	Average daily intake of trans fat from all sources, mainly partially hydrogenated vegetable oils and ruminant products	Intake of trans fatty acids between 0 and 1% of total daily energy

### Relative risks, theoretical minimum-risk exposure level, mediation

We obtained the relative risk of each disease endpoint per serving of the dietary components from the most recent dose–response meta-analyses of prospective observational studies and, where available, from randomized controlled trials [3, 13]. In GBD 2015 and 2016, the relative risks for the following risk-outcome pairs were updated: diet low in fruits-ischaemic heart disease; diet low in fruits-ischaemic stroke; diet low in fruits-haemorrhagic stroke; diet low in legumes-ischaemic heart disease, diet low in vegetables-ischaemic heart disease; diet low in vegetables-ischaemic stroke; diet low in vegetables-haemorrhagic stroke; diet low in whole grains-ischaemic heart disease; diet low in whole grains-ischaemic stroke; diet low in whole grains-haemorrhagic stroke; and diet low in fibre-ischaemic heart disease. To estimate the range of the theoretical minimum-risk exposure level (TMREL) for each dietary factor, we calculated the level of intake associated with the lowest risk of mortality from each disease endpoint based on the studies included in the meta-analyses of the dietary relative risks. Then, we calculated the TMREL as the weighted average using the global number of deaths from each disease outcome. In Table 1, the dietary risks, the exposure definition and the corresponding TMREL considered in this study are summarized. A more detailed description of the methods and the modelling approach used as well as outcome-pair specific relative risks per incremental increase in consumption are presented in the Supplementary Appendix and in Appendix Table 2. To account for multiple risk factor-outcome correlations and the mediation of CVDs via diet-induced metabolic risks, 69 risk factor-mediator pairs were considered in the analysis (Appendix Table 3). In terms of metabolic mediation factors, we distinguish between high body mass index, high total cholesterol, high fasting plasma glucose and high systolic blood pressure. Moreover, diet low in whole grains, diet low in vegetables and diet low in fruits are considered as mediation factors for diet low in fibre. The epidemiological studies used to evaluate the causal relationship between dietary risk-outcome pairs are summarized in Appendix Table 4. Further and more detailed information about the CRA framework of the GBD can be found in the corresponding GBD capstone papers [3, 13].

### Uncertainty ratios and uncertainty intervals

To gauge the level of uncertainty of the risk- and disease-specific results, we calculated the uncertainty ratio (UR). The uncertainty ratio is a dimensionless unit defined as the 95% uncertainty interval (UI) range (95% UI maximum minus 95% UI minimum) divided by the arithmetic mean of 95% UI maximum and 95% UI minimum. The higher the ratio, the greater the corresponding uncertainty [12]. To incorporate the uncertainty of parameters (exposure, relative risk, TMREL, mortality) as well as modelling uncertainty, we followed a Monte Carlo approach. All calculations were repeated 1000 times using one draw of each parameter at each iteration. Using these 1000 draws, we calculated the mean and the UI for the final estimates.

### Adjusting for multiplicity

As the dietary risks covered in the CRA GBD framework are not completely mutually exclusive, they had to be adjusted for multiplicity when the total disease burden alleviation due to all dietary risk factors was calculated. For all numbers and figures presented in this study, we assumed a combined implementation of all risk factors described above. Further and more detailed information about the multiplicity processing can be found in the corresponding GBD capstone papers [3, 13].

## Results

### Deaths attributable to diet-related CVDs in the year 2016

**Fig. 1:**
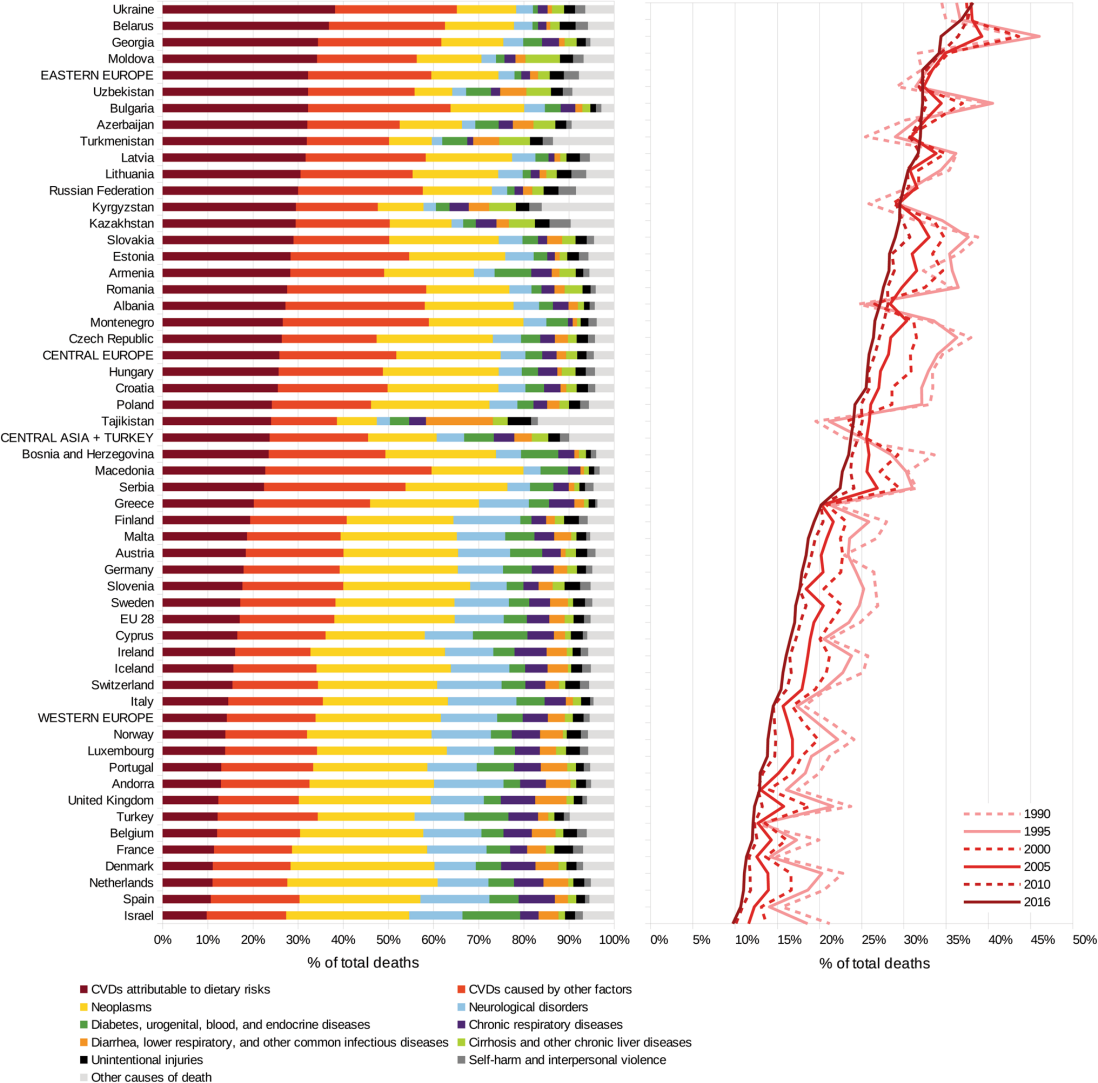
Share of diet-related deaths from CVDs compared to other causes of deaths in the WHO European region in the year 2016 (left) and corresponding trends from 1990 to 2016 (right)

In the year 2016, diet-related risks were associated with 2.1 million deaths (95% UI, 1.7–2.5 million) from CVDs in the WHO European Region, accounting for 22.4% (95% UI, 18.2–27.0%) of total deaths (Fig. 1, Table 2). Concerning specific CVDs, 1.76 million deaths (84% of total diet-related CVD deaths) were due to ischaemic heart disease, followed by ischaemic stroke (175,202 deaths, 8.3%) and haemorrhagic stroke (132,749 deaths, 6.3%). Hypertensive heart disease, rheumatic heart disease, cardiomyopathy and myocarditis, atrial fibrillation and flutter, aortic aneurysm, peripheral vascular disease, endocarditis, other cardiovascular and circulatory diseases were of less importance. The highest uncertainty ratios (UR)—and, therefore, the lowest validity for the results—were observed for endocarditis (UR 1.73) and hypertensive heart disease (UR 1.72; Table 2a). In terms of food and nutrient groups, five risk factors had an attributable fraction greater than 10% of the total diet-related CVD burden: diet low in whole grains (20.4%), diet low in nuts and seeds (16.2%), diet low in fruits (12.5%), diet high in sodium (12.0%), and diet low in seafood omega-3 PUFA (10.8%). The highest uncertainty ratio—and, therefore, the lowest validity for the dietary risks considered in terms of their impacts—was observed in the case of processed meat (0.63), followed by trans fatty acids (0.61) and sodium (0.59); Table 2b.

**Table 2:** Diet-related deaths from CVDs in the WHO European Region in 2016 due to (a) disease group and (b) food or nutrient group

	Number of deaths	95% Uncertainty interval		Uncertainty ratio^a^
(a) **Due to disease group**				
Ischemic heart disease	1,757,053	1,448,146	2,085,227	0.36
Ischemic stroke	175,202	141,174	216,754	0.42
Hemorrhagic stroke	132,749	112,348	160,875	0.36
Hypertensive heart disease	18,886	3214	42,018	1.72
Cardiomyopathy and myocarditis	3394	897	6002	1.48
Atrial fibrillation and flutter	2553	445	4699	1.65
Aortic aneurysm	1840	367	3165	1.58
Peripheral vascular disease	840	131	1562	1.69
Rheumatic heart disease	799	144	1559	1.66
Endocarditis	662	91	1280	1.73
Other cardiovascular and circulatory diseases	5659	1234	9719	1.55
Sum	2099,637	1,708,193	2,532,859	0.39
(b) **Due to food or nutrient group**				
Diet low in whole grains	429,220	374,656	492,324	0.27
Diet low in nuts and seeds	341,185	297,925	391,234	0.27
Diet low in fruits	261,965	211,927	317,216	0.40
Diet high in sodium	251,437	179,195	328,682	0.59
Diet low in seafood omega-3 PUFA	227,276	182,913	276,161	0.41
Diet low in vegetables	188,915	146,685	234,904	0.46
Diet low in legumes	148,668	121,269	179,024	0.38
Diet low in fiber	120,241	101,607	141,269	0.33
Diet low in PUFA	78,101	62,833	94,923	0.41
Diet high in processed meat	34,113	23,666	45,238	0.63
Diet high in trans fatty acids	16,182	11,369	21,318	0.61
Diet high in sugar-sweetened beverages	2334	1997	2717	0.31
Sum	2099,637	1,708,193	2,532,859	0.39

^a^The uncertainty ratio is a dimensionless unit, defined as the 95% UI range (95% UI maximum minus 95% UI minimum) divided by the arithmetic mean of 95% UI maximum and 95% UI minimum [12]. The higher the ratio the higher is the corresponding uncertainty

Within the WHO European Region, the fraction of deaths attributable to diet-related CVDs varies considerably between the regions: Whereas Eastern Europe and Central Asia faced the greatest burden in terms of age-standardized deaths rates (304 and 298 per 100,000, respectively), Eastern Europe showed the highest burden (937,000) and Central Asia the lowest burden (227,000) in terms of absolute deaths. The smallest burden in terms of percentage of deaths and deaths per 100,000 people was observed in Western Europe (14.2%, 62 deaths per 100,000). Within Eastern Europe, the highest number of absolute deaths due to diet-related CVDs was observed for Russia (599,000 deaths), followed by Ukraine (253,000 deaths). Further, Ukraine showed the highest corresponding fraction (38.2% of total deaths) among all countries considered in this study. In terms of age-standardized deaths per 100,000 inhabitants, the highest rates in Eastern Europe were identified for Ukraine (349 deaths per 100,000), Moldova (328 deaths per 100,000) and Belarus (313 deaths per 100,000). Within Central Europe, the greatest burden in terms of total deaths was observed in Poland (94,000), followed by Romania (70,000), and Bulgaria (35,000). In relative terms, the highest percentage was identified in Bulgaria (32.2%), followed by Slovakia (29.0%) and Romania (27.6%). In terms of age-standardized death rates, the ranking was almost the same: first, Bulgaria (260 deaths per 100,000); second, Slovakia and Romania, each having 206 deaths per 100,000.

Within Western Europe, the highest absolute numbers of CVD deaths related to dietary risks in the year 2016 were observed in Germany (165,000), followed by Italy (97,000) and the UK (75,000). In relative terms, the highest percentage was identified in Greece (20.2%), followed by Finland (19.4%), Malta (18.7%) and Austria (18.4%). In terms of age-standardized death rates, the ranking was almost the same: Greece (100 deaths per 100,000), followed by Cyprus (88 per 100,000) and Finland, Malta and Germany (each having 87 per 100,000).

In Central Asia, the greatest burdens in terms of total deaths were observed in Uzbekistan (66,000), Turkey (44,000) and Kazakhstan (40,000). In relative terms, the highest percentages were identified in Georgia (34.4%), Uzbekistan (32.2%) and Azerbaijan (32.0%). In terms of age-standardized deaths, the highest rate was observed in Uzbekistan (394 deaths per 100,000), followed by Turkmenistan (367 per 100,000) and Kyrgyzstan (350 per 100,000); Table 3.

**Table 3:** Number of deaths, deaths in % and deaths per 100,000 people (age-standardized) by CVD attributable to dietary risk factors (incl. 95% uncertainty interval)

	Number of deaths	Deaths in %	Deaths per 100,000 people (age-standardized)
**Western Europe**	**594,295**	**14.2**	**64**
	(496,931–700,631)	(11.9–16.7)	(54–75)
Andorra	94	12.9	54
	(71–121)	(9.7–16.6)	(40–70)
Austria	15,186	18.4	83
	(12,158–18,203)	(14.7–22.1)	(68–99)
Belgium	13,608	12.0	59
	(11,059–16,384)	(9.8–14.5)	(48–70)
Cyprus	1195	16.5	88
	(984–1419)	(13.6–19.6)	(73–104)
Denmark	5854	11.1	55
	(4716–7190)	(8.9–13.6)	(44–67)
Finland	10,029	19.4	87
	(8335–11,766)	(16.1–22.7)	(72–102)
France	66,801	11.4	46
	(55,087–79,825)	(9.4–13.6)	(38–54)
Germany	164,639	17.9	87
	(136,783–194,795)	(14.9–21.2)	(73–103)
Greece	25,785	20.2	100
	(20,764–31,139)	(16.3–24.4)	(81–119)
Iceland	353	15.7	69
	(297–413)	(13.2–18.4)	(58–81)
Ireland	4984	16.0	79
	(4033–5947)	(13.0–19.2)	(64–95)
Israel	4486	9.8	43
	(3279–5815)	(7.1–12.6)	(32–57)
Italy	96,977	14.5	61
	(76,694–118,592)	(11.5–17.8)	(49–74)
Luxembourg	568	13.8	62
	(450–694)	(10.9–16.9)	(49–76)
Malta	644	18.7	87
	(506–805)	(14.7–23.3)	(68–108)
Netherlands	16,301	11.0	52
	(13,435–19,917)	(9.1–13.5)	(43–63)
Norway	5818	13.9	61
	(4657–7055)	(11.1–16.9)	(49–74)
Portugal	14,499	13.0	63
	(11,574–17,620)	(10.3–15.8)	(51–76)
Spain	44,617	10.7	43
	(35,661–54,825)	(8.5–13.1)	(35–53)
Sweden	16,164	17.2	77
	(12,849–19,567)	(13.7–20.8)	(61–93)
Switzerland	10,349	15.4	60
	(7580–13,259)	(11.3–19.8)	(44–76)
United Kingdom	75,343	12.3	62
	(66,069–85,339)	(10.8–13.9)	(55–70)
**Central Europe**	**341,032**	**25.9**	**177**
	(288,961–393,849)	(21.9–29.9)	(144–214)
Albania	6085	27.2	174
	(4813–7475)	(21.5–33.4)	(137–212)
Bosnia and Herzegovina	9081	23.4	153
	(7224–11,195)	(18.6–28.9)	(121–188)
Bulgaria	35,298	32.2	260
	(28,440-43,692)	(25.9–39.8)	(211–319)
Croatia	14,208	25.5	170
	(11,607–17,005)	(20.8–30.5)	(140–203)
Czech Republic	28,574	26.4	159
	(24,428–32,717)	(22.6–30.2)	(136–181)
Hungary	33,539	25.7	192
	(27,930–39,812)	(21.4–30.5)	(160–228)
Macedonia	4948	22.7	197
	(3852–6211)	(17.7–28.5)	(152–249)
Montenegro	1582	26.6	186
	(1276–1922)	(21.4–32.3)	(150–226)
Poland	94,291	24.2	150
	(78,937–110,004)	(20.3–28.2)	(125–175)
Romania	70,166	27.6	206
	(56,558–85,548)	(22.2–33.6)	(167–250)
Serbia	23,971	22.4	168
	(18,949–29,963)	(17.7–28.1)	(134–209)
Slovakia	15,643	29.0	206
	(13,002–18,262)	(24.1–33.8)	(171–240)
Slovenia	3646	17.7	86
	(2889–4515)	(14.0–21.9)	(69–106)
**Eastern Europe**	**936,950**	**32.2**	**304**
	(738,494–1162,390)	(25.4–40.0)	(240–376)
Belarus	44,568	36.8	313
	(36,132–53,629)	(29.9–44.3)	(254–374)
Estonia	4491	28.3	170
	(3484–5538)	(21.9–34.9)	(133–209)
Latvia	9004	31.7	232
	(7428–10,536)	(26.1–37.1)	(192–270)
Lithuania	12,187	30.5	214
	(10,339–14,157)	(25.9–35.4)	(182–247)
Moldova	14,746	34.2	328
	(12,420–17,069)	(28.8–39.6)	(276–381)
Russia	598,759	30.0	291
	(432,818–794,563)	(21.7–39.8)	(212–385)
Ukraine	253,196	38.2	349
	(196,460–328,161)	(29.6–49.5)	(271–450)
**Central Asia + Turkey**	**227,360**	**23.7**	**289**
	(183,808–275,990)	(19.2–28.8)	(240–343)
Armenia	7686	28.2	204
	(6392–8963)	(23.5–32.9)	(171–238)
Azerbaijan	22,418	32.0	319
	(17,743–27,380)	(25.4–39.1)	(254–391)
Georgia	16,486	34.4	278
	(13,290–19,833)	(27.7–41.4)	(227–333)
Kazakhstan	39,632	29.5	306
	(31,982–19,833)	(23.8–35.9)	(248–371)
Kyrgyzstan	10,627	29.5	350
	(9137–12,225)	(25.4–34.0)	(299–402)
Tajikistan	9932	24.0	310
	(8369–11,930)	(20.2–28.9)	(260–371)
Turkey	44,298	12.1	67
	(33,452–57,146)	(9.2–15.7)	(50–87)
Turkmenistan	10,483	31.9	376
	(9186–11,876)	(28.0–36.2)	(326–427)
Uzbekistan	65,799	32.2	394
	(54,256–78,339)	(26.6–38.3)	(323–472)
**EU-28**	**894,241**	**17.1**	**85**
	(736,198–1065,840)	(14.4–19.8)	(72–98)
**Sum/ weighted average**	**2099,637**	**22.4**	**208**
	(1,708,193–2,532,859)	(18.2–27.0)	(171–249)

### Mortality from diet-related CVDs from 1990 to 2016

In the 51 countries considered, the share of diet-related CVD deaths out of total mortality ranged between 38.2% in the Ukraine and 9.8% in Israel. In 10 out of these 51 countries, an increase in this fraction was observed from 1990 to 2016. The increase was largest in Turkmenistan (+ 26%), followed by Tajikistan (+ 23%) and Kyrgyzstan (+ 14%), Fig. 1. From 2010 to 2016, the largest increases in the attributable fraction were observed for Turkmenistan (+ 3.9%), Kyrgyzstan (+ 1.5%) and Andorra (+ 1.4%).

The largest reduction of the attributable fraction from 1990 to 2016 was identified for Israel (− 54%), followed by Denmark (− 51%) and the UK (− 48%). On average, the observed reduction was − 34% for Western Europe and − 25% for Central Europe. In Central Asia and Eastern Europe, the corresponding fractions increased by + 3.0% and + 0.4%, respectively. From the 2010 to 2016, the largest decreases in the attributable fraction were observed for the United Kingdom (− 7.1%), the Netherlands (− 6.9%) and Serbia (− 6.7%).

In terms of absolute deaths, the mortality from CVDs attributable to dietary risks decreased in the WHO Europe Region by 187,000 deaths per year from 2.3 million in 1990 to 2.1 million in 2016 (drop by 8.2%), whereas the regional subdivisions considered in this study developed unevenly: While the number of deaths per year increased by 124,000 (from 813,000 in 1990 to 937,000 in 2016) in Eastern Europe and by 29,000 (from 198,000 in 1990 to 227,000 in 2016) Central Asia, the number decreased by 110,000 (from 451,000 in 1990 to 341,000 in 2016) in Central Europe and dropped by 230,000 (from 824,000 in 1990 to 594,000 in 2016) in Western Europe. During this period, the largest increase in death numbers occurred in Russia, where 91,000 additional deaths occurred in 2016 compared to 1990 (1990: 508,000 deaths; 2016: 599,000 deaths).

**Fig. 2:**
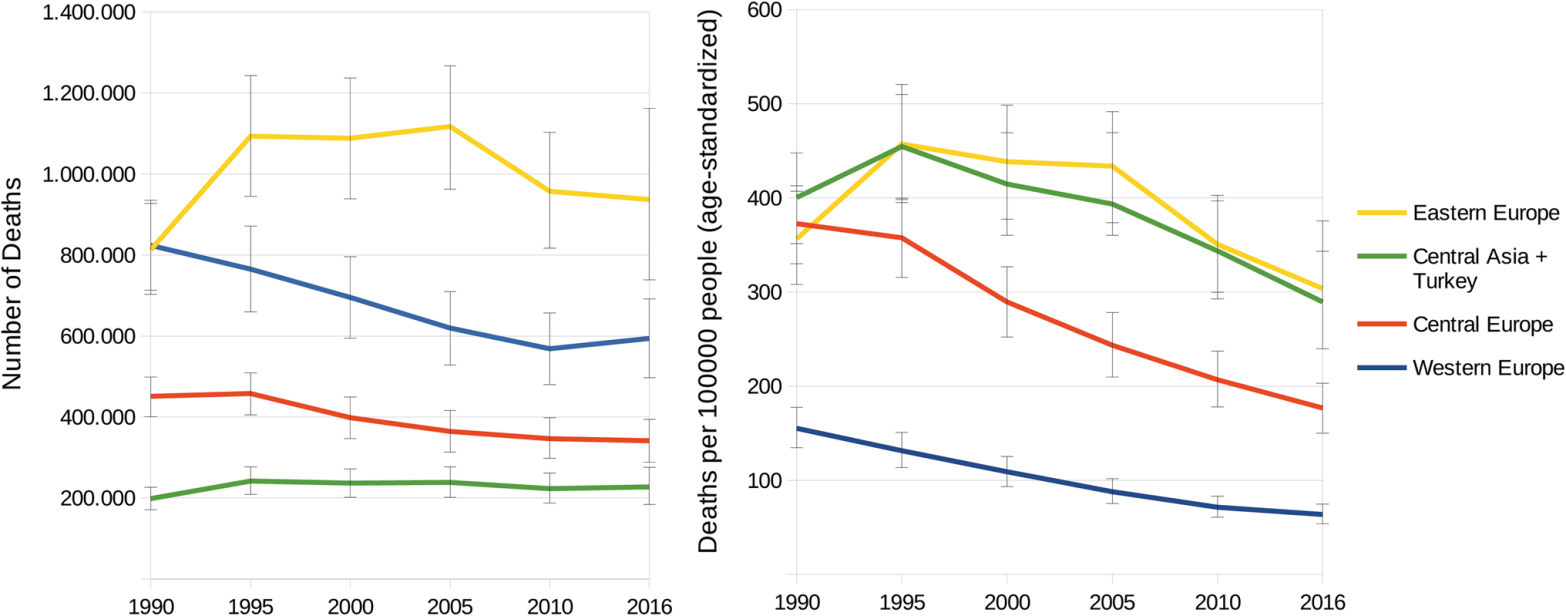
Number of deaths (left) and death rate per 100,000 people (age-standardized) (right) in Eastern, Central and Western Europe and Central Asia related to CVDs attribute to dietary risks from 1990 to 2016 (incl. 95% uncertainty interval)

However, although from 2010 to 2016 a decline in the absolute number of deaths was observed for Central Europe (− 5400 deaths per year) and Eastern Europe (− 20,300 per year), Western Europe faced an increase of 25,600 deaths from CVDs attributable to dietary risks, from 569,000 in 2010 to 594,000 deaths in 2016 (+ 4.5%), in the same period. In addition, in Central Asia, a slight increase of 4300 deaths per year was observed from 2010 to 2016 (+ 1.9%). In total, 29 countries out of 51 showed increased absolute numbers of diet-related CVD deaths from 2010 to 2016 (Fig. 2, Supplementary Appendix and in Appendix Table 5).

In terms of age-standardized mortality, the CVD death rate attributable to dietary risks has fallen in the WHO European Region over the last 26 years (except for an increase in Eastern Europe and Central Asia from 1990 to 1995). However, the pace of the reduction slowed down in the period from 2010 to 2016. Whereas in Western Europe the rate in 2016 (64 deaths per 100,000) was almost the same as in 2010 (71 deaths per 100,000), the magnitude of change in the mortality rate is more attenuated in Central Asia (2010: 344 per 100,000; 2016: 289 per 100,000), Eastern Europe (2010: 351 per 100,000; 2016: 304 per 100,000), and Central Europe (2010: 207 per 100,000; 2016: 177 per 100,000; Fig. 2).

**Fig. 3:**
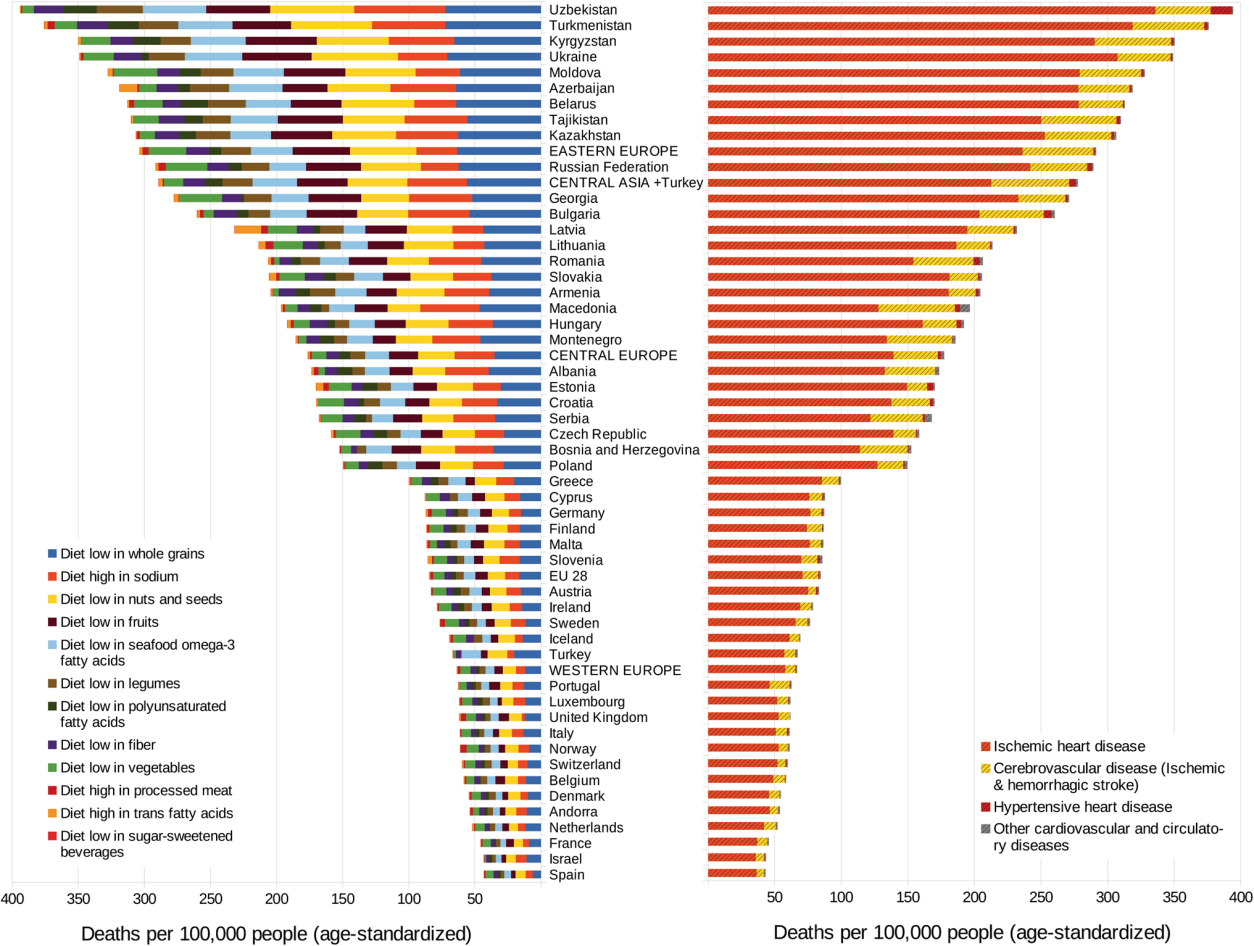
Diet-related deaths per 100,000 people (age-standardized) from CVDs due to risk factors (left) and disease groups (right) in 2016

The age-standardized diet-related CVD mortality in 2016 varied widely from a rate of 43 per 100,000 in Israel and Spain to 394 per 100,000 in Uzbekistan. Eastern European and Central Asian countries (exception: Turkey) consistently had the highest mortality rates (weighted average: 304 and 289 per 100,000, respectively). With the exception of Turkey (67 per 100,000), diet-related CVD rates in all countries in these two regions ranged between 170 per 100,000 in Estonia and 394 per 100,000 in Uzbekistan. Central Europe (weighted average: 177 per 100,000) ranged between 86 deaths per 100,000 in Slovenia and 260 per 100,000 in Bulgaria. In Western Europe (weighted average: 64 per 100,000), the highest rate was found in Greece (100 deaths per 100,000) and the lowest in Spain and Israel (each with 43 deaths per 100,000). In the EU-28, an average rate of 85 deaths per 100,000 was observed in 2016. On the disease level, countries with higher mortality due to ‘a diet high in sodium’ showed a higher death rate due to cerebrovascular diseases and hypertensive heart disease, mainly Central European countries such as Bulgaria, Macedonia, Romania, and Serbia, but also on a lower level in countries such as Portugal and Turkey (Fig. 3).

### Differences between gender and age groups

**Fig. 4:**
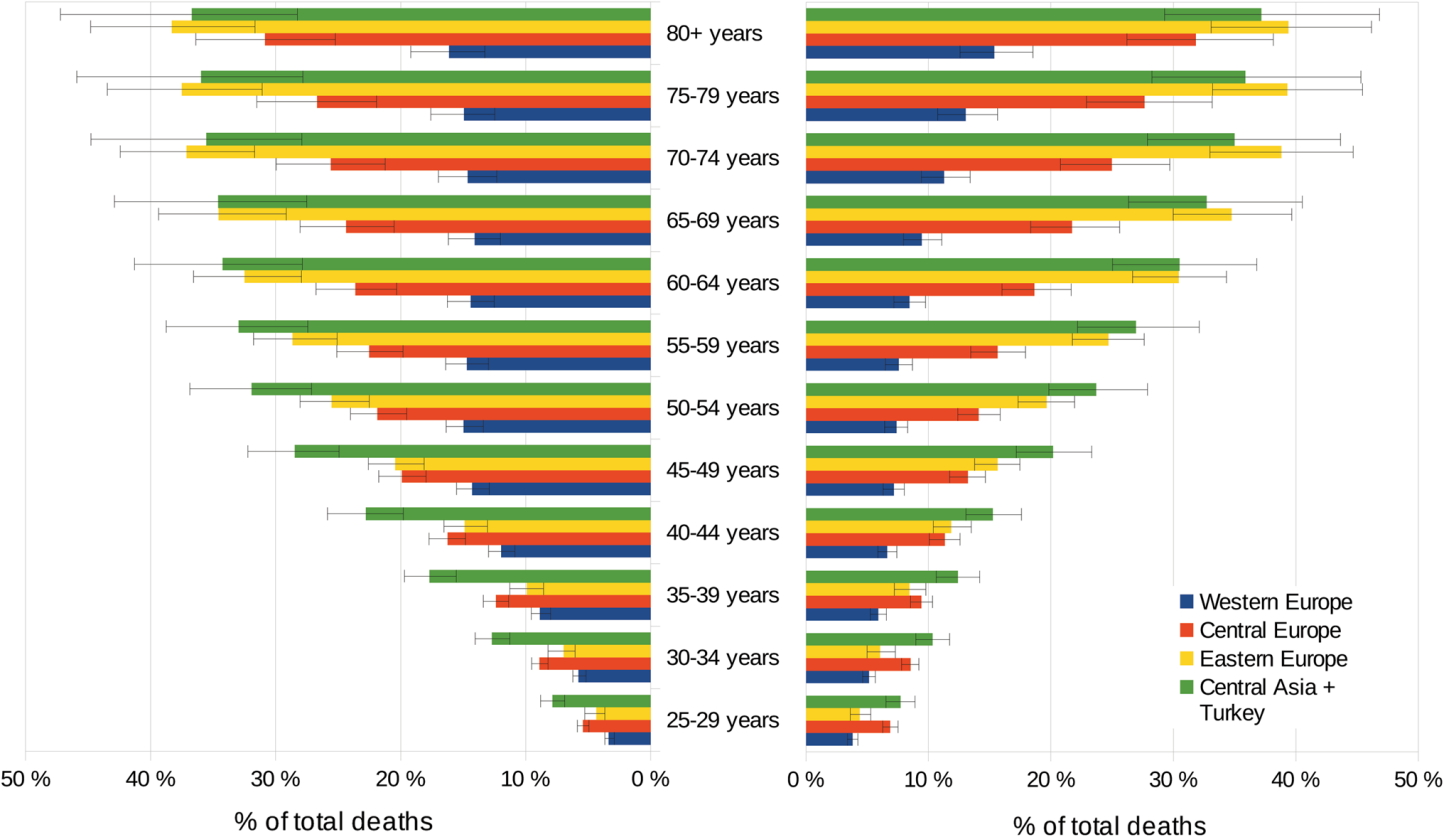
Attributable fraction of diet-related deaths from CVDs due to sex and age-group in 2016, male (left), female (right) (incl. 95% uncertainty interval)

Diet-related CVDs increased steadily as a proportion of total deaths across older age groups, with the greatest increases in Central Asia, Eastern and Central Europe and among men in general. Whereas among older men in Western Europe the attributable fraction plateaus at approximately 15% at the age of > 50 years, in Central Asia and Central and Eastern Europe the attributable burden increases more sharply at a younger age and plateaus at approximately 35% in Central Asia and Eastern Europe and 25% in Central Europe at the age of > 55 years. In the female population, the attributable fraction is less pronounced at a younger age but exceeds the corresponding proportion of men in Eastern Europe at an age of > 65 years, in Central Europe at an age of > 75 years and in Central Asia at an age of > 80 years, peaking at 39% in Eastern Europe, 37% in Central Asia and 32% in Central Europe in the age group 80 + years (Fig. 4).

Nearly 601,000 deaths (28.6% of all diet-related CVD deaths) occurred in the WHO European Region among adults younger than 70 years, whereas 420,000 deaths occurred in men and 181,000 in women. The highest share of diet-related CVD deaths in adults younger than 70 years were observed with 42.5% in Central Asia equalling 97,000 premature deaths, followed by Eastern Europe (33.7%, 316,000 deaths), Central Europe (26.0%, 87,000 deaths) and Western Europe (16.9%, 100,000 deaths). In the EU-28 approximately 178,000 deaths (19.9% of all diet-related CVD deaths) occurred among adults < 70 years, of which 132,000 deaths in men and 46,000 were in women.

Between 2010 and 2016, an increase in absolute diet-related CVD deaths was observed in 32 (out of 51) countries, leading to 20,000 additional deaths in 2016 among adults younger than 70 years. Most of these deaths were observed in Germany (+ 2700), followed by Belarus (+ 2600), Kazakhstan (+ 1800), Romania (+ 1700) and the Ukraine (+ 1600). Details at the country level can be found in the Supporting Material.

### The impact of different food groups on CVD outcomes

**Fig. 5:**
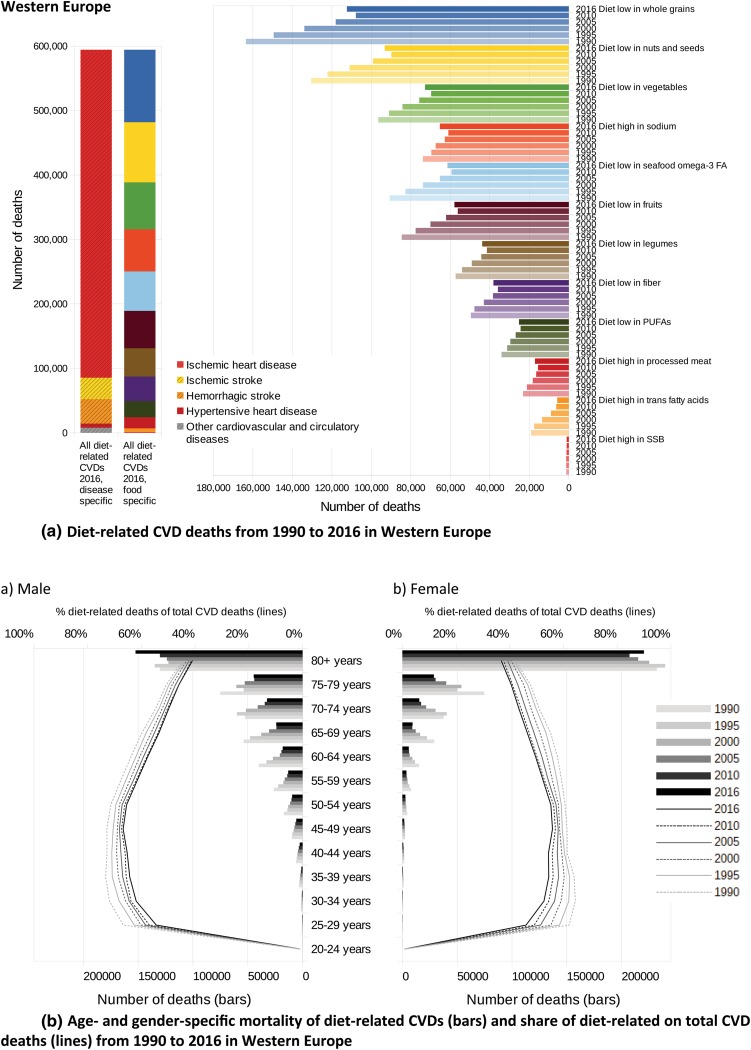

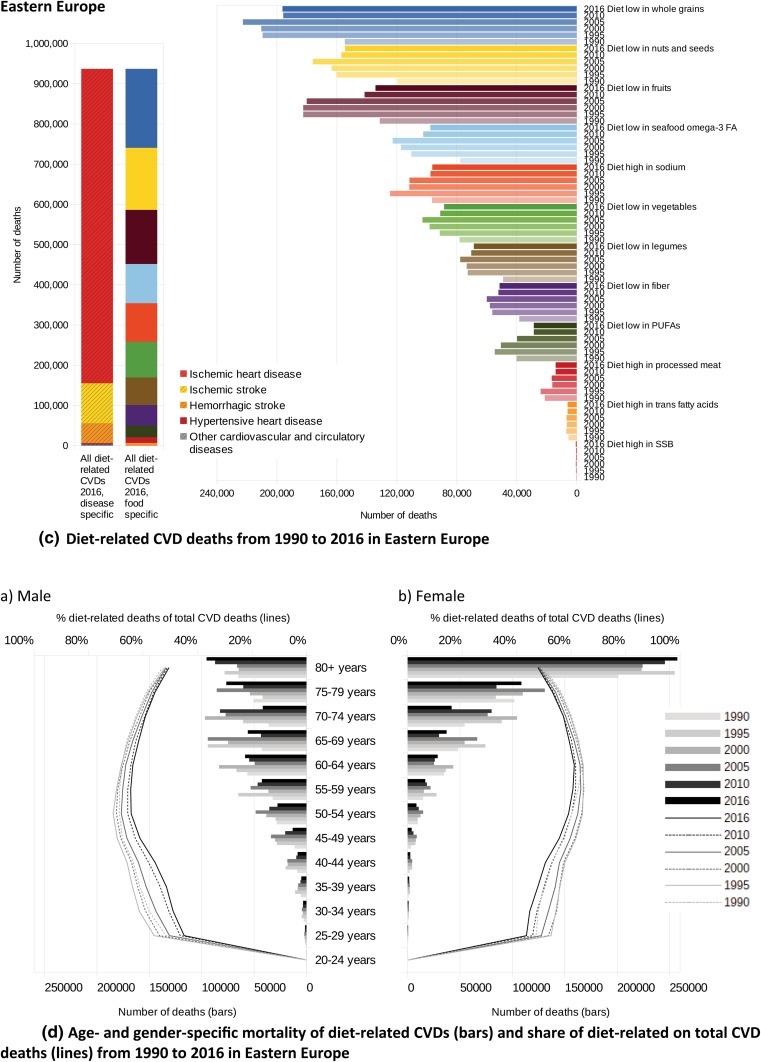

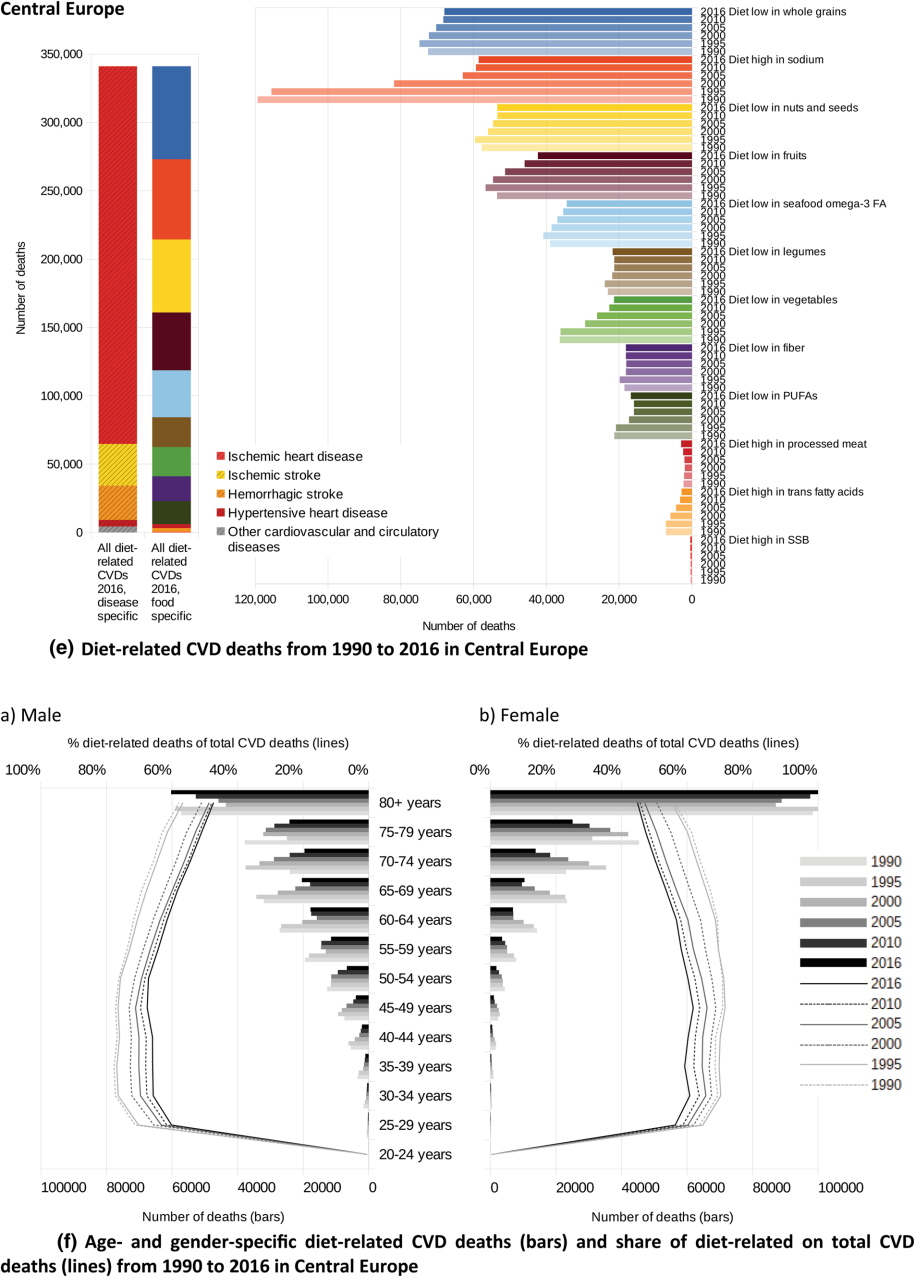

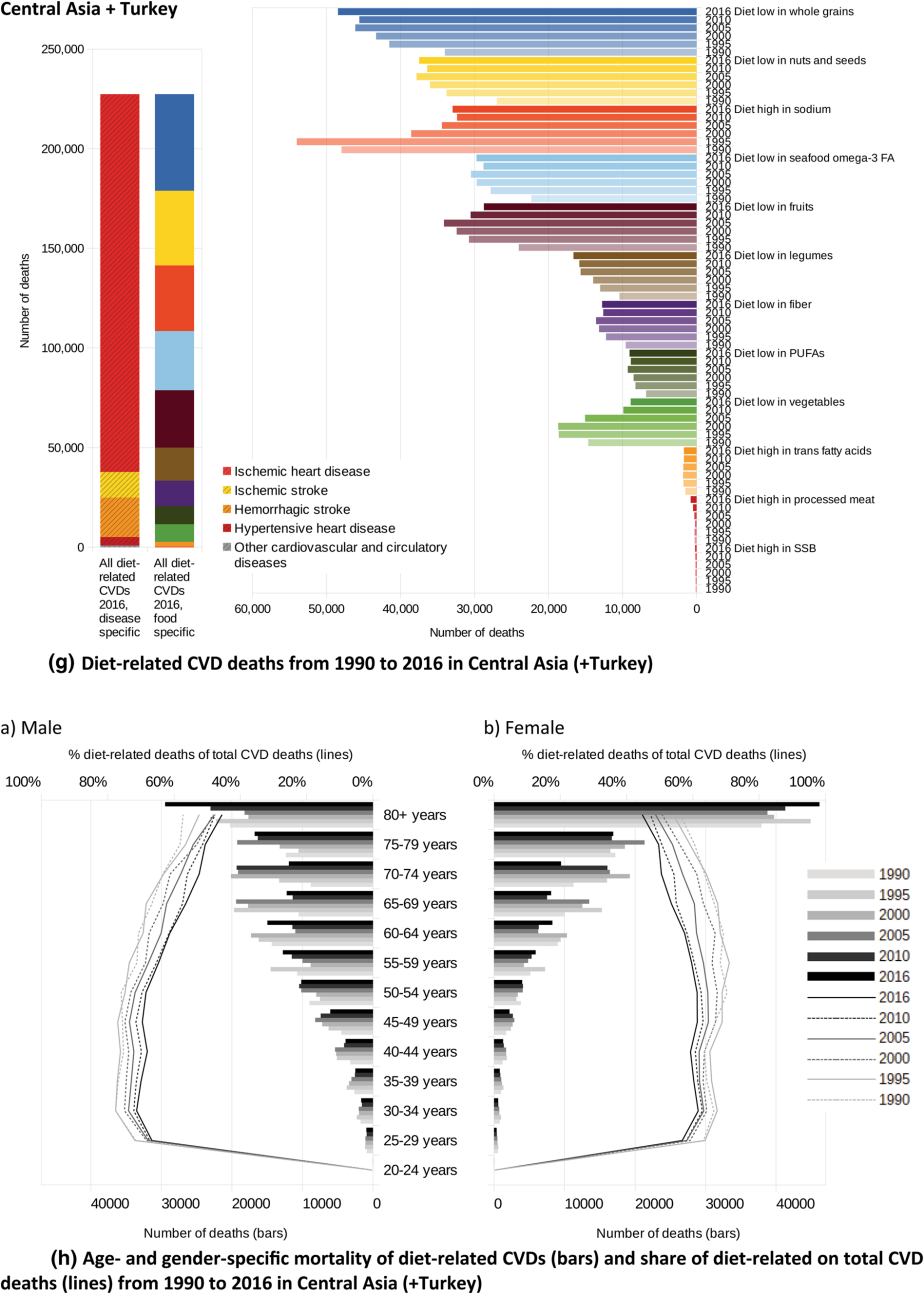
**a** Diet-related CVD deaths from 1990 to 2016 in Western Europe, **b** Age- and gender-specific mortality of diet-related CVDs (bars) and share of diet-related on total CVD deaths (lines) from 1990 to 2016 in Western Europe, **c** Diet-related CVD deaths from 1990 to 2016 in Eastern Europe, **d** Age- and gender-specific mortality of diet-related CVDs (bars) and share of diet-related on total CVD deaths (lines) from 1990 to 2016 in Eastern Europe, **e** Diet-related CVD deaths from 1990 to 2016 in Central Europe, **f** Age- and gender-specific mortality of diet-related CVDs (bars) and share of diet-related on total CVD deaths (lines) from 1990 to 2016 in Central Europe, **g** Diet-related CVD deaths from 1990 to 2016 in Central Asia (+ Turkey), **h** Age- and gender-specific mortality of diet-related CVDs (bars) and share of diet-related on total CVD deaths (lines) from 1990 to 2016 in Central Asia (+ Turkey)

In Fig. 5, the absolute numbers of CVD deaths related to single and aggregated dietary risks are presented from 1990 to 2016. In both cases, multiplicity-adjusted presentation was used, not considering that single risk reductions, separately applied, might lead to even greater improvements in CVD health.

Except for the observation that a diet low in whole grains was the leading risk factor in all GBD regions considered, the ranking of the other dietary risk factors was different across the regions. Whereas in Western Europe, Eastern Europe and in Central Asia the low consumption of nuts and seeds was the second leading risk factor for CVDs, in Central Europe the excessive consumption of sodium caused the second highest mortality—with the consequence of slightly increased mortality due to cerebrovascular diseases (ischaemic and haemorrhagic stroke) and hypertensive heart disease. In Eastern European countries, the excessive consumption of sodium ranked in fifth place, behind the risk factors ‘diet low in nuts and seeds’ and ‘diet low in fruits’ and ‘diet low in omega-3 fatty acids’. Whereas in Central Asia the risk factor ‘diet high in sodium’ ranked third, in Western Europe it ranks fourth. In all four regions, the risks ‘diet high in SSB’ and ‘diet high in trans fatty acids’ were of minor importance.

Although the changes over time followed a consistent pattern, the magnitude of the changes reflected underlying alterations in dietary patterns. In the last two decades, while improvements in diet-related vascular health in Eastern Europe were derived mainly from increased consumption of fruits, PUFAs and omega-3 fatty acids, the reduced consumption of sodium was the largest impacting factor in Central Europe and Central Asia. Of further relevance in these two regions was the increased intake of vegetables and fruits.

In Western Europe since 1990, increased intakes of whole grains, nuts and seeds, vegetables, omega-3 fatty acids and fruits were the leading factors resulting in diminished deaths from CVDs. However, with the exception of ‘diet high in trans fatty acids’ between 2010 and 2016, all risk factors showed an increased impact on CVD deaths, which could be explained by population ageing resulting in an additional 25,600 deaths per year (95% UI: 17,000–36,000). Details on the country level can be found in the Supporting Material.

## Discussion

Using data of the GBD Study 2016 [3], we examined the impact of dietary risks on CVD mortality in the WHO European Region in the period from 1990 to 2016. Although the age-standardized death rates decreased in all considered subregions between 2010 and 2016, the absolute number of diet-related premature cardiovascular deaths increased in 29 (out of 51) countries, with increases in Western Europe by 25,600 and in Central Asia by 4300 deaths (Fig. 2, Supplementary Appendix and in Appendix Table 5). Moreover, in 32 (out of 51) countries, increases in absolute diet-related CVD mortality were observed in adults younger than 70 years, leading to an additional 20,000 deaths in 2016 compared to 2010.

With 22.4% of overall mortality and 49.2% of CVD mortality, dietary factors contribute substantially to the disease burden in the WHO European Region. Compared to other behavioural (and thus modifiable) risk factors (physical inactivity, drug and alcohol abuse, tobacco smoking, etc.), an altered diet is the most effective means of preventing premature deaths from CVDs in the WHO European Region [3]. Nonetheless, questions remain about the most efficient way to promote dietary changes among healthy adults [14]. The impact of a range of dietary interventions and instruments at the population level has been systematically evaluated, ranging from regulatory and market-based instruments to nudging and information-based instruments [15–18]. Although the effectiveness, the acceptance and, ultimately, the durability of each policy measure is case-specific and may vary across countries, the prioritization of public health interventions should be evidence-based and data-driven.

Our analysis has several strengths. To the best of our knowledge, it builds upon the most comprehensive, most current, most consistent and most robust data framework quantifying the risks of disease burden globally [3]. In comparison to similar studies focusing on European countries [19, 20], we present here for the first time the numbers of deaths attributable to eleven different types of CVD and twelve dietary risk factors, accounting for cause-specific mortality by country, age, sex, and time period. Further, we used the most updated effect sizes of diet-disease relationships considering age- and outcome-specific aetiological effect sizes per unit of exposure [3].

Concerning the development of CVD mortality until the year 2010 in Russia, our results confirm the findings of Grigoriev et al. [21] and Shkolnikov et al. [22], who described an unprecedented decline in cardiovascular mortality rates in the period 2000–2010 (particularly for cerebrovascular diseases, less pronounced for heart disease) as the ‘cardiovascular revolution’. The authors state that both behavioural factors (less alcohol consumption, increased consumption of fruits and vegetables) and medical care (increased cardiac surgeries and healthcare expenditures) contributed to this positive trend. However, a stringent decomposition of various factors affecting cardiovascular health was not carried out in these studies. Further, Grigoriev et al. [21] reported that, in 2013, federal healthcare expenditures were reduced, leading to substantial gaps in the healthcare budgets of regional authorities. As 50 out of 81 Russian regions are subsidized by the federal budget, less affluent regions have likely faced underfunding of federally supported medical services. In terms of risk factor exposure, we can confirm the results of Burggraf et al. [23, 24], who analysed the food and nutrient consumption in Russia based on the Russia Longitudinal Monitoring Survey (RLMS-HSE) from 1996 to 2008. Whereas the consumption of fruit increased steadily during this period (from 26.6 to 39.0 kg in rural areas, from 44.3 to 50.8 kg in urban areas per person per year), we observed a strong decline in fruit-related CVDs from 2005 to 2010, moving the risk factor ‘diet low in fruits’ from the second position in 2000 to the third position in 2016 (Appendix Figure 79).

Zatonski et al. [25] examined trends of mortality due to coronary heart disease (CHD) and fat consumption in eleven Eastern and Central European countries from 1990 until 2002. They observed that, in countries where sunflower oil remained the primary oil (such as Russia, Ukraine, Romania, and Bulgaria), the rate of CHD remained stable from 1990 onward. Meanwhile, in countries such as Poland, Czech Republic, Estonia, Latvia and Lithuania, which started to use rapeseed oil, a strong decline in CHD mortality was observed. This finding is confirmed in our study. Although both oils are rich in PUFAs, rapeseed oil contains more α-linoleic acid (ALA, C18:3), an omega-3 fatty acid with atheroprotective properties. Moreover, ALA is partly converted to eicosapentaenoic acid (EPA) and docosahexaenoic acid (DHA), which may protect against CHD and cerebrovascular diseases [26, 27].

### Limitations

The following limitations of our study have to be mentioned. The dietary data were from multiple sources including nationally representative individual-level nutrition surveys, household budget surveys, FAO Food Balance Sheets and Supply and Utilization Accounts, varying in type and magnitude of uncertainty. To overcome this problem, we incorporated these sources of uncertainty into our analysis if possible and quantified the overall UI for the mortality estimates. We used effect sizes from meta-analyses of observational studies that had been adjusted for major confounders (e.g., age, sex, smoking, physical activity); however, the possibility of residual confounding cannot be excluded. In particular, despite adjustment for multiplicity, interrelations between dietary factors may affect our estimated aetiological effect sizes for individual dietary factors.

Moreover, we have to bear in mind that most of the studies which have been included in the meta-analyses are representative for northern American and European populations. Hence, the transferability of the results to Asian populations is limited. Further, in terms of the risk factor with the strongest impact on preventing CVDs (´diet low in whole grains´), we have to note that in the underlying studies the consumption is measured differently and that a common definition of whole grain products does not exist. This could have contributed to the heterogeneity between studies. In addition, given the broad spectrum of whole grain products available it is difficult to assess intake accurately in epidemiological studies. Hence, some degree of measurement error is inevitable [28]. The same statement also applies to the other food and/or nutrient groups considered. As in real life a large number of differently composed foods are consumed, an exact division into distinct food or nutrient groups is impossible. As a consequence resulting disease burdens might be under- or overestimated. Any further epidemiological studies should therefore document the amount and the type of product consumed more accurately. In case of whole grain products a further differentiation between whole grains products based on wheat, rye, oat and barley would be desirable, as different metabolic pathways exist due to the different kinds of containing fibres [29].

Although the meta-analyses used in the GBD CRA framework have been adjusted for several confounders (smoking, alcohol, physical activity, BMI) further confounders remain (deprivation, socioeconomic status, health care access) and are a potential source of bias. However, in terms of the risk factors diet low in vegetables, diet low in fruit and diet low in whole grains both the Nurses’ Health Study and the Health Professionals Follow-up Study, cohorts with relatively little confounding by socioeconomic status or health care access (screening, treatment), found similar results regardless of the correction of confounding factors.

Furthermore, we must bear in mind that the only dietary risks included were those that were sufficiently described in the literature in terms of causal effect sizes. However, as the effect of other dietary risks on CVDs cannot be excluded, further risk-outcome pairs should be included in future iterations of the GBD CRA framework as far as an aetiologic pathway is observed and epidemiologically confirmed. Potential nutrients with a purported protective effect on CVDs include flavonoids (in particular carotenoids such as astaxanthin, lutein, zeaxanthin and β-cryptoxanthin) [30], vitamin K [31], selenium [32] and vitamin D due to proposed anti-atherosclerotic effects. However, in case of vitamin D a blood pressure lowering effect was only observed in suboptimally supplied individuals (< 50 nmol/L) who received monthly high doses (100,000 UI) for more than a year. [33, 34]. Flavonoids are bioactive compounds that occur naturally in many plants [35]. They likely have protective effects against vascular endothelial cell dysfunction, which is a key event in the aetiological pathway of cardio- and cerebrovascular diseases [36–38]. Although meta-analyses of randomized intervention studies are scarce [39], several meta-analyses of prospective cohort studies revealed an inverse relation between flavonoid consumption and CVDs.

Although substantial progress has been made in the last 26 years to decrease diet-related cardiovascular deaths in the WHO European Region in relative and absolute terms, the last six years have brought an increase in the absolute death numbers for Western Europe and Central Asia. In 2016, optimized dietary patterns could have prevented roughly one in every five premature cardiovascular deaths (22%), with this percentage varying considerably among the countries and regions (Eastern Europe: 32%, Central Europe: 26%, Central Asia: 24%, Western Europe: 14%, EU-28: 17%). Future policy and health interventions will need to be tailor-made and context-specific, involving relevant stakeholders (agriculture, food industry, retailing and the gastronomy sector), to address this issue properly. Distinguishing between primordial, primary and secondary dietary prevention Yu et al. [40] recommends several suitable actions to overcome diet-related CVD deaths. Regular monitoring should surveil the progress towards more health-promoting food environments.

## Electronic supplementary material

Below is the link to the electronic supplementary material.

Supplementary material 1 (DOCX 7585 kb)
