# Supplementary material for: Cardiovascular mortality attributable to dietary risk factors in 51 countries in the WHO European Region from 1990 to 2016: a systematic analysis of the Global Burden of Disease Study
Source: Eur J Epidemiol. 2018 Dec 14;34(1):37–55. doi: 10.1007/s10654-018-0473-x (PMC6325999; doi:10.1007/s10654-018-0473-x)
Supplement: Supplementary file 1 — Supplementary material 1 (DOCX 7585 kb) [file 10654_2018_473_MOESM1_ESM.docx]

**Supporting Material**

**Cardiovascular mortality attributable to dietary risk factors in 51 countries in the WHO European Region from 1990 to 2016: A Systematic Analysis of the Global Burden of Disease Study**

**Table of Contents**

List of Abbreviations… 6

Modelling approach (overview)… 8

Risk exposure data and modelling strategy… 9

Relative risks, population attributable fractions and mediation factors… 10

[Table 1 Considered Countries in the WHO European Region 8](#_Toc530058062)

[Table 2 Relative risks for diet-related cardiovascular diseases for both sexes by age, risk and outcome (incl. uncertainty intervals) 12](#_Toc530058063)

[Table 3 Cause and risk factor specific mediation factors (incl. uncertainty intervals) 14](#_Toc530058064)

[Table 4 Citation of the epidemiological studies used to evaluate the causal relationship between dietary risk-outcome pairs 17](#_Toc530058065)

[Table 5 Death rate (age-standardized), number of deaths of cardiovascular diseases (CVDs) attributable to dietary risks from 1990 to 2016 (including 95% uncertainty interval) 20](#_Toc530058066)

[Figure 1 Diet-related CVD deaths from 1990 to 2016 in Albania 32](#_Toc530058067)

[Figure 2 Age- and gender-specific diet-related CVD deaths (bars) and share of diet-related on total CVD deaths (lines) from 1990 to 2016 in Albania 32](#_Toc530058068)

[Figure 3 Diet-related CVD deaths from 1990 to 2016 in Andorra 33](#_Toc530058069)

[Figure 4 Age- and gender-specific diet-related CVD deaths (bars) and share of diet-related on total CVD deaths (lines) from 1990 to 2016 in Andorra 33](#_Toc530058070)

[Figure 5 Diet-related CVD deaths from 1990 to 2016 in Armenia 34](#_Toc530058071)

[Figure 6 Age- and gender-specific diet-related CVD deaths (bars) and share of diet-related on total CVD deaths (lines) from 1990 to 2016 in Armenia 34](#_Toc530058072)

[Figure 7 Diet-related CVD deaths from 1990 to 2016 in Austria 35](#_Toc530058073)

[Figure 8 Age- and gender-specific diet-related CVD deaths (bars) and share of diet-related on total CVD deaths (lines) from 1990 to 2016 in Austria 35](#_Toc530058074)

[Figure 9 Diet-related CVD deaths from 1990 to 2016 in Azerbaijan 36](#_Toc530058075)

[Figure 10 Age- and gender-specific diet-related CVD deaths (bars) and share of diet-related on total CVD deaths (lines) from 1990 to 2016 in Azerbaijan 36](#_Toc530058076)

[Figure 11 Diet-related CVD deaths from 1990 to 2016 in Belarus 37](#_Toc530058077)

[Figure 12 Age- and gender-specific diet-related CVD deaths (bars) and share of diet-related on total CVD deaths (lines) from 1990 to 2016 in Belarus 37](#_Toc530058078)

[Figure 13 Diet-related CVD deaths from 1990 to 2016 in Belgium 38](#_Toc530058079)

[Figure 14 Age- and gender-specific diet-related CVD deaths (bars) and share of diet-related on total CVD deaths (lines) from 1990 to 2016 in Belgium 38](#_Toc530058080)

[Figure 15 Diet-related CVD deaths from 1990 to 2016 in Bosnia and Herzegovina 39](#_Toc530058081)

[Figure 16 Age- and gender-specific diet-related CVD deaths (bars) and share of diet-related on total CVD deaths (lines) from 1990 to 2016 in Bosnia and Herzegovina 39](#_Toc530058082)

[Figure 17 Diet-related CVD deaths from 1990 to 2016 in Bulgaria 40](#_Toc530058083)

[Figure 18 Age- and gender-specific diet-related CVD deaths (bars) and share of diet-related on total CVD deaths (lines) from 1990 to 2016 in Bulgaria 40](#_Toc530058084)

[Figure 19 Diet-related CVD deaths from 1990 to 2016 in Croatia 41](#_Toc530058085)

[Figure 20 Age- and gender-specific diet-related CVD deaths (bars) and share of diet-related on total CVD deaths (lines) from 1990 to 2016 in Croatia 41](#_Toc530058086)

[Figure 21 Diet-related CVD deaths from 1990 to 2016 in Cyprus 42](#_Toc530058087)

[Figure 22 Age- and gender-specific diet-related CVD deaths (bars) and share of diet-related on total CVD deaths (lines) from 1990 to 2016 in Cyprus 42](#_Toc530058088)

[Figure 23 Diet-related CVD deaths from 1990 to 2016 in the Czech Republic 43](#_Toc530058089)

[Figure 24 Age- and gender-specific diet-related CVD deaths (bars) and share of diet-related on total CVD deaths (lines) from 1990 to 2016 in the Czech Republic 43](#_Toc530058090)

[Figure 25 Diet-related CVD deaths from 1990 to 2016 in Denmark 44](#_Toc530058091)

[Figure 26 Age- and gender-specific diet-related CVD deaths (bars) and share of diet-related on total CVD deaths (lines) from 1990 to 2016 in Denmark 44](#_Toc530058092)

[Figure 27 Diet-related CVD deaths from 1990 to 2016 in Estonia 45](#_Toc530058093)

[Figure 28 Age- and gender-specific diet-related CVD deaths (bars) and share of diet-related on total CVD deaths (lines) from 1990 to 2016 in Estonia 45](#_Toc530058094)

[Figure 29 Diet-related CVD deaths from 1990 to 2016 in the EU-28 46](#_Toc530058095)

[Figure 30 Age- and gender-specific diet-related CVD deaths (bars) and share of diet-related on total CVD deaths (lines) from 1990 to 2016 in the EU-28 46](#_Toc530058096)

[Figure 31 Diet-related CVD deaths from 1990 to 2016 in Finland 47](#_Toc530058097)

[Figure 32 Age- and gender-specific diet-related CVD deaths (bars) and share of diet-related on total CVD deaths (lines) from 1990 to 2016 in Finland 47](#_Toc530058098)

[Figure 33 Diet-related CVD deaths from 1990 to 2016 in France 48](#_Toc530058099)

[Figure 34 Age- and gender-specific diet-related CVD deaths (bars) and share of diet-related on total CVD deaths (lines) from 1990 to 2016 in France 48](#_Toc530058100)

[Figure 35 Diet-related CVD deaths from 1990 to 2016 in Georgia 49](#_Toc530058101)

[Figure 36 Age- and gender-specific diet-related CVD deaths (bars) and share of diet-related on total CVD deaths (lines) from 1990 to 2016 in Georgia 49](#_Toc530058102)

[Figure 37 Diet-related CVD deaths from 1990 to 2016 in Germany 50](#_Toc530058103)

[Figure 38 Age- and gender-specific diet-related CVD deaths (bars) and share of diet-related on total CVD deaths (lines) from 1990 to 2016 in Germany 50](#_Toc530058104)

[Figure 39 Diet-related CVD deaths from 1990 to 2016 in Greece 51](#_Toc530058105)

[Figure 40 Age- and gender-specific diet-related CVD deaths (bars) and share of diet-related on total CVD deaths (lines) from 1990 to 2016 in Greece 51](#_Toc530058106)

[Figure 41 Diet-related CVD deaths from 1990 to 2016 in Hungary 52](#_Toc530058107)

[Figure 42 Age- and gender-specific diet-related CVD deaths (bars) and share of diet-related on total CVD deaths (lines) from 1990 to 2016 in Hungary 52](#_Toc530058108)

[Figure 43 Diet-related CVD deaths from 1990 to 2016 in Iceland 53](#_Toc530058109)

[Figure 44 Age- and gender-specific diet-related CVD deaths (bars) and share of diet-related on total CVD deaths (lines) from 1990 to 2016 in Iceland 53](#_Toc530058110)

[Figure 45 Diet-related CVD deaths from 1990 to 2016 in Ireland 54](#_Toc530058111)

[Figure 46 Age- and gender-specific diet-related CVD deaths (bars) and share of diet-related on total CVD deaths (lines) from 1990 to 2016 in Ireland 54](#_Toc530058112)

[Figure 47 Diet-related CVD deaths from 1990 to 2016 in Israel 55](#_Toc530058113)

[Figure 48 Age- and gender-specific diet-related CVD deaths (bars) and share of diet-related on total CVD deaths (lines) from 1990 to 2016 in Israel 55](#_Toc530058114)

[Figure 49 Diet-related CVD deaths from 1990 to 2016 in Italy 56](#_Toc530058115)

[Figure 50 Age- and gender-specific diet-related CVD deaths (bars) and share of diet-related on total CVD deaths (lines) from 1990 to 2016 in Italy 56](#_Toc530058116)

[Figure 51 Diet-related CVD deaths from 1990 to 2016 in Kazakhstan 57](#_Toc530058117)

[Figure 52 Age- and gender-specific diet-related CVD deaths (bars) and share of diet-related on total CVD deaths (lines) from 1990 to 2016 in Kazakhstan 57](#_Toc530058118)

[Figure 53 Diet-related CVD deaths from 1990 to 2016 in Kyrgyzstan 58](#_Toc530058119)

[Figure 54 Age- and gender-specific diet-related CVD deaths (bars) and share of diet-related on total CVD deaths (lines) from 1990 to 2016 in Kyrgyzstan 58](#_Toc530058120)

[Figure 55 Diet-related CVD deaths from 1990 to 2016 in Latvia 59](#_Toc530058121)

[Figure 56 Age- and gender-specific diet-related CVD deaths (bars) and share of diet-related on total CVD deaths (lines) from 1990 to 2016 in Latvia 59](#_Toc530058122)

[Figure 57 Diet-related CVD deaths from 1990 to 2016 in Lithuania 60](#_Toc530058123)

[Figure 58 Age- and gender-specific diet-related CVD deaths (bars) and share of diet-related on total CVD deaths (lines) from 1990 to 2016 in Lithuania 60](#_Toc530058124)

[Figure 59 Diet-related CVD deaths from 1990 to 2016 in Luxembourg 61](#_Toc530058125)

[Figure 60 Age- and gender-specific diet-related CVD deaths (bars) and share of diet-related on total CVD deaths (lines) from 1990 to 2016 in Luxembourg 61](#_Toc530058126)

[Figure 61 Diet-related CVD deaths from 1990 to 2016 in Macedonia 62](#_Toc530058127)

[Figure 62 Age- and gender-specific diet-related CVD deaths (bars) and share of diet-related on total CVD deaths (lines) from 1990 to 2016 in Macedonia 62](#_Toc530058128)

[Figure 63 Diet-related CVD deaths from 1990 to 2016 in Malta 63](#_Toc530058129)

[Figure 64 Age- and gender-specific diet-related CVD deaths (bars) and share of diet-related on total CVD deaths (lines) from 1990 to 2016 in Malta 63](#_Toc530058130)

[Figure 65 Diet-related CVD deaths from 1990 to 2016 in Moldova 64](#_Toc530058131)

[Figure 66 Age- and gender-specific diet-related CVD deaths (bars) and share of diet-related on total CVD deaths (lines) from 1990 to 2016 in Moldova 64](#_Toc530058132)

[Figure 67 Diet-related CVD deaths from 1990 to 2016 in Montenegro 65](#_Toc530058133)

[Figure 68 Age- and gender-specific diet-related CVD deaths (bars) and share of diet-related on total CVD deaths (lines) from 1990 to 2016 in Montenegro 65](#_Toc530058134)

[Figure 69 Diet-related CVD deaths from 1990 to 2016 in the Netherlands 66](#_Toc530058135)

[Figure 70 Age- and gender-specific diet-related CVD deaths (bars) and share of diet-related on total CVD deaths (lines) from 1990 to 2016 in the Netherlands 66](#_Toc530058136)

[Figure 71 Diet-related CVD deaths from 1990 to 2016 in Norway 67](#_Toc530058137)

[Figure 72 Age- and gender-specific diet-related CVD deaths (bars) and share of diet-related on total CVD deaths (lines) from 1990 to 2016 in Norway 67](#_Toc530058138)

[Figure 73 Diet-related CVD deaths from 1990 to 2016 in Poland 68](#_Toc530058139)

[Figure 74 Age- and gender-specific diet-related CVD deaths (bars) and share of diet-related on total CVD deaths (lines) from 1990 to 2016 in Poland 68](#_Toc530058140)

[Figure 75 Diet-related CVD deaths from 1990 to 2016 in Portugal 69](#_Toc530058141)

[Figure 76 Age- and gender-specific diet-related CVD deaths (bars) and share of diet-related on total CVD deaths (lines) from 1990 to 2016 in Portugal 69](#_Toc530058142)

[Figure 77 Diet-related CVD deaths from 1990 to 2016 in Romania 70](#_Toc530058143)

[Figure 78 Age- and gender-specific diet-related CVD deaths (bars) and share of diet-related on total CVD deaths (lines) from 1990 to 2016 in Romania 70](#_Toc530058144)

[Figure 79 Diet-related CVD deaths from 1990 to 2016 in Russia 71](#_Toc530058145)

[Figure 80 Age- and gender-specific diet-related CVD deaths (bars) and share of diet-related on total CVD deaths (lines) from 1990 to 2016 in Russia 71](#_Toc530058146)

[Figure 81 Diet-related CVD deaths from 1990 to 2016 in Serbia 72](#_Toc530058147)

[Figure 82 Age- and gender-specific diet-related CVD deaths (bars) and share of diet-related on total CVD deaths (lines) from 1990 to 2016 in Serbia 72](#_Toc530058148)

[Figure 83 Diet-related CVD deaths from 1990 to 2016 in Slovakia 73](#_Toc530058149)

[Figure 84 Age- and gender-specific diet-related CVD deaths (bars) and share of diet-related on total CVD deaths (lines) from 1990 to 2016 in Slovakia 73](#_Toc530058150)

[Figure 85 Diet-related CVD deaths from 1990 to 2016 in Slovenia 74](#_Toc530058151)

[Figure 86 Age- and gender-specific diet-related CVD deaths (bars) and share of diet-related on total CVD deaths (lines) from 1990 to 2016 in Slovenia 74](#_Toc530058152)

[Figure 87 Diet-related CVD deaths from 1990 to 2016 in Spain 75](#_Toc530058153)

[Figure 88 Age- and gender-specific diet-related CVD deaths (bars) and share of diet-related on total CVD deaths (lines) from 1990 to 2016 in Spain 75](#_Toc530058154)

[Figure 89 Diet-related CVD deaths from 1990 to 2016 in Sweden 76](#_Toc530058155)

[Figure 90 Age- and gender-specific diet-related CVD deaths (bars) and share of diet-related on total CVD deaths (lines) from 1990 to 2016 in Sweden 76](#_Toc530058156)

[Figure 91 Diet-related CVD deaths from 1990 to 2016 in Switzerland 77](#_Toc530058157)

[Figure 92 Age- and gender-specific diet-related CVD deaths (bars) and share of diet-related on total CVD deaths (lines) from 1990 to 2016 in Switzerland 77](#_Toc530058158)

[Figure 93 Diet-related CVD deaths from 1990 to 2016 in Tajikistan 78](#_Toc530058159)

[Figure 94 Age- and gender-specific diet-related CVD deaths (bars) and share of diet-related on total CVD deaths (lines) from 1990 to 2016 in Tajikistan 78](#_Toc530058160)

[Figure 95 Diet-related CVD deaths from 1990 to 2016 in Turkey 79](#_Toc530058161)

[Figure 96 Age- and gender-specific diet-related CVD deaths (bars) and share of diet-related on total CVD deaths (lines) from 1990 to 2016 in Turkey 79](#_Toc530058162)

[Figure 97 Diet-related CVD deaths from 1990 to 2016 in Turkmenistan 80](#_Toc530058163)

[Figure 98 Age- and gender-specific diet-related CVD deaths (bars) and share of diet-related on total CVD deaths (lines) from 1990 to 2016 in Turkmenistan 80](#_Toc530058164)

[Figure 99 Diet-related CVD deaths from 1990 to 2016 in Ukraine 81](#_Toc530058165)

[Figure 100 Age- and gender-specific diet-related CVD deaths (bars) and share of diet-related on total CVD deaths (lines) from 1990 to 2016 in Ukraine 81](#_Toc530058166)

[Figure 101 Diet-related CVD deaths from 1990 to 2016 in the United Kingdom 82](#_Toc530058167)

[Figure 102 Age- and gender-specific diet-related CVD deaths (bars) and share of diet-related on total CVD deaths (lines) from 1990 to 2016 in the United Kingdom 82](#_Toc530058168)

[Figure 103 Diet-related CVD deaths from 1990 to 2016 in Uzbekistan 83](#_Toc530058169)

[Figure 104 Age- and gender-specific diet-related CVD deaths (bars) and share of diet-related on total CVD deaths (lines) from 1990 to 2016 in Uzbekistan 83](#_Toc530058170)

**List of abbreviations**

BMI: body‐mass index

CRA: comparative risk assessment

CVD: cardiovascular disease

DALY: disability‐adjusted life‐year

DisMod-MR 2.1: Disease Modelling Bayesian Meta-Regression tool, version 2.1FAO: Food and Agriculture Organization

FPG: fasting plasma glucose

GATHER: Guidelines for Accurate and Transparent Health Estimates Reporting

GBD: Global Burden of Disease

IHD: ischaemic heart disease

LDI: lag distributed income per capita

LMIC: low and middle‐income countries

LRI: lower respiratory infection

MCMC: Markov Chain Monte Carlo simulations

MDG: Millennium Development Goal

MoM: method of moments

NCD: non‐communicable disease

PAF: population attributable fraction

PSC: Prospective Cohort Study

PUFA: polyunsaturated fatty acid

RCT: randomised controlled trial

RMSE: root mean square error

RR: relative risk

SBP: systolic blood pressure

SD: standard deviation

SDG: Sustainable Development Goal

SDI: Socio‐demographic Index

SEV: summary exposure value

SSB: sugar‐sweetened beverages

ST‐GPR: spatiotemporal Gaussian process regression

TMREL: theoretical minimum‐risk exposure level

UI: uncertainty interval

UR: uncertainty ratio

WCRF: World Cancer Research Fund

WHO: World Health Organization

YLD: years lived with disability

YLL: years of life lost

Table 1 Considered Countries in the WHO European Region

| **Region** | **Countries** |
| --- | --- |
| Western Europe | Andorra, Austria, Belgium, Cyprus, Denmark, Finland, France, Germany, Greece, Iceland, Ireland, Israel, Italy, Luxembourg, Malta, Netherlands, Norway, Portugal, Spain, Sweden, Switzerland, United Kingdom |
| Central Europe | Albania, Bosnia and Herzegovina, Bulgaria, Croatia, Czech Republic, Hungary, Macedonia, Montenegro, Poland, Romania, Serbia, Slovakia, Slovenia |
| Eastern Europe | Belarus, Estonia, Latvia, Lithuania, Moldova, Russia, Ukraine |
| Central Asia + Turkey | Armenia, Azerbaijan, Georgia, Kazakhstan, Kyrgyzstan, Tajikistan, Turkey, Turkmenistan, Uzbekistan |
| EU-28 | Austria, Belgium, Bulgaria, Croatia, Cyprus, Czech Republic, Denmark, Estonia, Finland, France, Germany, Greece, Hungary, Ireland, Italy, Latvia, Lithuania, Luxembourg, Malta, the Netherlands, Poland, Portugal, Romania, Slovakia, Slovenia, Spain, Sweden, United Kingdom |

**Modelling approach (overview)**

The comparative risk assessment framework was developed by Murray and Lopez^[[1]](#footnote-1)^ who established a causal web of hierarchically organised risks or causes that contribute to health outcomes, which allows for quantification of risks or causes at any level in the framework. In GBD 2016, as in previous iterations of the GBD study, we evaluated a set of behavioural, environmental and occupational, and metabolic risks, where risk-outcome pairs were included based on established evidence rules (see the Supporting Material of GBD 2016 capstone paper^[[2]](#footnote-2)^). These risks were organised in four hierarchical Levels. At each Level of risk, we evaluated whether risk combinations were additive, multiplicative, or shared common pathways for intervention. This approach allows the quantification of the proportion of risk-attributable burden shared with another risk or combination of risks and the measurement of potential overlaps between behavioural, environmental and occupational, and metabolic risks. Within the GBD two principle types of modelling approaches are applied, a Bayesian meta-regression model (DisMod-MR 2.1) and a spatiotemporal Gaussian process regression model (ST-GPR), to pool data from different sources, control and adjust for bias in data, and incorporate other types of information such as countrylevel covariates. DisMod-MR 2.1 and ST-GPR are mixed effect models that borrow information across age, time, and locations to synthesise multiple sources of data into unified estimates of levels and trends. A detailed description of the likelihood used for estimation, and a full description of improvements made for DisMod-MR 2.1 are described by Vos et al. (2016).^[[3]](#footnote-3)^

**Risk exposure data and modelling strategy**

For the risk factor exposure estimation of dietary risks we used a spatio-temporal Gaussian process regression (ST-GPR) framework to estimate the intake of each dietary factor by age, sex, country, and year. In GBD 2016, for all dietary factors other than sodium, we considered data from 24-hour diet recall as the gold standard. For sodium, the 24-hour urinary sodium was considered as gold standard. To estimate the 24-hour urinary sodium based on dietary sodium, we performed an adjustment between these two types of data. For the remaining diet-related risk factors we used dietary data from multiple sources including nationally and sub-nationally representative nutrition surveys, household budget surveys, accounts of national sales, and United Nations FAO Food Balance Sheets and Supply and Utilization Accounts. Additionally, for trans fatty acids we used data of the availability of hydrogenated vegetable oil in packaged foods. Polyunsaturated and trans fatty acids were modelled as a percent of total dietary energy. We modelled missing country-year data from FAO using a space-time Gaussian process regression and lag-distributed country income as the covariate. For each dietary factor, we estimated the global age pattern of consumption based on nutrition surveys (i.e., 24-hour diet recall) and applied that age pattern to the FAO data. Substantive changes in input data compared to GBD 2015 are as follows: (a) reextracting data from all nutrition surveys and standardizing the definition of dietary components across sources; (b) incorporating data gathered through a systematic review of literature for each of our dietary risk factors; (c) using sales data for fruit, vegetables, legumes, processed meats, red meats, sugar-sweetened beverages, and milk. To characterize the distribution of each dietary factor at population level, we use an ensemble approach that separately fit 12 distributions for individual level microdata to specific to each data source’s sampled population. The respective goodness of fit of each family was assessed and a weighting scheme was determined to optimize overall fit to the unique distribution of each risk factor. A global mean of the weights for each risk factor’s data sources was created. We then determined the standard deviation of each population’s consumption through a linear regression that captured the relationship between the standard deviation and mean of intake in nationally representative nutrition surveys using 24-hour diet recalls. Then we applied the coefficients of this regression to the outputs of our ST-GPR model to calculate the standard deviation of intake by age, sex, year, and country. We also quantified the within person variation in consumption of each dietary component and adjusted the standard deviations accordingly. We used Dismod-MR 2.1 to pool effect sizes from included studies in the meta-analyses and to generate outcome-specific dose-response curves for each dietary risk factors and mediation factors related to CVDs.

**Relative risks, population attributable fractions and mediation factors**

We obtained the relative risk of each disease endpoint per serving of the dietary components from recent dose-response meta-analyses of prospective observational studies, and where available randomized controlled trials (RCTs). In GBD 2016, we specifically updated the relative risks for the relationship between a diet low in legumes and ischemic heart disease, which is now being considered distinctly as opposed to being placed within the category of vegetables. Considering the well-established age trend of the relative risks of metabolic risk factors for cardiovascular disease, we conducted a literature review to identify the most important metabolic mediators for each dietary factor and used the age trend of the relative risk of that mediator(s) and the disease endpoint to estimate the age-specific relative risk for each dietary factors (Appendix Table 2).

To avoid double counting of risk related-disease burdens when the effects of joint risk factors are aggregated, we included corresponding mediation factors offsetting the overlaps of disease burdens between multiple risk factors. Therefore, we first prepared a list of possible mediation-pairs especially between behavioural risks and metabolic risk factors with cardiometabolic outcomes based on Danaei et al (2013).^[[4]](#footnote-4)^ Danaei et al. (2013) assumed that part of the effect of BMI on ischemic heart disease (IHD) is through high systolic blood pressure (SBP), cholesterol and fasting plasma glucose (FPG). The proportion of the BMI effect that can be explained by other metabolic risk factors is the amount of mediation. The difference between the crude RR of BMI on IHD with the RR adjusted for SBP, FPG, and cholesterol reflects the amount of BMI effect on IHD that is mediated and already included in SBP, FPG, and cholesterol. Some mediation factors equal 1 where the whole effect was calculated through other risk factor, e.g. the effect of sugar-sweetened beverages through BMI or salt through SBP, or when we assumed other risk factors are sources of the exposure, for example, fibre is provided by consuming fruit, vegetable, and whole grains. In case of more than one risk factor for an outcome, we estimated the fraction of risk that was mediated through the other risk. This resulted in a matrix of parameters containing each possible pairing of risk factors included in the GBD 2016 using the formula:

$${PAF}_{Joasgt}=1-\prod_{j=1}^{J} (1-{PAF}_{joasgt}\prod_{i=1}^{J} (1-{MF}_{jio}))$$

where *J* is a set of risk factors for the aggregation; *PAF_joasgt_* is the population attributable fraction for risk 𝑗 for age group 𝑎, sex 𝑠, location 𝑔, and year 𝑡; and *MF_jio_* is the mediation factor for risk 𝑗 mediated through 𝑖 for cause 𝑜. Mediation factors can be found in Appendix Table 3. We kept uncertainty of each parameter by generating and following 1000 draws of the estimates to calculate 1000 draws of the posterior distribution of the mediation factor. We did not include risk mediator pairs if the mediation factor was not significant at 5% level (more than 50 out of 1000 draws were negative). We truncated the mediation factor distribution at 1 where the whole effect of the risk factor on the outcome would be assumed to be through the mediator pathway. Further details can be found in the Supporting Material of the GBD 2016 capstone paper.^[[5]](#footnote-5)^

Table 2 Relative risks for diet-related cardiovascular diseases for both sexes by age, risk and outcome (incl. uncertainty intervals)

| **Risk - Outcome** | **Exposure unit** | **25-29 years** | **30-34 years** | **35-39 years** | **40-44 years** | **45-49 years** | **50-54 years** | **55-59 years** | **60-64 years** | **65-69 years** | **70-74 years** | **75-79 years** | **80-84 years** | **85-89 years** | **90-94 years** | **95+**  **years** |
| --- | --- | --- | --- | --- | --- | --- | --- | --- | --- | --- | --- | --- | --- | --- | --- | --- |
| Diet low in fruits | | | | | | | | | | | | | | | | |
| Ischaemic heart disease | 100 g/day | 1.254  (1.083 - 1.442) | 1.209  (1.070 - 1.361) | 1.159  (1.054 - 1.271) | 1.131  (1.045 - 1.221) | 1.125  (1.043 - 1.211) | 1.114  (1.039 - 1.193) | 1.099  (1.034 - 1.167) | 1.087  (1.030 - 1.146) | 1.078  (1.027 - 1.13) | 1.070  (1.025 - 1.117) | 1.064  (1.022 - 1.106) | 1.057  (1.020 - 1.095) | 1.057  (1.020 - 1.095) | 1.057  (1.020 - 1.095) | 1.057  (1.020 - 1.095) |
| Ischaemic stroke | 100 g/day | 2.024  (1.465 - 2.818) | 1.834  (1.390 - 2.444) | 1.621  (1.301 - 2.043) | 1.480  (1.239 - 1.787) | 1.403  (1.204 - 1.653) | 1.333  (1.171 - 1.533) | 1.272  (1.142 - 1.432) | 1.222  (1.116 - 1.348) | 1.181  (1.096 - 1.283) | 1.145  (1.078 - 1.225) | 1.114  (1.061 - 1.175) | 1.054  (1.029 - 1.082) | 1.054  (1.029 - 1.082) | 1.054  (1.029 - 1.082) | 1.054  (1.029 - 1.082) |
| Hemorrhagic stroke | 100 g/day | 1.688  (1.319 - 2.182) | 1.576  (1.273 - 1.972) | 1.444  (1.215 - 1.732) | 1.365  (1.18 - 1.595) | 1.336  (1.167 - 1.544) | 1.300  (1.150 - 1.483) | 1.260  (1.131 - 1.415) | 1.226  (1.115 - 1.358) | 1.193  (1.099 - 1.305) | 1.164  (1.084 - 1.256) | 1.133  (1.069 - 1.207) | 1.065  (1.034 - 1.100) | 1.065  (1.034 - 1.100) | 1.065  (1.034 - 1.100) | 1.065  (1.034 - 1.100) |
| Diet low in vegetables | | | | | | | | | | | | | | | | |
| Ischaemic heart disease | 100 g/day | 1.249  (1.089 - 1.446) | 1.205  (1.074 - 1.362) | 1.154  (1.057 - 1.269) | 1.126  (1.047 - 1.219) | 1.121  (1.045 - 1.210) | 1.111  (1.042 - 1.193) | 1.098  (1.037 - 1.168) | 1.086  (1.032 - 1.148) | 1.077  (1.029 - 1.133) | 1.070  (1.027 - 1.120) | 1.064  (1.024 - 1.109) | 1.057  (1.022 - 1.097) | 1.057  (1.022 - 1.097) | 1.057  (1.022 - 1.097) | 1.057  (1.022 - 1.097) |
| Ischaemic stroke | 100 g/day | 1.249  (1.049 - 1.463) | 1.211  (1.042 - 1.388) | 1.165  (1.033 - 1.300) | 1.132  (1.027 - 1.238) | 1.113  (1.023 - 1.203) | 1.095  (1.020 - 1.170) | 1.079  (1.017 - 1.141) | 1.065  (1.014 - 1.116) | 1.054  (1.012 - 1.096) | 1.044  (1.009 - 1.077) | 1.035  (1.007 - 1.061) | 1.017  (1.004 - 1.029) | 1.017  (1.004 - 1.029) | 1.017  (1.004 - 1.029) | 1.017  (1.004 - 1.029) |
| Hemorrhagic stroke | 100 g/day | 1.177  (1.046 - 1.326) | 1.153  (1.040 - 1.278) | 1.122  (1.032 - 1.220) | 1.102  (1.027 - 1.184) | 1.095  (1.025 - 1.170) | 1.086  (1.023 - 1.153) | 1.075  (1.020 - 1.134) | 1.066  (1.018 - 1.117) | 1.057  (1.015 - 1.101) | 1.049  (1.013 - 1.086) | 1.040  (1.011 - 1.071) | 1.020  (1.005 - 1.035) | 1.020  (1.005 - 1.035) | 1.020  (1.005 - 1.035) | 1.020  (1.005 - 1.035) |
| Diet low in legumes | | | | | | | | | | | | | | | | |
| Ischaemic heart disease | 50 g/day | 1.499  (1.180 - 1.890) | 1.453  (1.166 - 1.801) | 1.388  (1.144 - 1.677) | 1.332  (1.125 - 1.573) | 1.287  (1.110 - 1.490) | 1.237  (1.092 - 1.401) | 1.181  (1.071 - 1.303) | 1.139  (1.055 - 1.230) | 1.111  (1.045 - 1.183) | 1.089  (1.036 - 1.146) | 1.074  (1.030 - 1.120) | 1.101  (1.041 - 1.165) | 1.101  (1.041 - 1.165) | 1.101  (1.041 - 1.165) | 1.101  (1.041 - 1.165) |
| Diet low in whole grains | | | | | | | | | | | | | | | | |
| Ischaemic heart disease | 50 g/day | 1.478  (1.274 - 1.722) | 1.387  (1.225 - 1.578) | 1.285  (1.168 - 1.418) | 1.228  (1.136 - 1.333) | 1.216  (1.129 - 1.313) | 1.194  (1.117 - 1.281) | 1.165  (1.1 - 1.238) | 1.141  (1.086 - 1.203) | 1.125  (1.076 - 1.179) | 1.112  (1.068 - 1.160) | 1.102  (1.062 - 1.145) | 1.097  (1.059 - 1.138) | 1.097  (1.059 - 1.138) | 1.097  (1.059 - 1.138) | 1.097  (1.059 - 1.138) |
| Ischaemic stroke | 50 g/day | 2.075  (1.669 - 2.517) | 1.863  (1.548 - 2.199) | 1.624  (1.406 - 1.849) | 1.466  (1.309 - 1.625) | 1.380  (1.255 - 1.505) | 1.304  (1.206 - 1.401) | 1.241  (1.165 - 1.316) | 1.189  (1.130 - 1.247) | 1.150  (1.104 - 1.195) | 1.117  (1.081 - 1.151) | 1.090  (1.063 - 1.116) | 1.041  (1.029 - 1.053) | 1.041  (1.029 - 1.053) | 1.041  (1.029 - 1.053) | 1.041  (1.029 - 1.053) |
| Hemorrhagic stroke | 50 g/day | 1.596  (1.406 - 1.825) | 1.484  (1.333 - 1.662) | 1.349  (1.244 - 1.471) | 1.276  (1.194 - 1.369) | 1.258  (1.182 - 1.344) | 1.232  (1.165 - 1.309) | 1.201  (1.143 - 1.267) | 1.176  (1.126 - 1.233) | 1.150  (1.108 - 1.198) | 1.128  (1.092 - 1.169) | 1.106  (1.076 - 1.139) | 1.050  (1.036 - 1.065) | 1.050  (1.036 - 1.065) | 1.050  (1.036 - 1.065) | 1.050  (1.036 - 1.065) |
| Diet low in nuts and seeds | | | | | | | | | | | | | | | | |
| Ischaemic heart disease | 4.05 g/day | 1.209  (1.128 - 1.296) | 1.169  (1.105 - 1.239) | 1.124  (1.077 - 1.174) | 1.099  (1.062 - 1.138) | 1.095  (1.060 - 1.133) | 1.088  (1.055 - 1.122) | 1.076  (1.048 - 1.105) | 1.066  (1.042 - 1.092) | 1.059  (1.037 - 1.082) | 1.054  (1.034 - 1.075) | 1.050  (1.032 - 1.069) | 1.046  (1.029 - 1.064) | 1.046  (1.029 - 1.064) | 1.046  (1.029 - 1.064) | 1.046  (1.029 - 1.064) |
| Diet high in processed meat | | | | | | | | | | | | | | | | |
| Ischaemic heart disease | 50 g/day | 2.568  (1.047 - 4.657) | 2.124  (1.038 - 3.478) | 1.720  (1.028 - 2.489) | 1.545  (1.022 - 2.093) | 1.547  (1.022 - 2.097) | 1.520  (1.022 - 2.037) | 1.467  (1.020 - 1.922) | 1.422  (1.018 - 1.826) | 1.386  (1.017 - 1.750) | 1.354  (1.016 - 1.683) | 1.325  (1.015 - 1.622) | 1.252  (1.012 - 1.475) | 1.252  (1.012 - 1.475) | 1.252  (1.012 - 1.475) | 1.252  (1.012 - 1.475) |
| Diet high in sugar-sweetened beverages | | | | | | | | | | | | | | | | |
| Ischaemic heart disease | 2.5 g/day | 1.377  (0.933 - 1.883) | 1.311  (0.943 - 1.717) | 1.232  (0.955 - 1.521) | 1.195  (0.961 - 1.436) | 1.186  (0.963 - 1.413) | 1.172  (0.965 - 1.381) | 1.156  (0.968 - 1.343) | 1.14  (0.971 - 1.306) | 1.124  (0.974 - 1.27) | 1.11  (0.977 - 1.238) | 1.095  (0.98 - 1.205) | 1.067  (0.985 - 1.143) | 1.067  (0.985 - 1.143) | 1.067  (0.985 - 1.143) | 1.067  (0.985 - 1.143) |

| **Risk - Outcome** | **Exposure unit / Category** | **25-29 years** | **30-34 years** | **35-39 years** | **40-44 years** | **45-49 years** | **50-54 years** | **55-59 years** | **60-64 years** | **65-69 years** | **70-74 years** | **75-79 years** | **80-84 years** | **85-89 years** | **90-94 years** | **95+**  **years** |
| --- | --- | --- | --- | --- | --- | --- | --- | --- | --- | --- | --- | --- | --- | --- | --- | --- |
| Diet low in fibre | | | | | | | | | | | | | | | | |
| Ischaemic heart disease | 20 g/day | 1.688  (1.415 - 2.028) | 1.622  (1.379 - 1.922) | 1.529  (1.326 - 1.776) | 1.45  (1.280 - 1.654) | 1.387  (1.243 - 1.558) | 1.318  (1.202 - 1.455) | 1.242  (1.156 - 1.342) | 1.184  (1.119 - 1.258) | 1.147  (1.096 - 1.205) | 1.118  (1.077 - 1.163) | 1.097  (1.064 - 1.135) | 1.133  (1.087 - 1.185) | 1.133  (1.087 - 1.185) | 1.133  (1.087 - 1.185) | 1.133  (1.087 - 1.185) |
| Diet low in seafood omega-3 fatty acids | | | | | | | | | | | | | | | | |
| Ischaemic heart disease | 100 mg/day | 1.291  (1.109 - 1.505) | 1.249  (1.094 - 1.428) | 1.199  (1.077 - 1.338) | 1.173  (1.067 - 1.293) | 1.165  (1.064 - 1.279) | 1.154  (1.06 - 1.260) | 1.140  (1.055 - 1.235) | 1.126  (1.050 - 1.211) | 1.113  (1.045 - 1.189) | 1.101  (1.040 - 1.167) | 1.088  (1.035 - 1.145) | 1.062  (1.025 - 1.102) | 1.062  (1.025 - 1.102) | 1.062  (1.025 - 1.102) | 1.062  (1.025 - 1.102) |
| Diet low in polyunsaturated fatty acids | | | | | | | | | | | | | | | | |
| Ischaemic heart disease | 5% energy/day | 1.267  (1.098 - 1.452) | 1.211  (1.079 - 1.352) | 1.148  (1.056 - 1.244) | 1.114  (1.044 - 1.186) | 1.111  (1.043 - 1.181) | 1.101  (1.039 - 1.165) | 1.086  (1.033 - 1.140) | 1.075  (1.029 - 1.121) | 1.068  (1.026 - 1.110) | 1.063  (1.025 - 1.102) | 1.060  (1.024 - 1.097) | 1.063  (1.025 - 1.102) | 1.063  (1.025 - 1.102) | 1.063  (1.025 - 1.102) | 1.063  (1.025 - 1.102) |
| Diet high in trans fatty acids | | | | | | | | | | | | | | | | |
| Ischaemic heart disease | 2% energy/day | 1.901  (1.591 - 2.275) | 1.775  (1.514 - 2.085) | 1.615  (1.415 - 1.848) | 1.517  (1.352 - 1.707) | 1.461  (1.316 - 1.627) | 1.396  (1.274 - 1.535) | 1.323  (1.225 - 1.433) | 1.264  (1.186 - 1.352) | 1.222  (1.157 - 1.294) | 1.186  (1.132 - 1.246) | 1.158  (1.112 - 1.207) | 1.150  (1.107 - 1.197) | 1.150  (1.107 - 1.197) | 1.150  (1.107 - 1.197) | 1.150  (1.107 - 1.197) |
| Diet high in sodium * | | | | | | | | | | | | | | | | |
|  | Non-Black, Non-Hypertensive | -1.366  (-1.937 - -0.795) | -1.882  (-2.434 - -1.330) | -2.397  (-2.967 - -1.828) | -2.913  (-3.533 - -2.292) | -3.428  (-4.126 - -2.730) | -3.944  (-4.738 - -3.150) | -4.459  (-5.362 - -3.556) | -4.975  (-5.995 - -3.954) | -5.490  (-6.634 - -4.347) | -5.490  (-6.634 - -4.347) | -5.490  (-6.634 - -4.347) | -5.490  (-6.634 - -4.347) | -5.490  (-6.634 - -4.347) | -5.490  (-6.634 - -4.347) | -5.490  (-6.634 - -4.347) |
|  | Non-Black, Hypertensive | -3.300  (-4.147 - -2.454) | -3.816  (-4.547 - -3.085) | -4.331  (-4.959 - -3.704) | -4.847  (-5.389 - -4.305) | -5.363  (-5.848 - -4.877) | -5.878  (-6.346 - -5.411) | -6.394  (-6.886 - -5.901) | -6.909  (-7.464 - -6.354) | -7.425  (-8.069 - -6.781) | -7.425  (-8.069 - -6.781) | -7.425  (-8.069 - -6.781) | -7.425  (-8.069 - -6.781) | -7.425  (-8.069 - -6.781) | -7.425  (-8.069 - -6.781) | -7.425  (-8.069 - -6.781) |
|  | Black, Non-Hypertensive | -3.910  (-5.065 - -2.755) | -4.426  (-5.564 - -3.287) | -4.941  (-6.081 - -3.802) | -5.457  (-6.616 - -4.298) | -5.972  (-7.168 - -4.777) | -6.488  (-7.735 - -5.241) | -7.004  (-8.316 - -5.691) | -7.519  (-8.909 - -6.129) | -8.035  (-9.512 - -6.557) | -8.035  (-9.512 - -6.557) | -8.035  (-9.512 - -6.557) | -8.035  (-9.512 - -6.557) | -8.035  (-9.512 - -6.557) | -8.035  (-9.512 - -6.557) | -8.035  (-9.512 - -6.557) |
|  | Black, Hypertensive | -5.844  (-7.222 - -4.467) | -6.360  (-7.663 - -5.057) | -6.876  (-8.117 - -5.635) | -7.391  (-8.584 - -6.198) | -7.907  (-9.068 - -6.745) | -8.422  (-9.569 - -7.275) | -8.938  (-10.088 - -7.788) | -9.453  (-10.624 - -8.282) | -9.969  (-11.178 - -8.760) | -9.969  (-11.178 - -8.760) | -9.969  (-11.178 - -8.760) | -9.969  (-11.178 - -8.760) | -9.969  (-11.178 - -8.760) | -9.969  (-11.178 - -8.760) | -9.969  (-11.178 - -8.760) |

* For diet high in sodium the shifts reported are based on mediation through high systolic blood pressure.

Table 3 Cause and risk factor specific mediation factors (incl. uncertainty intervals)

| **№** | **Cause** | **Risk Factor** | **Mediator** | **Mediation Factor** | **UI** |
| --- | --- | --- | --- | --- | --- |
| 1 | Ischemic heart disease | Diet low in whole grains | High body‐mass index | 0.04 | 0.03 - 0.05 |
| 2 | Hemorrhagic stroke | Diet low in whole grains | High body‐mass index | 0.03 | 0.02 - 0.04 |
| 3 | Ischemic stroke | Diet low in whole grains | High body‐mass index | 0.04 | 0.03 - 0.05 |
| 4 | Ischemic heart disease | Diet low in whole grains | High total cholesterol | 0.39 | 0.17 - 0.54 |
| 5 | Ischemic stroke | Diet low in whole grains | High total cholesterol | 0.15 | 0.04 - 0.24 |
| 6 | Ischemic heart disease | Diet low in vegetables | High total cholesterol | 0.04 | 0.03 - 0.05 |
| 7 | Ischemic stroke | Diet low in vegetables | High total cholesterol | 0.08 | 0.03 - 0.13 |
| 8 | Ischemic heart disease | Diet low in vegetables | High fasting plasma glucose | 0.05 | 0.01 - 0.09 |
| 9 | Hemorrhagic stroke | Diet low in vegetables | High fasting plasma glucose | 0.08 | 0.03 - 0.12 |
| 10 | Ischemic stroke | Diet low in vegetables | High fasting plasma glucose | 0.08 | 0.03 - 0.12 |
| 11 | Ischemic heart disease | Diet low in vegetables | High systolic blood pressure | 0.04 | 0.03 - 0.05 |
| 12 | Hemorrhagic stroke | Diet low in vegetables | High systolic blood pressure | 0.04 | 0.02 - 0.05 |
| 13 | Ischemic stroke | Diet low in vegetables | High systolic blood pressure | 0.03 | 0.02 - 0.04 |
| 14 | Ischemic heart disease | Diet low in seafood omega‐3 fatty acids | High body‐mass index | 0.03 | 0.02 - 0.05 |
| 15 | Ischemic heart disease | Diet low in seafood omega‐3 fatty acids | High systolic blood pressure | 0.01 | 0 - 0.02 |
| 16 | Ischemic heart disease | Diet low in polyunsaturated fatty acids | High fasting plasma glucose | 0.57 | 0.39 - 0.77 |
| 17 | Ischemic heart disease | Diet low in nuts and seeds | High total cholesterol | 0.13 | 0 - 0.24 |
| 18 | Ischemic heart disease | Diet low in nuts and seeds | High fasting plasma glucose | 0.03 | 0.02 - 0.06 |
| 19 | Ischemic heart disease | Diet low in fruits | High total cholesterol | 0.06 | 0.05 - 0.08 |
| 20 | Ischemic stroke | Diet low in fruits | High total cholesterol | 0.05 | 0.04 - 0.06 |
| 21 | Ischemic stroke | Diet low in fruits | High fasting plasma glucose | 0.05 | 0.04 - 0.06 |
| 22 | Ischemic heart disease | Diet low in fruits | High systolic blood pressure | 0.06 | 0.05 - 0.08 |
| 23 | Hemorrhagic stroke | Diet low in fruits | High systolic blood pressure | 0.02 | 0.02 - 0.03 |
| 24 | Ischemic stroke | Diet low in fruits | High systolic blood pressure | 0.05 | 0.04 - 0.06 |
| 25 | Ischemic heart disease | Diet low in fiber | Diet low in whole grains | 1 | 1 - 1 |
| 26 | Ischemic heart disease | Diet low in fiber | Diet low in vegetables | 1 | 1 - 1 |
| 27 | Ischemic heart disease | Diet low in fiber | Diet low in fruits | 1 | 1 - 1 |
| 28 | Ischemic heart disease | Diet high in trans fatty acids | High total cholesterol | 0.15 | 0.02 - 0.24 |
| 29 | Ischemic heart disease | Diet high in trans fatty acids | High systolic blood pressure | 0.15 | 0.02 - 0.24 |
| 30 | Ischemic heart disease | Diet high in sugar‐sweetened beverages | High body‐mass index | 1 | 1 - 1 |
| 31 | Hemorrhagic stroke | Diet high in sugar‐sweetened beverages | High body‐mass index | 1 | 1 - 1 |
| 32 | Ischemic stroke | Diet high in sugar‐sweetened beverages | High body‐mass index | 1 | 1 - 1 |
| 33 | Atrial fibrillation and flutter | Diet high in sugar‐sweetened beverages | High body‐mass index | 1 | 1 - 1 |
| 34 | Ischemic heart disease | Diet high in sugar‐sweetened beverages | High total cholesterol | 0.1 | 0.05 - 0.15 |
| 35 | Ischemic stroke | Diet high in sugar‐sweetened beverages | High total cholesterol | 0.03 | 0 - 0.08 |
| 36 | Atrial fibrillation and flutter | Diet high in sugar‐sweetened beverages | High total cholesterol | 0.1 | 0.05 - 0.15 |
| 37 | Cardiomyopathy and myocarditis | Diet high in sugar‐sweetened beverages | High total cholesterol | 0.1 | 0.05 - 0.15 |
| 38 | Peripheral artery disease | Diet high in sugar‐sweetened beverages | High total cholesterol | 0.1 | 0.05 - 0.15 |
| 39 | Ischemic heart disease | Diet high in sugar‐sweetened beverages | High fasting plasma glucose | 0.15 | 0.1 - 0.2 |
| 40 | Hemorrhagic stroke | Diet high in sugar‐sweetened beverages | High fasting plasma glucose | 0.22 | 0.12 - 0.33 |
| 41 | Ischemic stroke | Diet high in sugar‐sweetened beverages | High fasting plasma glucose | 0.22 | 0.12 - 0.31 |
| 42 | Atrial fibrillation and flutter | Diet high in sugar‐sweetened beverages | High fasting plasma glucose | 0.15 | 0.09 - 0.2 |
| 43 | Cardiomyopathy and myocarditis | Diet high in sugar‐sweetened beverages | High fasting plasma glucose | 0.15 | 0.1 - 0.2 |
| 44 | Peripheral artery disease | Diet high in sugar‐sweetened beverages | High fasting plasma glucose | 0.15 | 0.1 - 0.2 |
| 45 | Ischemic heart disease | Diet high in sugar‐sweetened beverages | High systolic blood pressure | 0.31 | 0.28 - 0.34 |
| 46 | Hemorrhagic stroke | Diet high in sugar‐sweetened beverages | High systolic blood pressure | 0.65 | 0.58 - 0.73 |
| 47 | Ischemic stroke | Diet high in sugar‐sweetened beverages | High systolic blood pressure | 0.65 | 0.57 - 0.72 |
| 48 | Atrial fibrillation and flutter | Diet high in sugar‐sweetened beverages | High systolic blood pressure | 0.31 | 0.28 - 0.34 |
| 49 | Cardiomyopathy and myocarditis | Diet high in sugar‐sweetened beverages | High systolic blood pressure | 0.31 | 0.28 - 0.34 |
| 50 | Hypertensive heart disease | Diet high in sugar‐sweetened beverages | High systolic blood pressure | 1 | 1 - 1 |
| 51 | Other cardiovascular and circulatory diseases | Diet high in sugar‐sweetened beverages | High systolic blood pressure | 0.31 | 0.28 - 0.34 |
| 52 | Peripheral artery disease | Diet high in sugar‐sweetened beverages | High systolic blood pressure | 0.31 | 0.28 - 0.34 |
| 53 | Cardiomyopathy and myocarditis | Diet high in sugar‐sweetened beverages | High body‐mass index | 1 | 1 - 1 |
| 54 | Hypertensive heart disease | Diet high in sugar‐sweetened beverages | High body‐mass index | 1 | 1 - 1 |
| 55 | Other cardiovascular and circulatory diseases | Diet high in sugar‐sweetened beverages | High body‐mass index | 1 | 1 - 1 |
| 56 | Peripheral artery disease | Diet high in sugar‐sweetened beverages | High body‐mass index |  | 1 - 1 |
| 57 | Ischemic heart disease | Diet high in sodium | High systolic blood pressure | 1 | 1 - 1 |
| 58 | Hemorrhagic stroke | Diet high in sodium | High systolic blood pressure | 1 | 1 - 1 |
| 59 | Ischemic stroke | Diet high in sodium | High systolic blood pressure | 1 | 1 - 1 |
| 60 | Atrial fibrillation and flutter | Diet high in sodium | High systolic blood pressure | 1 | 1 - 1 |
| 61 | Cardiomyopathy and myocarditis | Diet high in sodium | High systolic blood pressure | 1 | 1 - 1 |
| 62 | Hypertensive heart disease | Diet high in sodium | High systolic blood pressure | 1 | 1 - 1 |
| 63 | Other cardiovascular and circulatory diseases | Diet high in sodium | High systolic blood pressure | 1 | 1 - 1 |
| 64 | Peripheral artery disease | Diet high in sodium | High systolic blood pressure | 1 | 1 - 1 |
| 65 | Aortic aneurysm | Diet high in sodium | High systolic blood pressure | 1 | 1 - 1 |
| 66 | Rheumatic heart disease | Diet high in sodium | High systolic blood pressure | 1 | 1 - 1 |
| 67 | Ischemic heart disease | Diet high in processed meat | High body‐mass index | 0.03 | 0.02 - 0.04 |
| 68 | Ischemic heart disease | Diet high in processed meat | High fasting plasma glucose | 0.01 | 0.01 - 0.02 |
| 69 | Ischemic heart disease | Diet low in nuts and seeds | High body‐mass index | 0.01 | 0 - 0.02 |

Table 4 Citation of the epidemiological studies used to evaluate the causal relationship between dietary risk-outcome pairs

| **Risk-outcome pair** | **Risk factor** | **Cause of death or injury (outcome)** | **Citation** |
| --- | --- | --- | --- |
| 1 | Diet low in fiber | Ischemic heart disease | Threapleton DE, Greenwood DC, Evans CE, et al. Dietary fibre intake and risk of cardiovascular disease: systematic review and meta-analysis. BMJ (Clinical research ed) 2013; 347: f6879. |
| 2 | Diet low in fruits | Ischemic heart disease | Hu D, Huang J, Wang Y, Zhang D, Qu Y. Fruits and vegetables consumption and risk of stroke: a meta-analysis of prospective cohort studies. Stroke 2014; 45: 1613–9. |
| 3 | Diet low in fruits | Stroke (hemorrhagic and ischemic) | Hu D, Huang J, Wang Y, Zhang D, Qu Y. Fruits and vegetables consumption and risk of stroke: a meta-analysis of prospective cohort studies. Stroke 2014; 45: 1613–9. |
| 4 | Diet low in legumes | Ischemic heart disease | Afshin A, Micha R, Khatibzadeh S, Mozaffarian D. Consumption of nuts and legumes and risk of incident ischemic heart disease, stroke, and diabetes: a systematic review and meta-analysis. Am J Clin Nutr 2014; 100: 278–88. |
| 5 | Diet low in nuts and seeds | Ischemic heart disease | Afshin A, Micha R, Khatibzadeh S, Mozaffarian D. Consumption of nuts and legumes and risk of incident ischemic heart disease, stroke, and diabetes: a systematic review and meta-analysis. Am J Clin Nutr 2014; 100: 278–88. |
| 6 | Diet low in polyunsaturated fatty acids | Ischemic heart disease | Farvid MS, Ding M, Pan A, et al. Dietary linoleic acid and risk of coronary heart disease: a systematic review and meta-analysis of prospective cohort studies. Circulation 2014; 130: 1568–78.; Mozaffarian D, Micha R, Wallace S. Effects on coronary heart disease of increasing polyunsaturated fat in place of saturated fat: a systematic review and meta-analysis of randomized controlled trials. PLoS Med 2010; 7: e1000252. |
| 7 | Diet low in seafood omega-3 fatty acids | Ischemic heart disease | Chowdhury R, Stevens S, Gorman D, et al. Association between fish consumption, long chain omega 3 fatty acids, and risk of cerebrovascular disease: systematic review and meta-analysis. BMJ (Clinical research ed) 2012; 345: e6698. |
| 8 | Diet low in vegetables | Ischemic heart disease | Wang X, Ouyang Y, Liu J, et al. Fruit and vegetable consumption and mortality from all causes, cardiovascular disease, and cancer: systematic review and dose-response meta-analysis of prospective cohort studies. BMJ 2014; 349: g4490. |
| 9 | Diet low in vegetables | Stroke (hemorrhagic and ischemic) | Hu D, Huang J, Wang Y, Zhang D, Qu Y. Fruits and vegetables consumption and risk of stroke: a meta-analysis of prospective cohort studies. Stroke 2014; 45: 1613–9. |
| 10 | Diet low in whole grains | Ischemic heart disease | Aune D, Keum N, Giovannucci E, et al. Whole grain consumption and risk of cardiovascular disease, cancer, and all cause and cause specific mortality: systematic review and dose-response meta-analysis of prospective studies. BMJ 2016; 353: i2716. |
| 11 | Diet low in whole grains | Stroke (hemorrhagic and ischemic) | Aune D, Keum N, Giovannucci E, et al. Whole grain consumption and risk of cardiovascular disease, cancer, and all cause and cause specific mortality: systematic review and dose-response meta-analysis of prospective studies. BMJ 2016; 353: i2716. |
| 12 | Diet high in processed meat | Ischemic heart disease | Micha R, Wallace SK, Mozaffarian D. Red and processed meat consumption and risk of incident coronary heart disease, stroke, and diabetes mellitus: a systematic review and meta-analysis. Circulation 2010; 121: 2271–83. |
| 13 | Diet high in sodium | Other cardiovascular and circulatory diseases | Aburto NJ, Ziolkovska A, Hooper L, Elliott P, Cappuccio FP, Meerpohl JJ. Effect of lower sodium intake on health: systematic review and meta-analyses. BMJ 2013; 346: f1326. |
| 14 | Diet high in sodium | Endocarditis | Aburto NJ, Ziolkovska A, Hooper L, Elliott P, Cappuccio FP, Meerpohl JJ. Effect of lower sodium intake on health: systematic review and meta-analyses. BMJ 2013; 346: f1326. |
| 15 | Diet high in sodium | Peripheral artery disease | Aburto NJ, Ziolkovska A, Hooper L, Elliott P, Cappuccio FP, Meerpohl JJ. Effect of lower sodium intake on health: systematic review and meta-analyses. BMJ 2013; 346: f1326. |
| 16 | Diet high in sodium | Aortic aneurysm | Aburto NJ, Ziolkovska A, Hooper L, Elliott P, Cappuccio FP, Meerpohl JJ. Effect of lower sodium intake on health: systematic review and meta-analyses. BMJ 2013; 346: f1326. |
| 17 | Diet high in sodium | Atrial fibrillation and flutter | Aburto NJ, Ziolkovska A, Hooper L, Elliott P, Cappuccio FP, Meerpohl JJ. Effect of lower sodium intake on health: systematic review and meta-analyses. BMJ 2013; 346: f1326. |
| 18 | Diet high in sodium | Cardiomyopathy and myocarditis | Aburto NJ, Ziolkovska A, Hooper L, Elliott P, Cappuccio FP, Meerpohl JJ. Effect of lower sodium intake on health: systematic review and meta-analyses. BMJ 2013; 346: f1326. |
| 19 | Diet high in sodium | Hypertensive heart disease | Aburto NJ, Ziolkovska A, Hooper L, Elliott P, Cappuccio FP, Meerpohl JJ. Effect of lower sodium intake on health: systematic review and meta-analyses. BMJ 2013; 346: f1326. |
| 20 | Diet high in sodium | Stroke (hemorrhagic and ischemic) | Aburto NJ, Ziolkovska A, Hooper L, Elliott P, Cappuccio FP, Meerpohl JJ. Effect of lower sodium intake on health: systematic review and meta-analyses. BMJ 2013; 346: f1326. |
| 21 | Diet high in sodium | Ischemic heart disease | Aburto NJ, Ziolkovska A, Hooper L, Elliott P, Cappuccio FP, Meerpohl JJ. Effect of lower sodium intake on health: systematic review and meta-analyses. BMJ 2013; 346: f1326. |
| 22 | Diet high in sodium | Rheumatic heart disease | Aburto NJ, Ziolkovska A, Hooper L, Elliott P, Cappuccio FP, Meerpohl JJ. Effect of lower sodium intake on health: systematic review and meta-analyses. BMJ 2013; 346: f1326. |
| 23 | Diet high in sugar-sweetened beverages | Atrial fibrillation and flutter | Malik VS, Pan A, Willett WC, Hu FB. Sugar-sweetened beverages and weight gain in children and adults: a systematic review and meta-analysis. Am J Clin Nutr 2013; 98: 1084–102. |
| 24 | Diet high in sugar-sweetened beverages | Hypertensive heart disease | Malik VS, Pan A, Willett WC, Hu FB. Sugar-sweetened beverages and weight gain in children and adults: a systematic review and meta-analysis. Am J Clin Nutr 2013; 98: 1084–102. |
| 25 | Diet high in sugar-sweetened beverages | Stroke (hemorrhagic and ischemic) | Malik VS, Pan A, Willett WC, Hu FB. Sugar-sweetened beverages and weight gain in children and adults: a systematic review and meta-analysis. Am J Clin Nutr 2013; 98: 1084–102. |
| 26 | Diet high in sugar-sweetened beverages | Ischemic heart disease | Malik VS, Pan A, Willett WC, Hu FB. Sugar-sweetened beverages and weight gain in children and adults: a systematic review and meta-analysis. Am J Clin Nutr 2013; 98: 1084–102. |
| 27 | Diet high in trans fatty acids | Ischemic heart disease | Mozaffarian D, Clarke R. Quantitative effects on cardiovascular risk factors and coronary heart disease risk of replacing partially hydrogenated vegetable oils with other fats and oils. Eur J Clin Nutr. 2009; 63(Suppl 2): S22-33. |

Table 5 Death rate (age-standardized), number of deaths of cardiovascular diseases (CVDs) attributable to dietary risks from 1990 to 2016 (including 95% uncertainty interval)

|  | **Year** | **Age-standardized death rate (per 100,000)** | **Uncertainty Interval** | | **Number of deaths** | **Uncertainty Interval** | | **Absolute changes of number of deaths between 2010 and 2016*** | **Uncertainty Interval** | |
| --- | --- | --- | --- | --- | --- | --- | --- | --- | --- | --- |
| **Western** | 1990 | 155 | 135 | 177 | 824,027 | 713,008 | 943,836 |  |  |  |
| **Europe** | 1995 | 131 | 113 | 151 | 765,545 | 659,544 | 881,448 |  |  |  |
|  | 2000 | 109 | 93 | 125 | 695,252 | 594,815 | 801,533 |  |  |  |
|  | 2005 | 88 | 75 | 102 | 619,373 | 528,410 | 719,319 |  |  |  |
|  | 2010 | 71 | 61 | 83 | 568,663 | 480,091 | 664,385 |  |  |  |
|  | 2016 | 64 | 54 | 75 | 594,295 | 496,931 | 700,631 | 25,632 | 16,840 | 36,246 |
| Andorra | 1990 | 95 | 73 | 120 | 68 | 52 | 86 |  |  |  |
|  | 1995 | 82 | 64 | 105 | 77 | 59 | 98 |  |  |  |
|  | 2000 | 63 | 48 | 80 | 63 | 49 | 81 |  |  |  |
|  | 2005 | 55 | 42 | 69 | 71 | 54 | 90 |  |  |  |
|  | 2010 | 53 | 40 | 67 | 83 | 63 | 106 |  |  |  |
|  | 2016 | 54 | 40 | 70 | 94 | 71 | 121 | 12 | 9 | 15 |
| Austria | 1990 | 177 | 145 | 209 | 19,150 | 15,703 | 22,633 |  |  |  |
|  | 1995 | 164 | 137 | 191 | 18,981 | 15,731 | 22,202 |  |  |  |
|  | 2000 | 141 | 118 | 164 | 17,548 | 14,633 | 20,326 |  |  |  |
|  | 2005 | 111 | 92 | 129 | 15,218 | 12,598 | 17,750 |  |  |  |
|  | 2010 | 95 | 78 | 111 | 14,688 | 11,967 | 17,260 |  |  |  |
|  | 2016 | 83 | 68 | 99 | 15,186 | 12,158 | 18,203 | 497 | 192 | 943 |
| Belgium | 1990 | 149 | 128 | 171 | 20,952 | 17,960 | 24,113 |  |  |  |
|  | 1995 | 120 | 102 | 138 | 18,166 | 15,458 | 21,014 |  |  |  |
|  | 2000 | 103 | 88 | 121 | 16,938 | 14,319 | 19,770 |  |  |  |
|  | 2005 | 82 | 69 | 96 | 14,656 | 12,300 | 17,147 |  |  |  |
|  | 2010 | 67 | 56 | 78 | 13,368 | 11,180 | 15,751 |  |  |  |
|  | 2016 | 59 | 48 | 70 | 13,608 | 11,059 | 16,384 | 241 | -121 | 633 |
| Cyprus | 1990 | 191 | 157 | 228 | 1,266 | 1,039 | 1,512 |  |  |  |
|  | 1995 | 168 | 138 | 198 | 1,296 | 1,064 | 1,533 |  |  |  |
|  | 2000 | 147 | 121 | 173 | 1,250 | 1,030 | 1,478 |  |  |  |
|  | 2005 | 124 | 103 | 145 | 1,187 | 982 | 1,396 |  |  |  |
|  | 2010 | 96 | 79 | 113 | 1,107 | 911 | 1,308 |  |  |  |
|  | 2016 | 88 | 73 | 104 | 1,195 | 984 | 1,419 | 88 | 72 | 110 |
| Denmark | 1990 | 177 | 149 | 206 | 13,486 | 11,320 | 15,644 |  |  |  |
|  | 1995 | 156 | 130 | 181 | 12,418 | 10,375 | 14,449 |  |  |  |
|  | 2000 | 114 | 94 | 134 | 9,592 | 7,927 | 11,277 |  |  |  |
|  | 2005 | 87 | 72 | 103 | 7,705 | 6,334 | 9,185 |  |  |  |
|  | 2010 | 66 | 54 | 78 | 6,239 | 5,109 | 7,410 |  |  |  |
|  | 2016 | 55 | 44 | 67 | 5,854 | 4,716 | 7,190 | -385 | -394 | -220 |
| Finland | 1990 | 226 | 199 | 254 | 13,985 | 12,279 | 15,700 |  |  |  |
|  | 1995 | 186 | 163 | 209 | 12,639 | 11,070 | 14,230 |  |  |  |
|  | 2000 | 151 | 132 | 172 | 11,367 | 9,875 | 12,887 |  |  |  |
|  | 2005 | 125 | 107 | 143 | 10,507 | 9,012 | 12,038 |  |  |  |
|  | 2010 | 107 | 91 | 123 | 10,426 | 8,854 | 12,020 |  |  |  |
|  | 2016 | 87 | 72 | 102 | 10,029 | 8,335 | 11,766 | -397 | -519 | -255 |
| France | 1990 | 96 | 81 | 112 | 76,876 | 64,555 | 89,704 |  |  |  |
|  | 1995 | 84 | 71 | 98 | 74,362 | 62,605 | 87,287 |  |  |  |
|  | 2000 | 74 | 63 | 86 | 72,282 | 60,936 | 84,664 |  |  |  |
|  | 2005 | 61 | 52 | 72 | 66,201 | 55,481 | 77,889 |  |  |  |
|  | 2010 | 51 | 43 | 60 | 63,224 | 52,837 | 74,989 |  |  |  |
|  | 2016 | 46 | 38 | 54 | 66,801 | 55,087 | 79,825 | 3577 | 2250 | 4837 |
| Germany | 1990 | 208 | 179 | 237 | 241,104 | 207,231 | 275,194 |  |  |  |
|  | 1995 | 174 | 150 | 199 | 215,255 | 184,848 | 246,574 |  |  |  |
|  | 2000 | 143 | 122 | 165 | 190,847 | 162,745 | 220,486 |  |  |  |
|  | 2005 | 117 | 99 | 135 | 170,870 | 145,025 | 198,151 |  |  |  |
|  | 2010 | 97 | 83 | 114 | 159,150 | 134,048 | 185,849 |  |  |  |
|  | 2016 | 87 | 73 | 103 | 164,639 | 136,783 | 194,795 | 5,489 | 2,735 | 8,946 |
| Greece | 1990 | 153 | 124 | 183 | 20,693 | 16,742 | 24,847 |  |  |  |
|  | 1995 | 140 | 113 | 167 | 21,105 | 17,020 | 25,378 |  |  |  |
|  | 2000 | 129 | 104 | 154 | 21,595 | 17,415 | 25,891 |  |  |  |
|  | 2005 | 115 | 93 | 138 | 21,467 | 17,280 | 25,778 |  |  |  |
|  | 2010 | 106 | 87 | 127 | 22,505 | 18,193 | 27,115 |  |  |  |
|  | 2016 | 100 | 81 | 119 | 25,785 | 20,764 | 31,139 | 3,280 | 2,571 | 4,024 |
| Iceland | 1990 | 164 | 143 | 184 | 440 | 383 | 495 |  |  |  |
|  | 1995 | 142 | 123 | 161 | 422 | 364 | 480 |  |  |  |
|  | 2000 | 117 | 101 | 133 | 379 | 328 | 432 |  |  |  |
|  | 2005 | 91 | 78 | 104 | 338 | 289 | 389 |  |  |  |
|  | 2010 | 76 | 64 | 88 | 326 | 275 | 377 |  |  |  |
|  | 2016 | 69 | 58 | 81 | 353 | 297 | 413 | 27 | 22 | 36 |
| Ireland | 1990 | 222 | 190 | 253 | 8,098 | 6,939 | 9,244 |  |  |  |
|  | 1995 | 193 | 165 | 219 | 7,505 | 6,435 | 8,555 |  |  |  |
|  | 2000 | 159 | 136 | 182 | 6,615 | 5,649 | 7,605 |  |  |  |
|  | 2005 | 114 | 98 | 131 | 5,296 | 4,516 | 6,074 |  |  |  |
|  | 2010 | 88 | 73 | 101 | 4,706 | 3,922 | 5,443 |  |  |  |
|  | 2016 | 79 | 64 | 95 | 4,984 | 4,033 | 5,974 | 279 | 111 | 531 |
| Israel | 1990 | 173 | 137 | 211 | 6,465 | 5,089 | 7,907 |  |  |  |
|  | 1995 | 137 | 110 | 168 | 6,664 | 5,308 | 8,125 |  |  |  |
|  | 2000 | 87 | 66 | 107 | 5,086 | 3,892 | 6,293 |  |  |  |
|  | 2005 | 66 | 50 | 82 | 4,522 | 3,396 | 5,630 |  |  |  |
|  | 2010 | 48 | 36 | 60 | 4,107 | 3,096 | 5,128 |  |  |  |
|  | 2016 | 43 | 32 | 57 | 4,486 | 3,279 | 5,815 | 379 | 183 | 687 |
| Italy | 1990 | 125 | 103 | 151 | 99,131 | 80,969 | 119,356 |  |  |  |
|  | 1995 | 108 | 88 | 129 | 97,285 | 79,379 | 117,329 |  |  |  |
|  | 2000 | 92 | 75 | 110 | 94,600 | 76,768 | 113,066 |  |  |  |
|  | 2005 | 77 | 63 | 92 | 89,411 | 72,444 | 107,491 |  |  |  |
|  | 2010 | 65 | 53 | 79 | 87,349 | 70,177 | 105,710 |  |  |  |
|  | 2016 | 61 | 49 | 74 | 96,977 | 76,694 | 118,592 | 9,628 | 6,516 | 12,882 |
| Luxembourg | 1990 | 170 | 142 | 199 | 821 | 683 | 959 |  |  |  |
|  | 1995 | 136 | 112 | 159 | 709 | 585 | 834 |  |  |  |
|  | 2000 | 111 | 92 | 130 | 634 | 524 | 744 |  |  |  |
|  | 2005 | 91 | 75 | 108 | 581 | 478 | 685 |  |  |  |
|  | 2010 | 71 | 57 | 84 | 521 | 422 | 619 |  |  |  |
|  | 2016 | 62 | 49 | 76 | 568 | 450 | 694 | 48 | 28 | 75 |
| Malta | 1990 | 217 | 181 | 254 | 728 | 607 | 852 |  |  |  |
|  | 1995 | 168 | 139 | 198 | 643 | 532 | 760 |  |  |  |
|  | 2000 | 156 | 128 | 185 | 688 | 565 | 818 |  |  |  |
|  | 2005 | 121 | 100 | 143 | 652 | 539 | 768 |  |  |  |
|  | 2010 | 101 | 82 | 120 | 630 | 513 | 748 |  |  |  |
|  | 2016 | 87 | 68 | 108 | 644 | 506 | 805 | 14 | -7 | 57 |
| Netherlands | 1990 | 145 | 124 | 166 | 26,445 | 22,550 | 30,234 |  |  |  |
|  | 1995 | 127 | 109 | 146 | 25,264 | 21,664 | 28,966 |  |  |  |
|  | 2000 | 109 | 93 | 126 | 23,473 | 20,021 | 27,157 |  |  |  |
|  | 2005 | 81 | 69 | 95 | 19,093 | 16,142 | 22,372 |  |  |  |
|  | 2010 | 61 | 51 | 72 | 16,236 | 13,686 | 19,168 |  |  |  |
|  | 2016 | 52 | 43 | 63 | 16,301 | 13,435 | 19,917 | 65 | -251 | 748 |
| Norway | 1990 | 170 | 145 | 196 | 10,827 | 9,125 | 12,561 |  |  |  |
|  | 1995 | 146 | 123 | 169 | 9,884 | 8,277 | 11,505 |  |  |  |
|  | 2000 | 120 | 100 | 139 | 8,726 | 7,294 | 10,193 |  |  |  |
|  | 2005 | 89 | 74 | 104 | 6,982 | 5,782 | 8,207 |  |  |  |
|  | 2010 | 72 | 60 | 85 | 6,164 | 5,112 | 7,291 |  |  |  |
|  | 2016 | 61 | 49 | 74 | 5,818 | 4,657 | 7,055 | -346 | -456 | -236 |
| Portugal | 1990 | 165 | 139 | 194 | 20,388 | 17,176 | 24,088 |  |  |  |
|  | 1995 | 140 | 117 | 166 | 19,410 | 16,256 | 23,061 |  |  |  |
|  | 2000 | 117 | 98 | 138 | 18,013 | 15,056 | 21,368 |  |  |  |
|  | 2005 | 92 | 77 | 109 | 16,061 | 13,359 | 19,106 |  |  |  |
|  | 2010 | 71 | 58 | 85 | 14,122 | 11,532 | 16,952 |  |  |  |
|  | 2016 | 63 | 51 | 76 | 14,499 | 11,574 | 17,620 | 377 | 43 | 668 |
| Spain | 1990 | 105 | 87 | 127 | 51,313 | 42,146 | 61,824 |  |  |  |
|  | 1995 | 87 | 72 | 105 | 48,879 | 40,081 | 58,960 |  |  |  |
|  | 2000 | 74 | 61 | 89 | 47,392 | 38,739 | 57,454 |  |  |  |
|  | 2005 | 63 | 51 | 76 | 46,596 | 37,780 | 56,377 |  |  |  |
|  | 2010 | 49 | 40 | 60 | 42,038 | 33,827 | 51,563 |  |  |  |
|  | 2016 | 43 | 35 | 53 | 44,617 | 35,661 | 54,825 | 2,579 | 1,833 | 3,262 |
| Sweden | 1990 | 178 | 151 | 202 | 25,594 | 21,643 | 29,202 |  |  |  |
|  | 1995 | 148 | 126 | 170 | 23,227 | 19,648 | 26,725 |  |  |  |
|  | 2000 | 127 | 107 | 146 | 21,381 | 17,924 | 24,755 |  |  |  |
|  | 2005 | 105 | 88 | 122 | 19,018 | 15,832 | 22,083 |  |  |  |
|  | 2010 | 86 | 71 | 100 | 16,681 | 13,771 | 19,562 |  |  |  |
|  | 2016 | 77 | 61 | 93 | 16,164 | 12,849 | 19,567 | -517 | -922 | 5 |
| Switzerland | 1990 | 136 | 116 | 158 | 13,106 | 11,114 | 15,288 |  |  |  |
|  | 1995 | 123 | 104 | 142 | 13,132 | 11,088 | 15,262 |  |  |  |
|  | 2000 | 103 | 87 | 119 | 12,122 | 10,209 | 14,064 |  |  |  |
|  | 2005 | 84 | 70 | 97 | 10,934 | 9,073 | 12,764 |  |  |  |
|  | 2010 | 69 | 57 | 81 | 10,150 | 8,316 | 12,048 |  |  |  |
|  | 2016 | 60 | 44 | 76 | 10,349 | 7,580 | 13,259 | 199 | -735 | 1,211 |
| United Kingdom | 1990 | 184 | 164 | 202 | 153,090 | 136,493 | 168,966 |  |  |  |
|  | 1995 | 157 | 140 | 173 | 138,224 | 122,978 | 152,824 |  |  |  |
|  | 2000 | 123 | 110 | 137 | 114,659 | 101,588 | 127,258 |  |  |  |
|  | 2005 | 93 | 82 | 103 | 92,009 | 81,220 | 102,597 |  |  |  |
|  | 2010 | 69 | 62 | 78 | 74,844 | 66,248 | 84,177 |  |  |  |
|  | 2016 | 62 | 55 | 70 | 75,343 | 66,069 | 85,339 | 499 | -179 | 1,162 |
| **Central** | 1990 | 373 | 330 | 413 | 451,098 | 400,652 | 498,559 |  |  |  |
| **Europe** | 1995 | 358 | 316 | 398 | 457,933 | 404,855 | 508,781 |  |  |  |
|  | 2000 | 290 | 252 | 327 | 398,029 | 346,696 | 449,213 |  |  |  |
|  | 2005 | 243 | 210 | 278 | 364,225 | 313,671 | 415,901 |  |  |  |
|  | 2010 | 207 | 178 | 237 | 346,403 | 297,683 | 398,330 |  |  |  |
|  | 2016 | 177 | 150 | 203 | 341,032 | 288,961 | 393,849 | -5,372 | -8,722 | -4,481 |
| Albania | 1990 | 240 | 207 | 274 | 4,181 | 3,607 | 4,718 |  |  |  |
|  | 1995 | 228 | 194 | 261 | 4,322 | 3,706 | 4,913 |  |  |  |
|  | 2000 | 225 | 190 | 264 | 4,717 | 4,000 | 5,504 |  |  |  |
|  | 2005 | 231 | 192 | 272 | 5,662 | 4,703 | 6,616 |  |  |  |
|  | 2010 | 204 | 165 | 244 | 5,912 | 4,798 | 7,080 |  |  |  |
|  | 2016 | 174 | 137 | 212 | 6,085 | 4,813 | 7,475 | 172 | 15 | 395 |
| Bosnia and Herzegovina | 1990 | 359 | 302 | 416 | 10,857 | 9,170 | 12,621 |  |  |  |
|  | 1995 | 335 | 281 | 391 | 10,612 | 8,921 | 12,399 |  |  |  |
|  | 2000 | 239 | 197 | 286 | 8,783 | 7,242 | 10,439 |  |  |  |
|  | 2005 | 195 | 159 | 233 | 8,585 | 6,985 | 10,331 |  |  |  |
|  | 2010 | 166 | 135 | 199 | 8,428 | 6,838 | 10,091 |  |  |  |
|  | 2016 | 153 | 121 | 188 | 9,081 | 7,224 | 11,195 | 653 | 386 | 1,104 |
| Bulgaria | 1990 | 432 | 372 | 489 | 43,251 | 37,209 | 48,821 |  |  |  |
|  | 1995 | 446 | 385 | 505 | 46,834 | 40,303 | 52,806 |  |  |  |
|  | 2000 | 389 | 332 | 448 | 42,120 | 35,809 | 48,665 |  |  |  |
|  | 2005 | 339 | 287 | 394 | 38,975 | 32,883 | 45,492 |  |  |  |
|  | 2010 | 291 | 246 | 342 | 35,574 | 29,772 | 41,929 |  |  |  |
|  | 2016 | 260 | 211 | 319 | 35,298 | 28,440 | 43,692 | -276 | -1332 | 1,763 |
| Croatia | 1990 | 332 | 283 | 376 | 17,757 | 15,256 | 20,073 |  |  |  |
|  | 1995 | 306 | 261 | 348 | 17,273 | 14,782 | 19,610 |  |  |  |
|  | 2000 | 253 | 218 | 290 | 15,136 | 12,992 | 17,347 |  |  |  |
|  | 2005 | 221 | 187 | 257 | 14,255 | 12,021 | 16,648 |  |  |  |
|  | 2010 | 190 | 161 | 220 | 13,910 | 11,701 | 16,171 |  |  |  |
|  | 2016 | 170 | 140 | 203 | 14,208 | 11,607 | 17,005 | 298 | -94 | 834 |
| Czech Republic | 1990 | 415 | 370 | 464 | 47,908 | 42,584 | 53,337 |  |  |  |
|  | 1995 | 352 | 311 | 393 | 42,029 | 37,092 | 46,852 |  |  |  |
|  | 2000 | 271 | 236 | 306 | 34,280 | 29,857 | 38,685 |  |  |  |
|  | 2005 | 224 | 195 | 253 | 30,528 | 26,568 | 34,474 |  |  |  |
|  | 2010 | 190 | 166 | 214 | 29,227 | 25,435 | 33,068 |  |  |  |
|  | 2016 | 159 | 136 | 181 | 28,574 | 24,428 | 32,717 | -653 | -1,007 | -351 |
| Hungary | 1990 | 388 | 338 | 436 | 48,335 | 42,037 | 54,361 |  |  |  |
|  | 1995 | 368 | 323 | 410 | 47,638 | 41,903 | 53,332 |  |  |  |
|  | 2000 | 308 | 266 | 347 | 41,818 | 36,124 | 47,150 |  |  |  |
|  | 2005 | 248 | 211 | 283 | 35,978 | 30,567 | 41,213 |  |  |  |
|  | 2010 | 212 | 179 | 245 | 33,375 | 28,224 | 38,641 |  |  |  |
|  | 2016 | 192 | 160 | 228 | 33,539 | 27,930 | 39,812 | 163 | -294 | 1,171 |
| Macedonia | 1990 | 344 | 281 | 411 | 4,919 | 4,100 | 5,803 |  |  |  |
|  | 1995 | 341 | 278 | 413 | 4,964 | 4,115 | 5,889 |  |  |  |
|  | 2000 | 290 | 229 | 357 | 4,907 | 3,914 | 5,975 |  |  |  |
|  | 2005 | 264 | 210 | 328 | 4,979 | 4,034 | 6,078 |  |  |  |
|  | 2010 | 225 | 177 | 284 | 4,901 | 3,891 | 6,100 |  |  |  |
|  | 2016 | 197 | 152 | 249 | 4,948 | 3,852 | 6,211 | 47 | -38 | 111 |
| Montenegro | 1990 | 260 | 218 | 306 | 1,426 | 1,196 | 1,681 |  |  |  |
|  | 1995 | 288 | 241 | 337 | 1,723 | 1,445 | 2,006 |  |  |  |
|  | 2000 | 268 | 221 | 317 | 1,742 | 1,437 | 2,050 |  |  |  |
|  | 2005 | 241 | 200 | 282 | 1,689 | 1,408 | 1,971 |  |  |  |
|  | 2010 | 202 | 165 | 242 | 1,503 | 1,221 | 1,802 |  |  |  |
|  | 2016 | 186 | 150 | 226 | 1,582 | 1,276 | 1,922 | 79 | 55 | 120 |
| Poland | 1990 | 360 | 314 | 403 | 129,382 | 112,973 | 144,900 |  |  |  |
|  | 1995 | 329 | 285 | 370 | 124,625 | 108,116 | 140,195 |  |  |  |
|  | 2000 | 256 | 221 | 291 | 105,140 | 90,850 | 119,486 |  |  |  |
|  | 2005 | 207 | 179 | 237 | 95,630 | 82,451 | 109,256 |  |  |  |
|  | 2010 | 179 | 154 | 206 | 94,554 | 81,122 | 108,628 |  |  |  |
|  | 2016 | 150 | 125 | 175 | 94,291 | 78,937 | 110,004 | -263 | -2,185 | 1,376 |
| Romania | 1990 | 388 | 332 | 442 | 89,568 | 76,762 | 102,192 |  |  |  |
|  | 1995 | 412 | 352 | 468 | 100,156 | 86,195 | 113,932 |  |  |  |
|  | 2000 | 321 | 269 | 375 | 84,555 | 70,563 | 98,583 |  |  |  |
|  | 2005 | 277 | 230 | 328 | 77,102 | 63,573 | 91,593 |  |  |  |
|  | 2010 | 236 | 195 | 281 | 72,735 | 59,714 | 86,628 |  |  |  |
|  | 2016 | 206 | 167 | 250 | 70,166 | 56,558 | 85,548 | -2,568 | -3,156 | -1,080 |
| Serbia | 1990 | 308 | 254 | 363 | 27,287 | 22,760 | 32,089 |  |  |  |
|  | 1995 | 339 | 282 | 396 | 33,299 | 27,882 | 38,557 |  |  |  |
|  | 2000 | 300 | 248 | 359 | 32,720 | 27,304 | 38,924 |  |  |  |
|  | 2005 | 257 | 209 | 311 | 29,828 | 24,431 | 35,962 |  |  |  |
|  | 2010 | 206 | 166 | 255 | 26,625 | 21,441 | 32,644 |  |  |  |
|  | 2016 | 168 | 134 | 209 | 23,971 | 18,949 | 29,963 | -2,654 | -2,491 | -2,681 |
| Slovakia | 1990 | 439 | 392 | 484 | 21,114 | 18,823 | 23,294 |  |  |  |
|  | 1995 | 386 | 341 | 429 | 19,599 | 17,303 | 21,693 |  |  |  |
|  | 2000 | 336 | 294 | 375 | 18,142 | 15,853 | 20,183 |  |  |  |
|  | 2005 | 298 | 259 | 335 | 17,533 | 15,264 | 19,675 |  |  |  |
|  | 2010 | 250 | 217 | 282 | 16,292 | 14,169 | 18,426 |  |  |  |
|  | 2016 | 206 | 171 | 240 | 15,643 | 13,002 | 18,262 | -649 | -1,167 | -164 |
| Slovenia | 1990 | 248 | 206 | 292 | 5,114 | 4,242 | 6,019 |  |  |  |
|  | 1995 | 217 | 183 | 255 | 4,858 | 4,072 | 5,711 |  |  |  |
|  | 2000 | 157 | 129 | 186 | 3,968 | 3,273 | 4,714 |  |  |  |
|  | 2005 | 125 | 100 | 150 | 3,482 | 2,788 | 4,184 |  |  |  |
|  | 2010 | 100 | 81 | 120 | 3,367 | 2,706 | 4,061 |  |  |  |
|  | 2016 | 86 | 69 | 106 | 3,646 | 2,889 | 4,515 | 280 | 183 | 454 |
| **Eastern** | 1990 | 357 | 308 | 407 | 812,867 | 703,380 | 928,019 |  |  |  |
| **Europe** | 1995 | 457 | 395 | 520 | 1,093,710 | 944,391 | 1,243,483 |  |  |  |
|  | 2000 | 438 | 377 | 498 | 1,088,619 | 938,620 | 1,237,444 |  |  |  |
|  | 2005 | 434 | 374 | 492 | 1,117,424 | 962,395 | 1,267,238 |  |  |  |
|  | 2010 | 351 | 300 | 402 | 957,273 | 817,596 | 1,102,742 |  |  |  |
|  | 2016 | 304 | 240 | 376 | 936,950 | 738,494 | 1,162,390 | -20,323 | -79,102 | 59,647 |
| Belarus | 1990 | 349 | 306 | 393 | 38,573 | 33,822 | 43,317 |  |  |  |
|  | 1995 | 435 | 379 | 487 | 49,233 | 42,909 | 55,089 |  |  |  |
|  | 2000 | 445 | 390 | 499 | 51,786 | 45,413 | 58,054 |  |  |  |
|  | 2005 | 443 | 386 | 498 | 53,647 | 46,761 | 60,380 |  |  |  |
|  | 2010 | 397 | 343 | 450 | 51,170 | 44,152 | 58,086 |  |  |  |
|  | 2016 | 313 | 254 | 374 | 44,568 | 36,132 | 53,629 | -6,602 | -8,020 | -4,457 |
| Estonia | 1990 | 391 | 340 | 440 | 6,955 | 6,052 | 7,822 |  |  |  |
|  | 1995 | 419 | 367 | 469 | 7,511 | 6,580 | 8,401 |  |  |  |
|  | 2000 | 332 | 288 | 376 | 6,227 | 5,397 | 7,052 |  |  |  |
|  | 2005 | 272 | 232 | 311 | 5,399 | 4,596 | 6,188 |  |  |  |
|  | 2010 | 197 | 159 | 235 | 4,337 | 3,489 | 5,207 |  |  |  |
|  | 2016 | 170 | 133 | 209 | 4,491 | 3,484 | 5,538 | 155 | -5 | 331 |
| Latvia | 1990 | 395 | 345 | 441 | 12,342 | 10,792 | 13,802 |  |  |  |
|  | 1995 | 452 | 399 | 503 | 13,934 | 12,287 | 15,525 |  |  |  |
|  | 2000 | 350 | 308 | 393 | 10,989 | 9,652 | 12,366 |  |  |  |
|  | 2005 | 339 | 295 | 378 | 10,938 | 9,504 | 12,271 |  |  |  |
|  | 2010 | 267 | 229 | 302 | 9,202 | 7,896 | 10,446 |  |  |  |
|  | 2016 | 232 | 192 | 270 | 9,004 | 7,428 | 10,536 | -198 | -469 | 90 |
| Lithuania | 1990 | 339 | 299 | 378 | 13,891 | 12,261 | 15,475 |  |  |  |
|  | 1995 | 380 | 334 | 422 | 15,795 | 13,905 | 17,568 |  |  |  |
|  | 2000 | 289 | 251 | 326 | 12,400 | 10,783 | 14,003 |  |  |  |
|  | 2005 | 288 | 248 | 324 | 13,417 | 11,533 | 15,116 |  |  |  |
|  | 2010 | 251 | 216 | 285 | 12,619 | 10,829 | 14,357 |  |  |  |
|  | 2016 | 214 | 182 | 247 | 12,187 | 10,339 | 14,157 | -433 | -490 | -200 |
| Moldova | 1990 | 438 | 377 | 502 | 14,446 | 12,382 | 16,577 |  |  |  |
|  | 1995 | 553 | 474 | 628 | 18,955 | 16,280 | 21,530 |  |  |  |
|  | 2000 | 459 | 394 | 524 | 16,289 | 14,045 | 18,625 |  |  |  |
|  | 2005 | 446 | 383 | 511 | 16,833 | 14,480 | 19,259 |  |  |  |
|  | 2010 | 397 | 340 | 458 | 16,314 | 13,980 | 18,685 |  |  |  |
|  | 2016 | 328 | 276 | 381 | 14,746 | 12,420 | 17,069 | -1,568 | -1,560 | -1,616 |
| Russia | 1990 | 349 | 299 | 403 | 507,708 | 433,756 | 585,259 |  |  |  |
|  | 1995 | 450 | 385 | 520 | 697,813 | 596,306 | 804,068 |  |  |  |
|  | 2000 | 435 | 372 | 501 | 708,378 | 605,090 | 815,156 |  |  |  |
|  | 2005 | 425 | 363 | 489 | 719,677 | 614,549 | 830,901 |  |  |  |
|  | 2010 | 336 | 284 | 391 | 606,717 | 513,122 | 705,314 |  |  |  |
|  | 2016 | 291 | 212 | 385 | 598,759 | 432,818 | 794,563 | -7,958 | -80,304 | 89,249 |
| Ukraine | 1990 | 369 | 315 | 420 | 218,952 | 187,284 | 249,152 |  |  |  |
|  | 1995 | 480 | 413 | 545 | 290,469 | 249,859 | 329,793 |  |  |  |
|  | 2000 | 461 | 394 | 521 | 282,549 | 241,444 | 319,273 |  |  |  |
|  | 2005 | 473 | 404 | 538 | 297,513 | 253,682 | 339,064 |  |  |  |
|  | 2010 | 394 | 332 | 453 | 256,914 | 216,377 | 295,804 |  |  |  |
|  | 2016 | 349 | 271 | 450 | 253,196 | 196,460 | 328,161 | -3,718 | -19,917 | 32,357 |
| **Central Asia** | 1990 | 400 | 352 | 448 | 198,596 | 171,141 | 226,688 |  |  |  |
| **+ Turkey** | 1995 | 455 | 400 | 510 | 242,158 | 208,570 | 277,055 |  |  |  |
|  | 2000 | 415 | 360 | 469 | 236,424 | 201,864 | 271,660 |  |  |  |
|  | 2005 | 393 | 337 | 450 | 238,716 | 202,155 | 276,904 |  |  |  |
|  | 2010 | 344 | 293 | 397 | 223,109 | 187,175 | 261,258 |  |  |  |
|  | 2016 | 289 | 240 | 343 | 227,360 | 183,808 | 275,990 | 4,251 | -3,367 | 14,731 |
| Armenia | 1990 | 367 | 322 | 406 | 8,122 | 7,166 | 8,983 |  |  |  |
|  | 1995 | 402 | 353 | 446 | 9,637 | 8,502 | 10,638 |  |  |  |
|  | 2000 | 325 | 282 | 368 | 8,741 | 7,568 | 9,866 |  |  |  |
|  | 2005 | 289 | 248 | 328 | 8,441 | 7,222 | 9,655 |  |  |  |
|  | 2010 | 255 | 217 | 294 | 7,933 | 6,743 | 9,194 |  |  |  |
|  | 2016 | 204 | 171 | 238 | 7,686 | 6,392 | 8,963 | -246 | -351 | -231 |
| Azerbaijan | 1990 | 475 | 423 | 528 | 16,512 | 14,739 | 18,249 |  |  |  |
|  | 1995 | 520 | 463 | 578 | 18,430 | 16,441 | 20,419 |  |  |  |
|  | 2000 | 490 | 428 | 549 | 19,428 | 16,965 | 21,758 |  |  |  |
|  | 2005 | 445 | 377 | 509 | 20,835 | 17,739 | 23,713 |  |  |  |
|  | 2010 | 420 | 354 | 485 | 22,435 | 19,020 | 25,906 |  |  |  |
|  | 2016 | 319 | 254 | 391 | 22,418 | 17,743 | 27,380 | -18 | -1,277 | 1,474 |
| Georgia | 1990 | 459 | 401 | 518 | 23,583 | 20,616 | 26,565 |  |  |  |
|  | 1995 | 444 | 390 | 503 | 22,446 | 19,689 | 25,396 |  |  |  |
|  | 2000 | 389 | 336 | 441 | 20,594 | 17,801 | 23,380 |  |  |  |
|  | 2005 | 357 | 306 | 404 | 19,901 | 17,047 | 22,562 |  |  |  |
|  | 2010 | 316 | 271 | 365 | 18,176 | 15,515 | 21,001 |  |  |  |
|  | 2016 | 278 | 227 | 333 | 16,486 | 13,290 | 19,833 | -1,691 | -2,225 | -1,168 |
| Kazakhstan | 1990 | 407 | 360 | 458 | 42,389 | 37,468 | 47,651 |  |  |  |
|  | 1995 | 534 | 471 | 600 | 58,198 | 51,361 | 65,481 |  |  |  |
|  | 2000 | 498 | 433 | 565 | 53,530 | 46,524 | 60,449 |  |  |  |
|  | 2005 | 482 | 409 | 555 | 53,628 | 45,604 | 61,674 |  |  |  |
|  | 2010 | 386 | 328 | 448 | 43,592 | 37,296 | 50,397 |  |  |  |
|  | 2016 | 306 | 248 | 371 | 39,632 | 31,982 | 48,298 | -3,961 | -5,314 | -2,099 |
| Kyrgyzstan | 1990 | 402 | 358 | 443 | 9,420 | 8,407 | 10,375 |  |  |  |
|  | 1995 | 506 | 449 | 562 | 11,745 | 10,444 | 13,005 |  |  |  |
|  | 2000 | 423 | 373 | 475 | 10,635 | 9,402 | 11,897 |  |  |  |
|  | 2005 | 447 | 391 | 507 | 11,681 | 10,234 | 13,157 |  |  |  |
|  | 2010 | 419 | 366 | 472 | 11,248 | 9,819 | 12,585 |  |  |  |
|  | 2016 | 350 | 299 | 402 | 10,627 | 9,137 | 12,225 | -621 | -682 | -360 |
| Tajikistan | 1990 | 402 | 349 | 453 | 8,662 | 7,528 | 9,792 |  |  |  |
|  | 1995 | 445 | 387 | 501 | 9,525 | 8,323 | 10,708 |  |  |  |
|  | 2000 | 448 | 387 | 508 | 9,546 | 8,229 | 10,822 |  |  |  |
|  | 2005 | 441 | 377 | 506 | 10,405 | 8,882 | 11,921 |  |  |  |
|  | 2010 | 374 | 320 | 435 | 10,090 | 8,620 | 11,714 |  |  |  |
|  | 2016 | 310 | 260 | 371 | 9,932 | 8,369 | 11,930 | -158 | -251 | 216 |
| Turkey | 1990 | 129 | 102 | 159 | 41,933 | 33,422 | 51,269 |  |  |  |
|  | 1995 | 135 | 107 | 167 | 50,340 | 39,937 | 61,823 |  |  |  |
|  | 2000 | 111 | 88 | 137 | 47,041 | 37,557 | 57,454 |  |  |  |
|  | 2005 | 86 | 69 | 106 | 39,649 | 31,651 | 48,421 |  |  |  |
|  | 2010 | 72 | 57 | 91 | 37,914 | 29,785 | 47,489 |  |  |  |
|  | 2016 | 67 | 50 | 87 | 44,298 | 33,452 | 57,146 | 6,384 | 3,667 | 9,656 |
| Turkmenistan | 1990 | 518 | 466 | 561 | 7,752 | 6,999 | 8,377 |  |  |  |
|  | 1995 | 576 | 520 | 630 | 9,810 | 8,872 | 10,662 |  |  |  |
|  | 2000 | 535 | 477 | 593 | 10,490 | 9,401 | 11,560 |  |  |  |
|  | 2005 | 481 | 423 | 538 | 10,521 | 9,301 | 11,732 |  |  |  |
|  | 2010 | 417 | 363 | 471 | 10,060 | 8,824 | 11,287 |  |  |  |
|  | 2016 | 376 | 326 | 427 | 10,483 | 9,186 | 11,876 | 423 | 363 | 589 |
| Uzbekistan | 1990 | 444 | 383 | 503 | 40,222 | 34,796 | 45,428 |  |  |  |
|  | 1995 | 529 | 456 | 603 | 52,027 | 45,000 | 58,922 |  |  |  |
|  | 2000 | 512 | 438 | 588 | 56,420 | 48,416 | 64,474 |  |  |  |
|  | 2005 | 511 | 435 | 596 | 63,655 | 54,475 | 74,070 |  |  |  |
|  | 2010 | 436 | 362 | 509 | 61,660 | 51,553 | 71,685 |  |  |  |
|  | 2016 | 394 | 323 | 472 | 65,799 | 54,256 | 78,339 | 4,138 | 2,703 | 6,653 |
| **EU-28** | 1990 | 197 | 173 | 221 | 1,228,737 | 1,055,026 | 1,404,167 |  |  |  |
|  | 1995 | 174 | 152 | 197 | 1,175,619 | 1,008,267 | 1,346,307 |  |  |  |
|  | 2000 | 142 | 123 | 162 | 1,043,651 | 886,866 | 1,205,238 |  |  |  |
|  | 2005 | 116 | 100 | 134 | 939,764 | 793,070 | 1,092,995 |  |  |  |
|  | 2010 | 96 | 82 | 111 | 873,025 | 732,255 | 1,023,207 |  |  |  |
|  | 2016 | 85 | 72 | 98 | 894,241 | 736,198 | 1,065,840 | 21,216 | 3,943 | 42,633 |
| **WHO-Europe** | 1990 | 321 | 281 | 361 | 2,286,588 | 1,960,007 | 2,618,408 |  |  |  |
| **Region** | 1995 | 350 | 306 | 395 | 2,559,346 | 2,193,356 | 2,933,077 |  |  |  |
|  | 2000 | 313 | 271 | 355 | 2,418,325 | 2,060,389 | 2,781,961 |  |  |  |
|  | 2005 | 290 | 249 | 330 | 2,339,737 | 1,984,851 | 2,707,541 |  |  |  |
|  | 2010 | 243 | 208 | 280 | 2,095,448 | 1,762,111 | 2,445,021 |  |  |  |
|  | 2016 | 208 | 171 | 249 | 2,099,637 | 1,708,193 | 2,532,859 | 4,189 | -53,918 | 87,838 |

* Red numbers indicate an increase in absolute death numbers between 2010 and 2016.


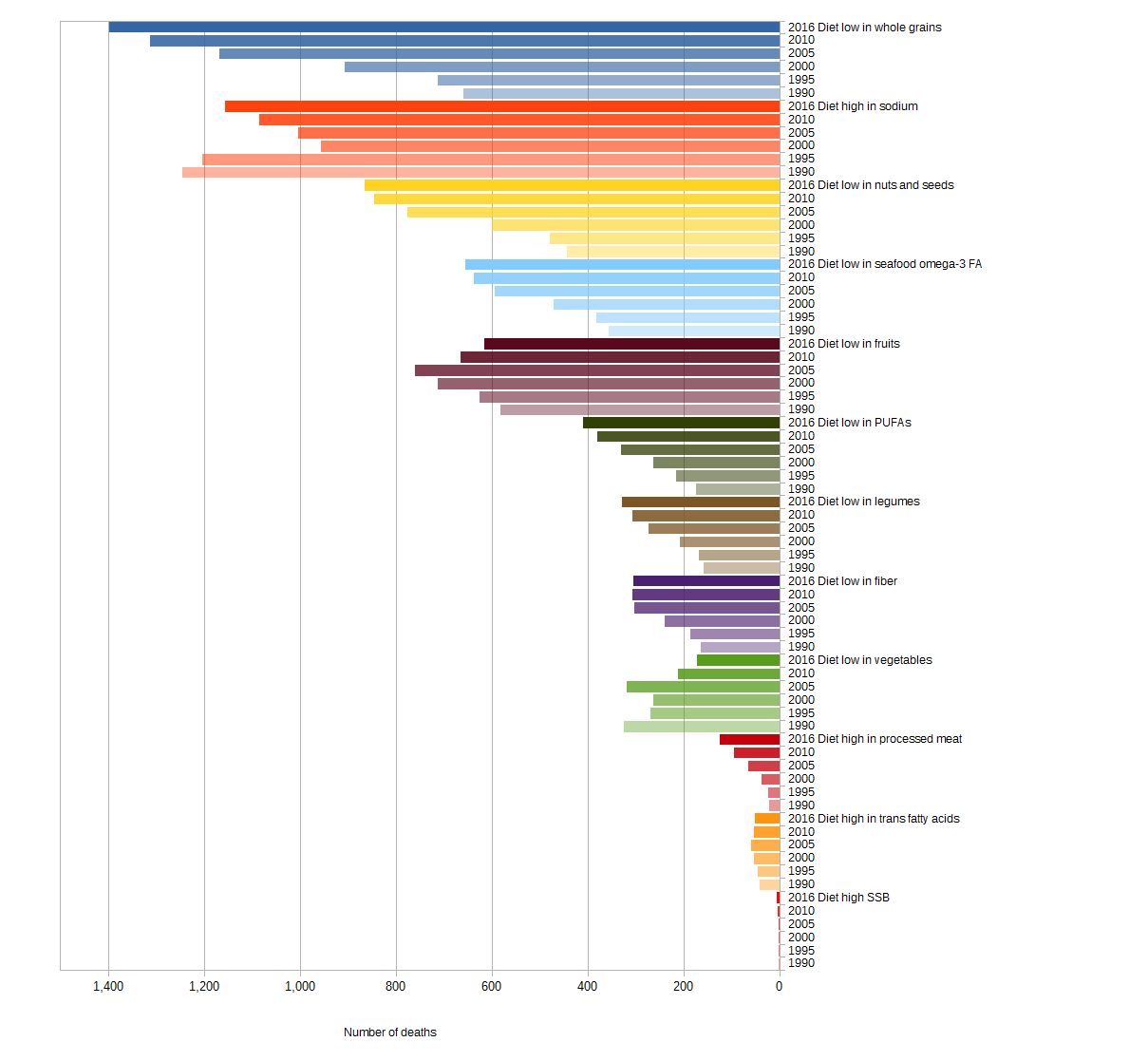
**
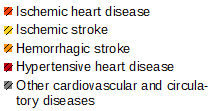
Albania**

**
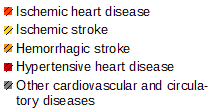

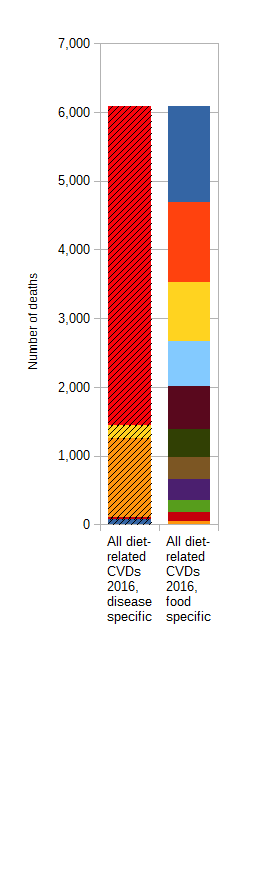
**

Figure 1 Diet-related CVD deaths from 1990 to 2016 in Albania

a) Male b) Female


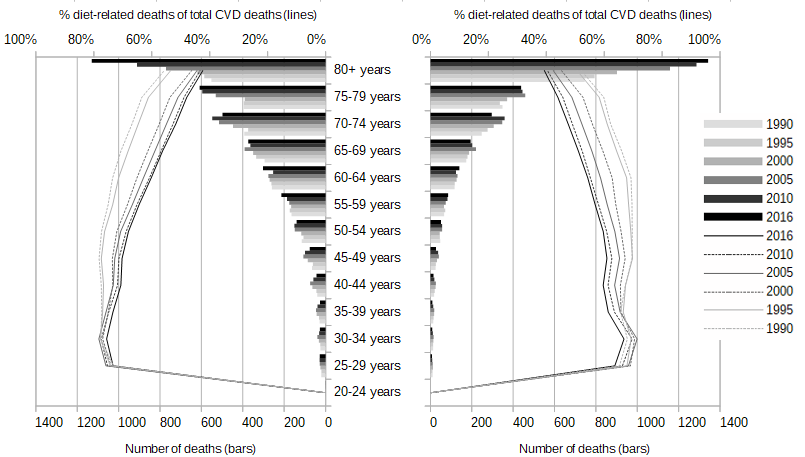


Figure 2 Age- and gender-specific diet-related CVD deaths (bars) and share of diet-related on total CVD deaths (lines) from 1990 to 2016 in Albania

**
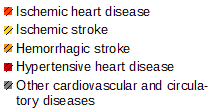
**
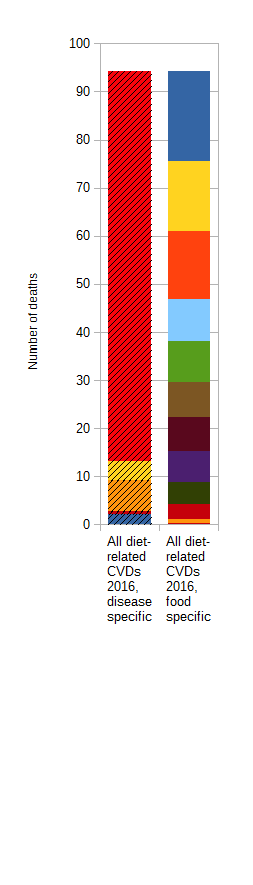

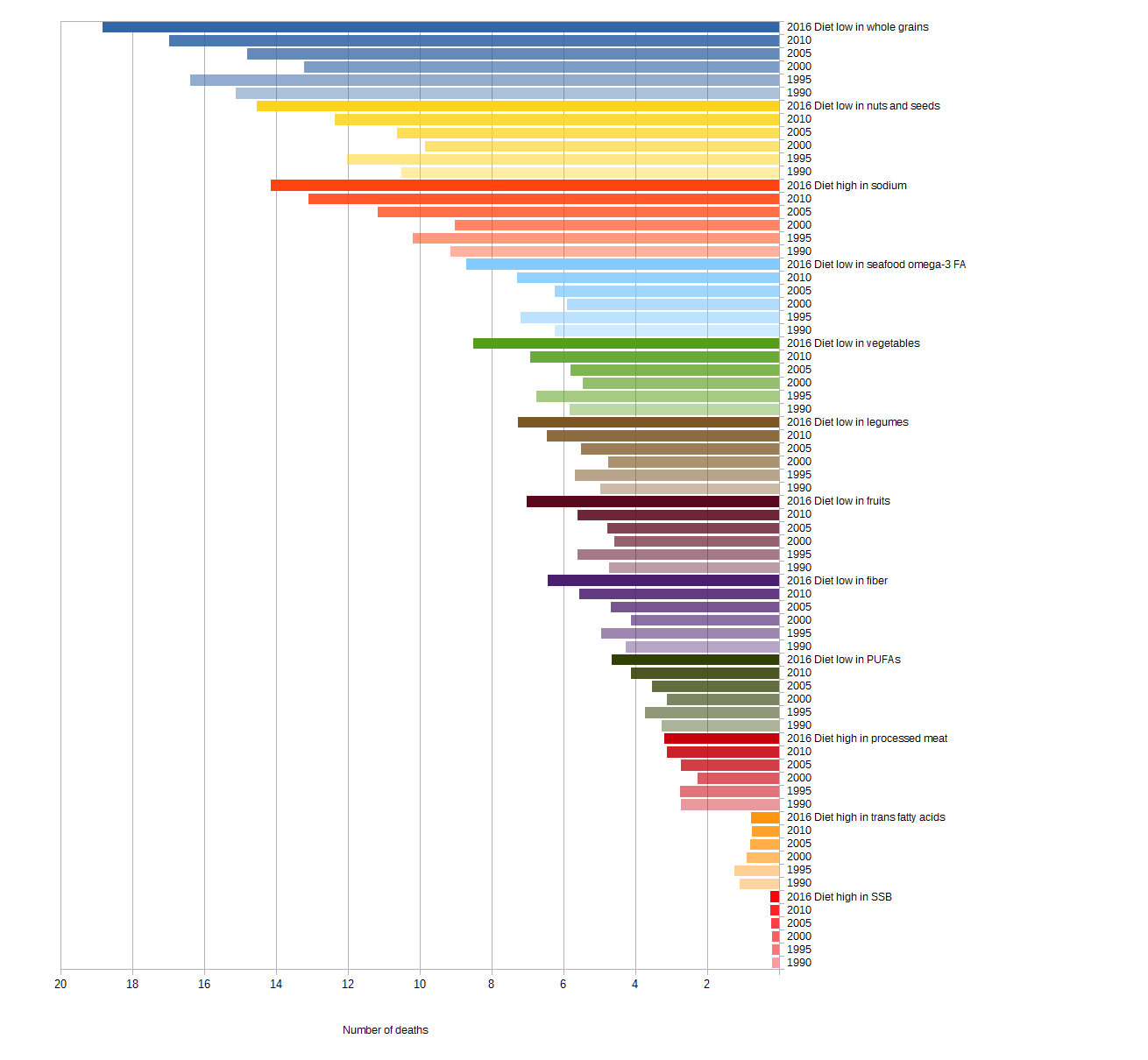
**Andorra**

Figure 3 Diet-related CVD deaths from 1990 to 2016 in Andorra

a) Male b) Female


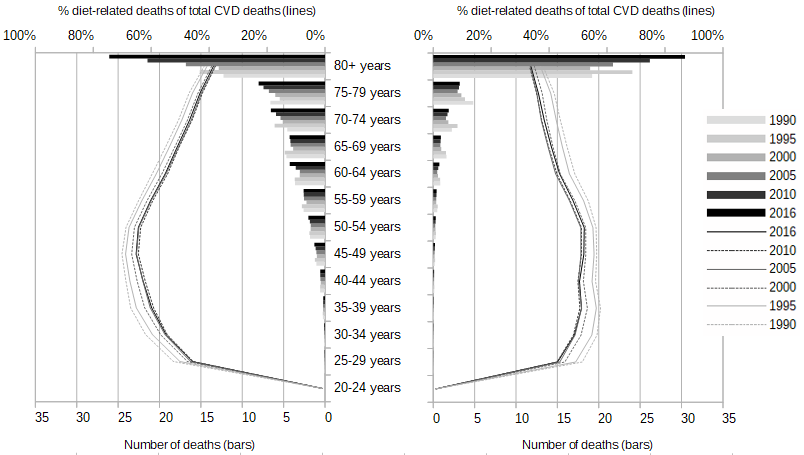


Figure 4 Age- and gender-specific diet-related CVD deaths (bars) and share of diet-related on total CVD deaths (lines) from 1990 to 2016 in Andorra

**
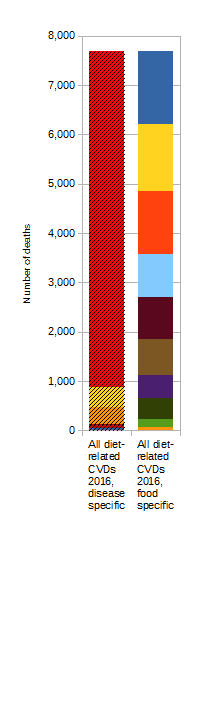

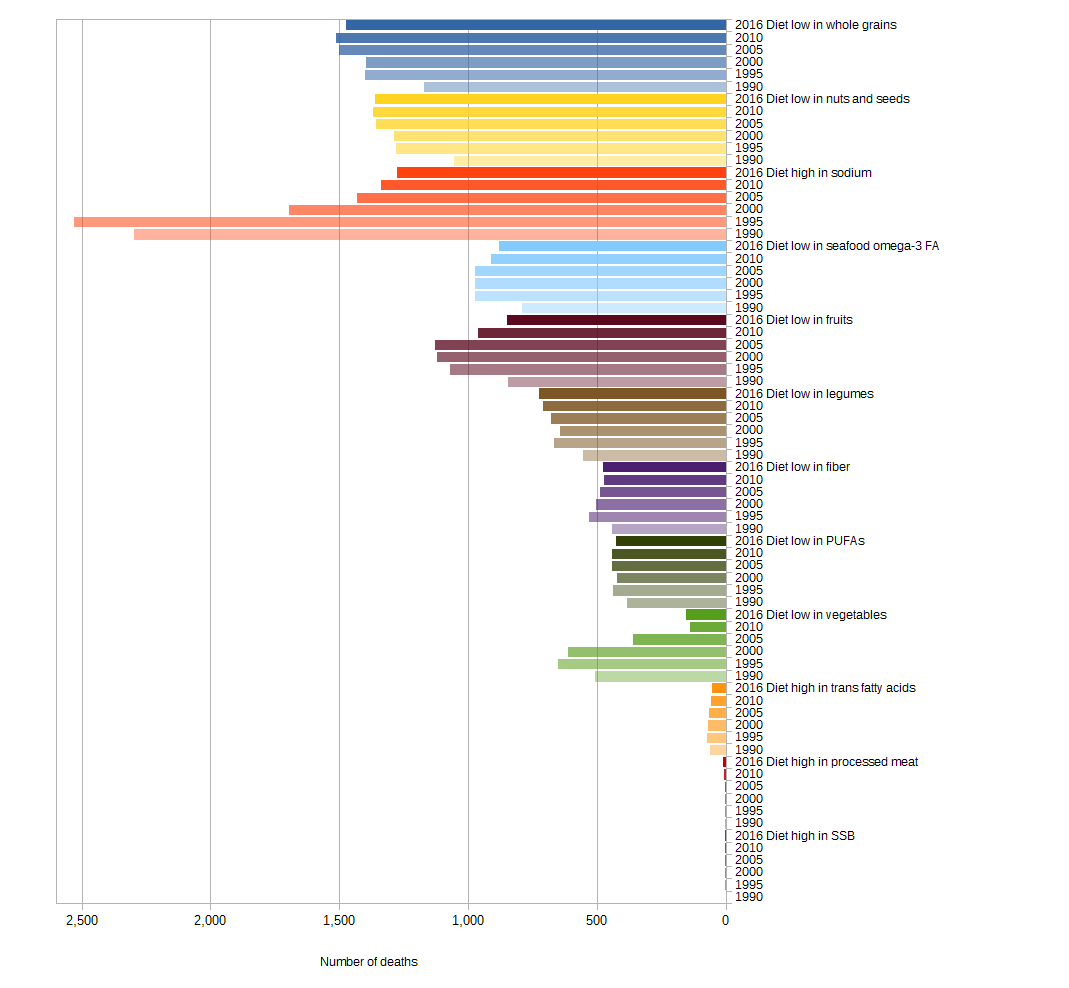

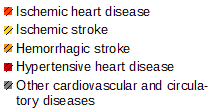
Armenia**

Figure 5 Diet-related CVD deaths from 1990 to 2016 in Armenia

a) Male b) Female


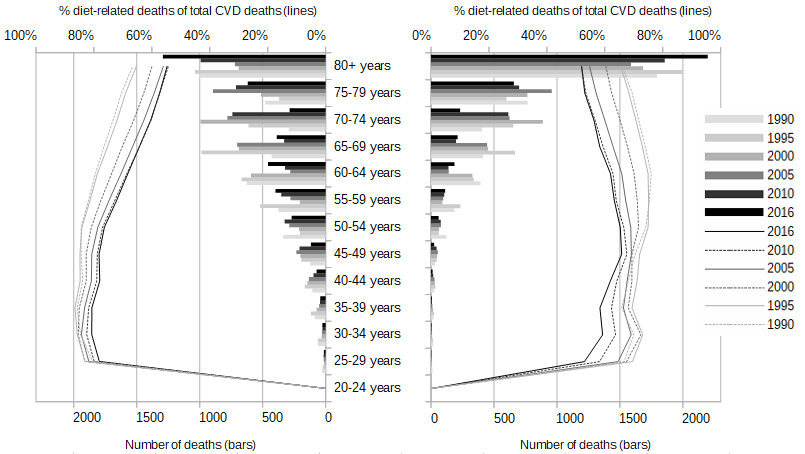


Figure 6 Age- and gender-specific diet-related CVD deaths (bars) and share of diet-related on total CVD deaths (lines) from 1990 to 2016 in Armenia

**
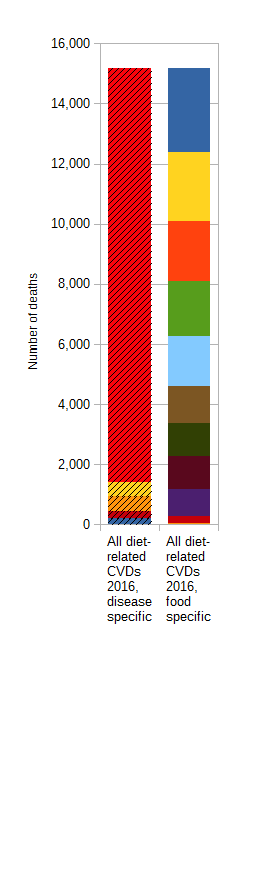

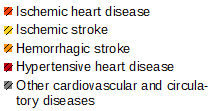

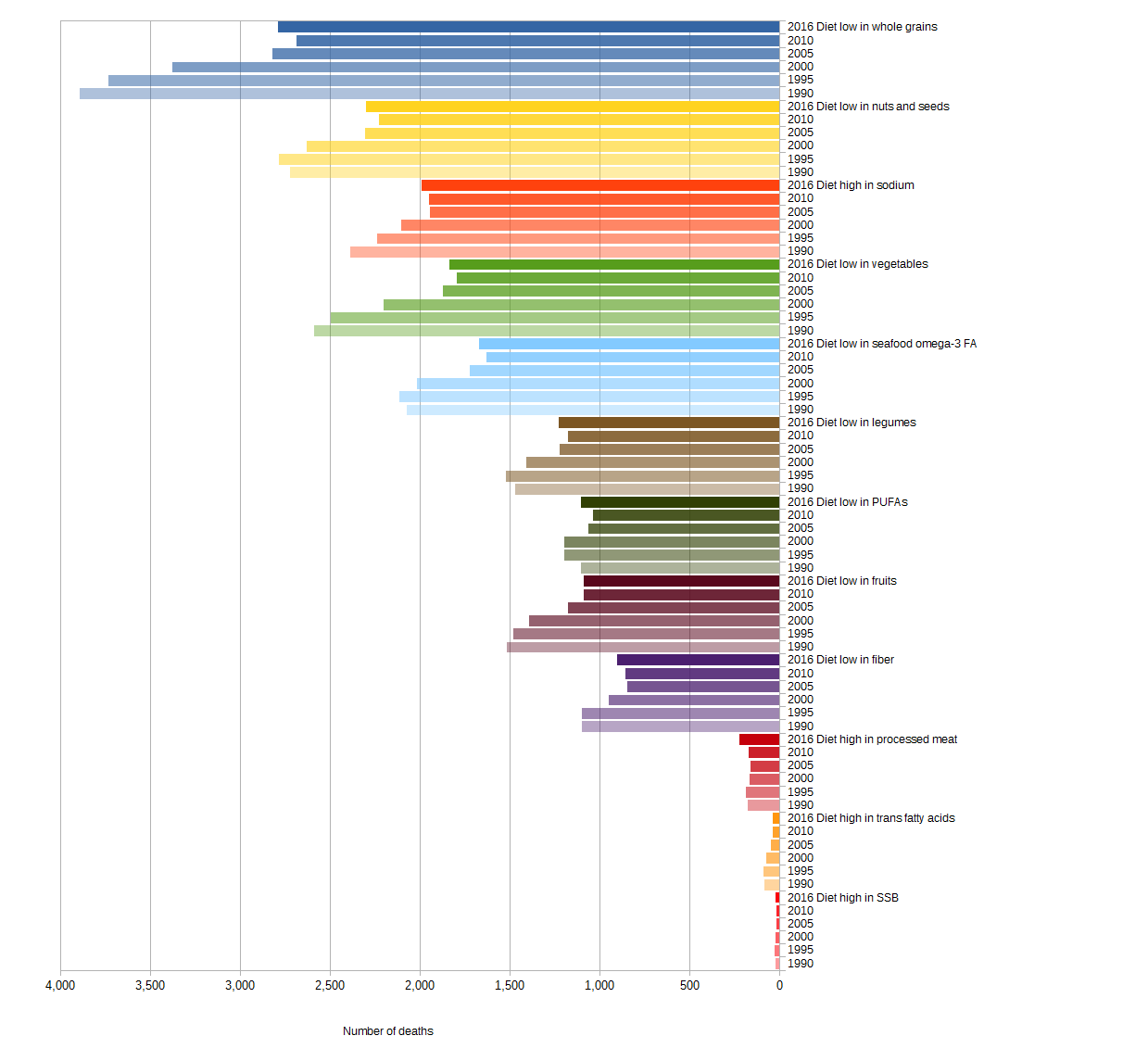
Austria**

Figure 7 Diet-related CVD deaths from 1990 to 2016 in Austria

a) Male b) Female


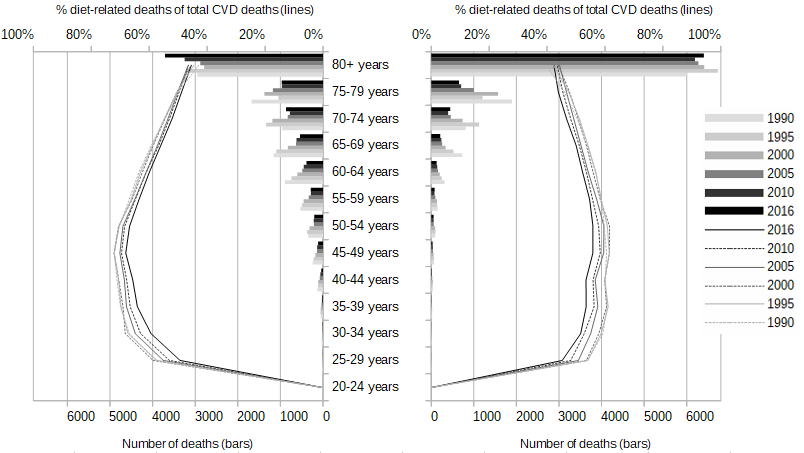


Figure 8 Age- and gender-specific diet-related CVD deaths (bars) and share of diet-related on total CVD deaths (lines) from 1990 to 2016 in Austria

**
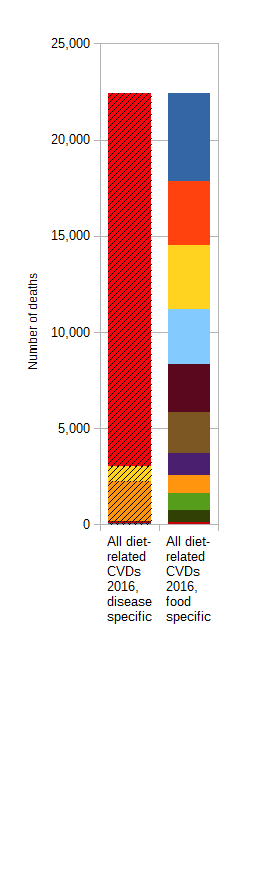

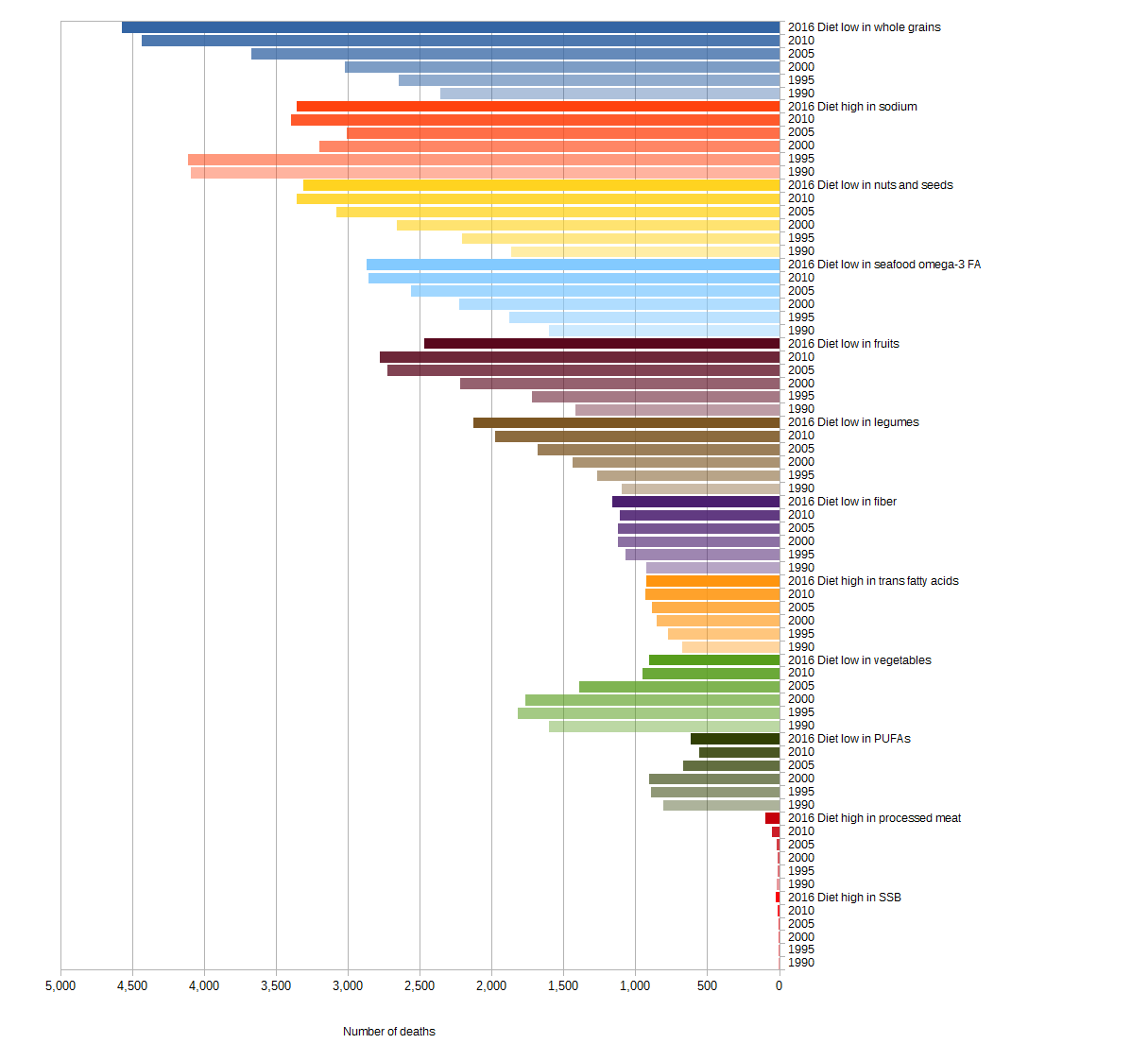

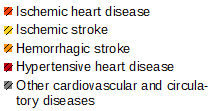
Azerbaijan**

Figure 9 Diet-related CVD deaths from 1990 to 2016 in Azerbaijan

a) Male b) Female


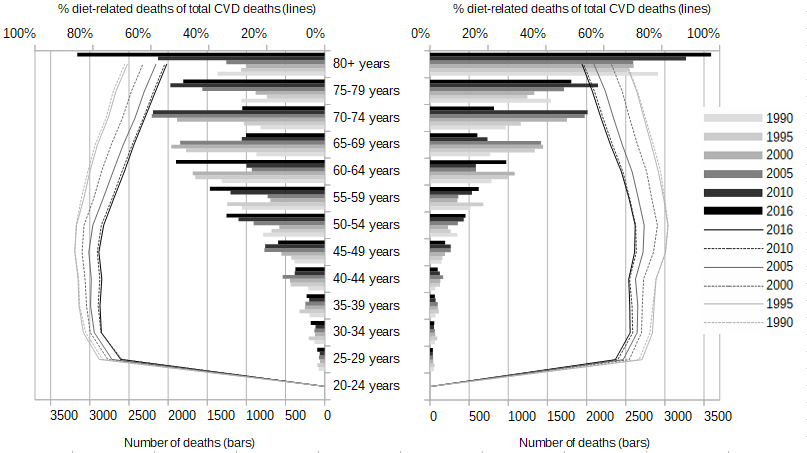


Figure 10 Age- and gender-specific diet-related CVD deaths (bars) and share of diet-related on total CVD deaths (lines) from 1990 to 2016 in Azerbaijan

**
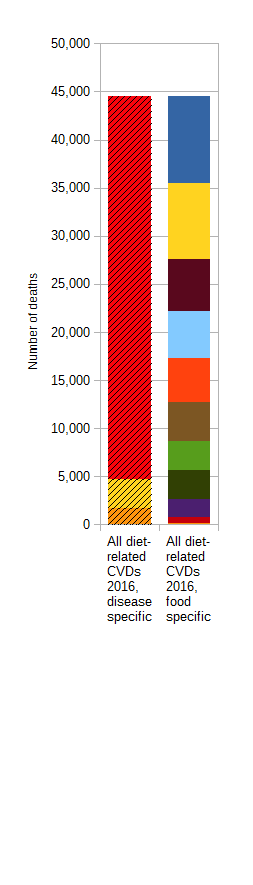

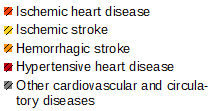

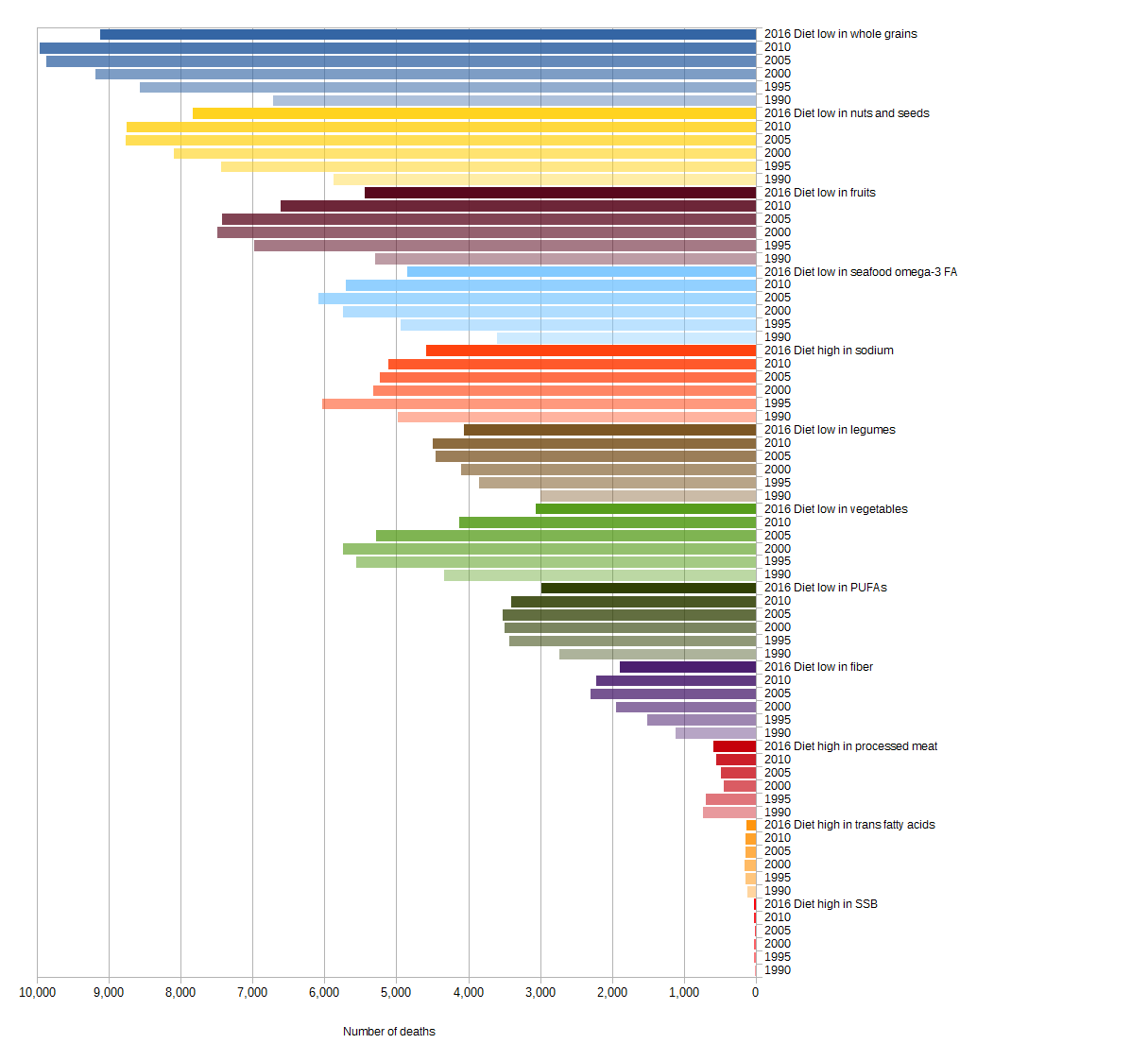
Belarus**

Figure 11 Diet-related CVD deaths from 1990 to 2016 in Belarus

a) Male b) Female


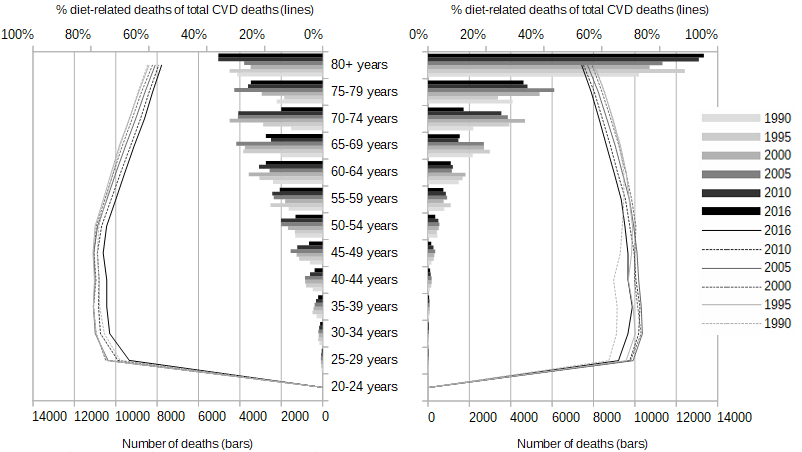


Figure 12 Age- and gender-specific diet-related CVD deaths (bars) and share of diet-related on total CVD deaths (lines) from 1990 to 2016 in Belarus

**
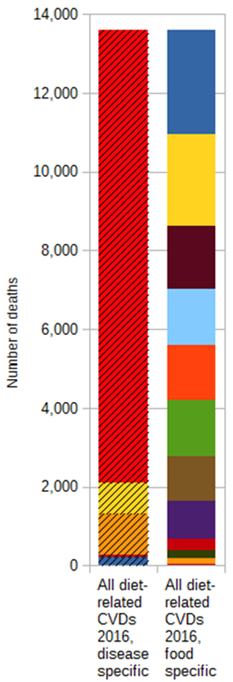

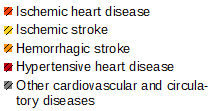
**
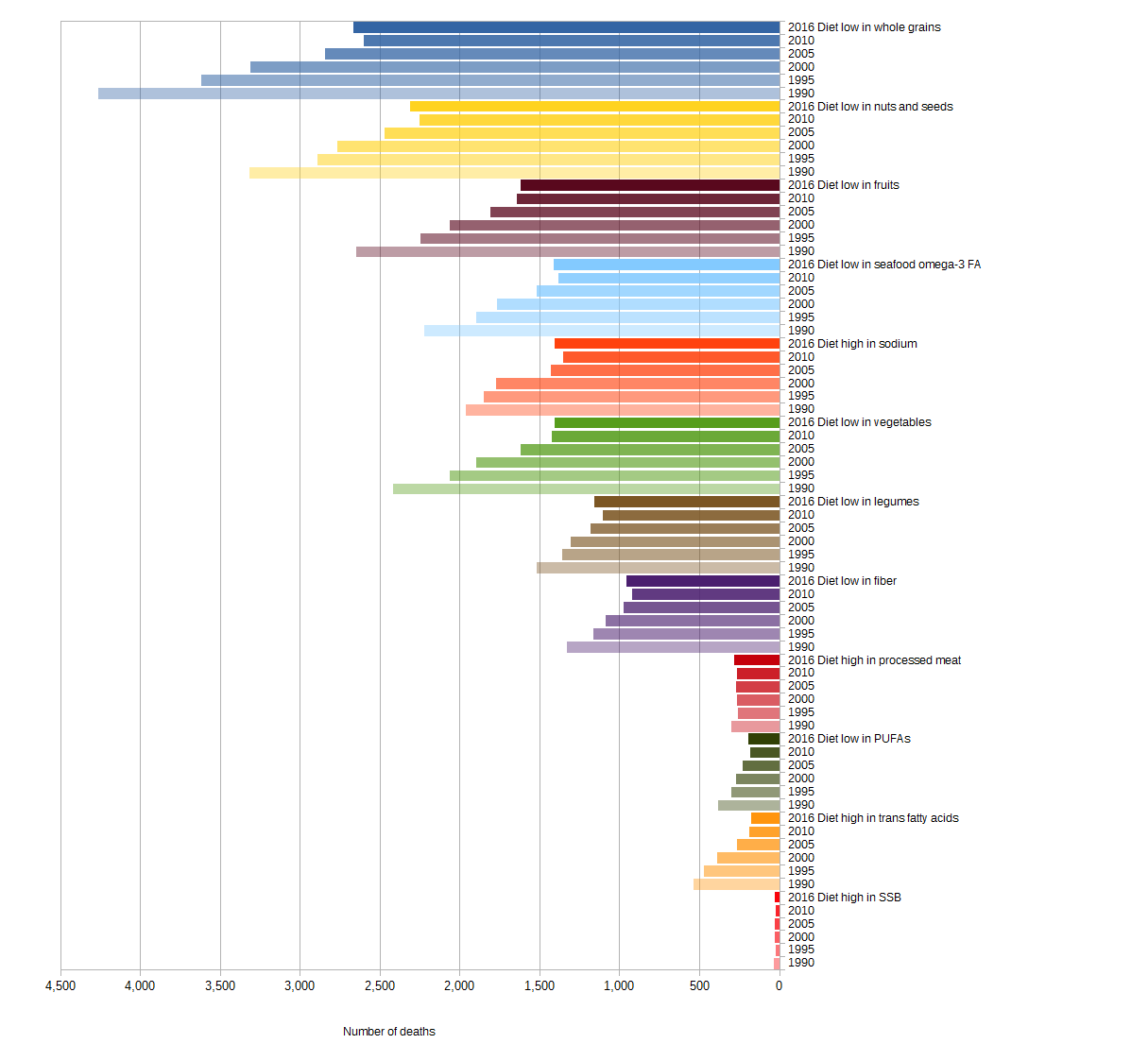
**Belgium**

Figure 13 Diet-related CVD deaths from 1990 to 2016 in Belgium

a) Male b) Female


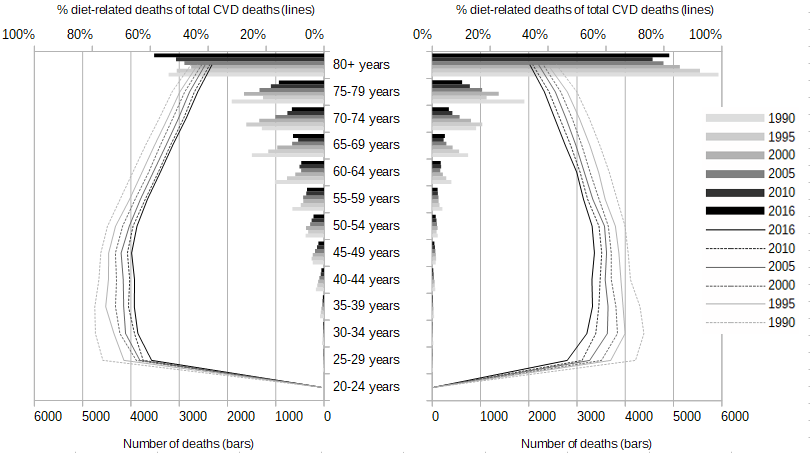


Figure 14 Age- and gender-specific diet-related CVD deaths (bars) and share of diet-related on total CVD deaths (lines) from 1990 to 2016 in Belgium

**
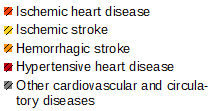

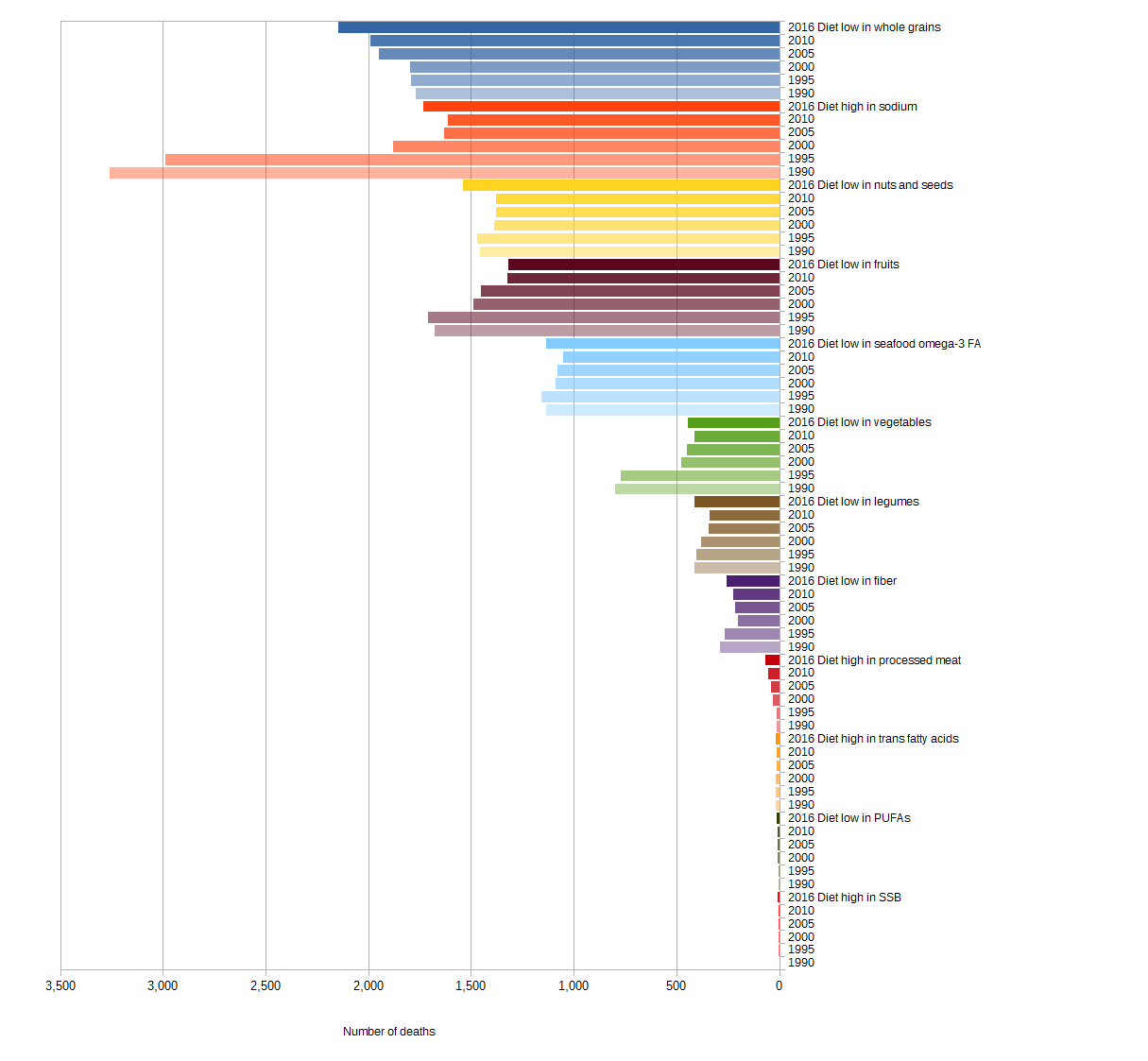

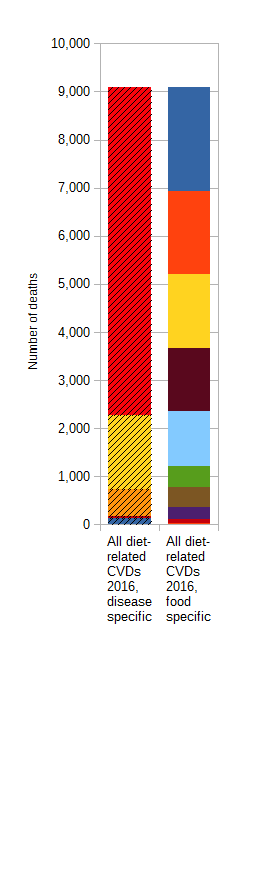
Bosnia and Herzegovina**

Figure 15 Diet-related CVD deaths from 1990 to 2016 in Bosnia and Herzegovina

a) Male b) Female


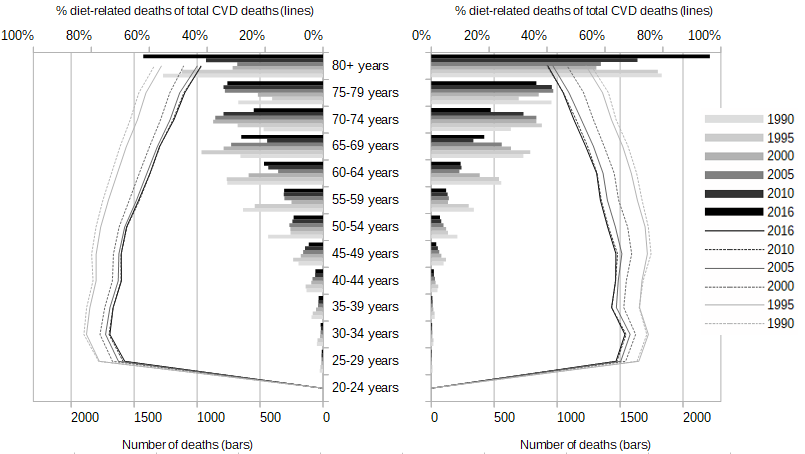


Figure 16 Age- and gender-specific diet-related CVD deaths (bars) and share of diet-related on total CVD deaths (lines) from 1990 to 2016 in Bosnia and Herzegovina


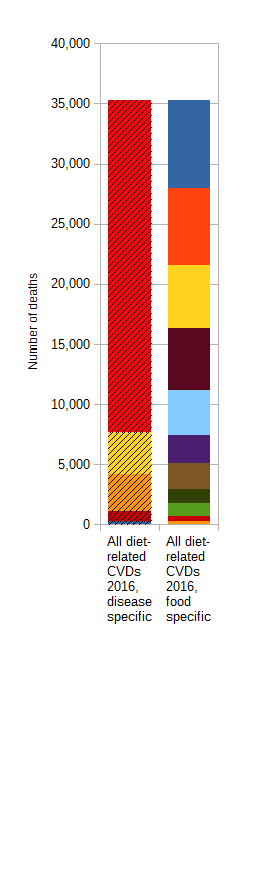
**
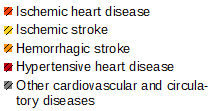
**
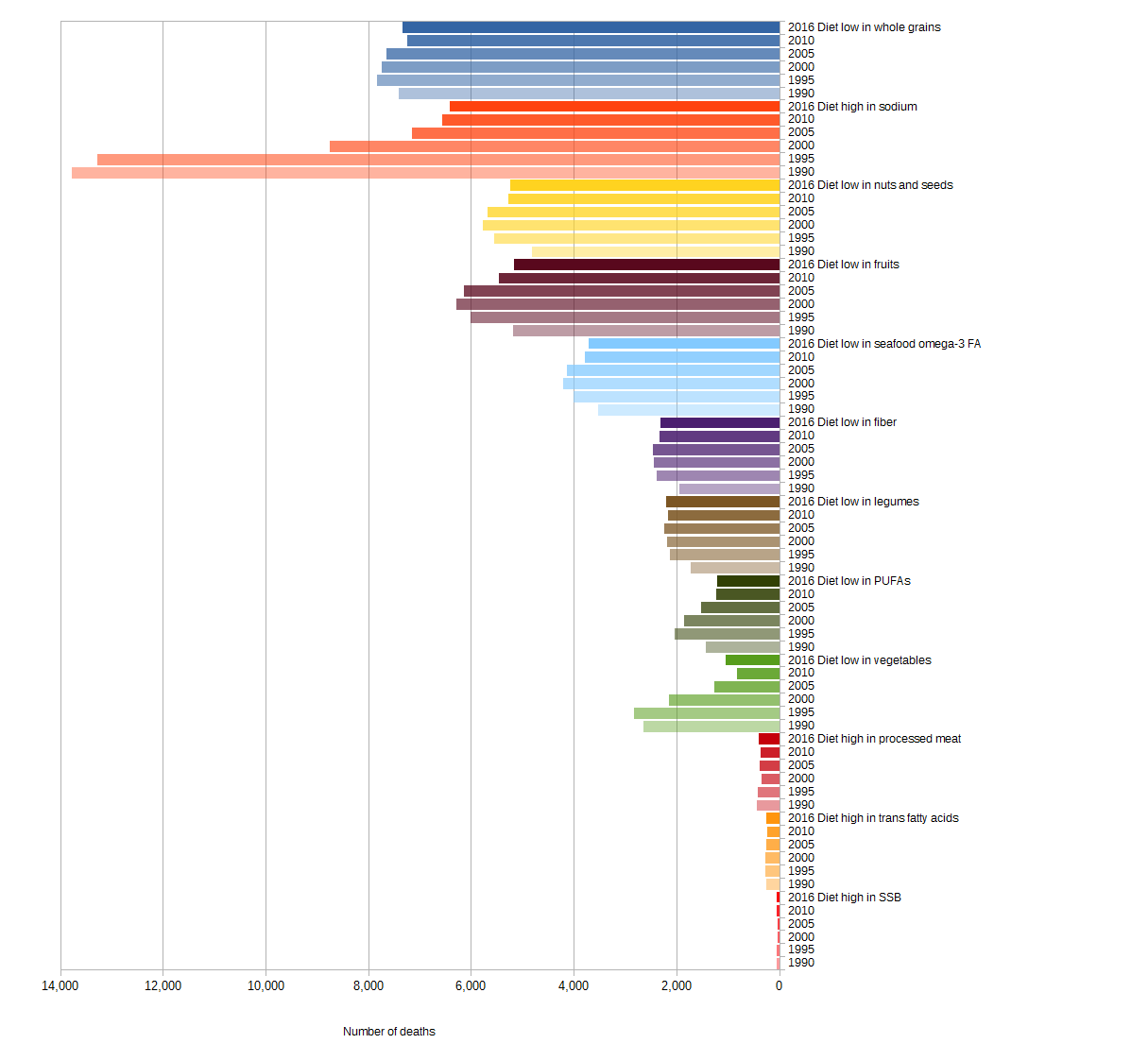
**Bulgaria**

Figure 17 Diet-related CVD deaths from 1990 to 2016 in Bulgaria

a) Male b) Female


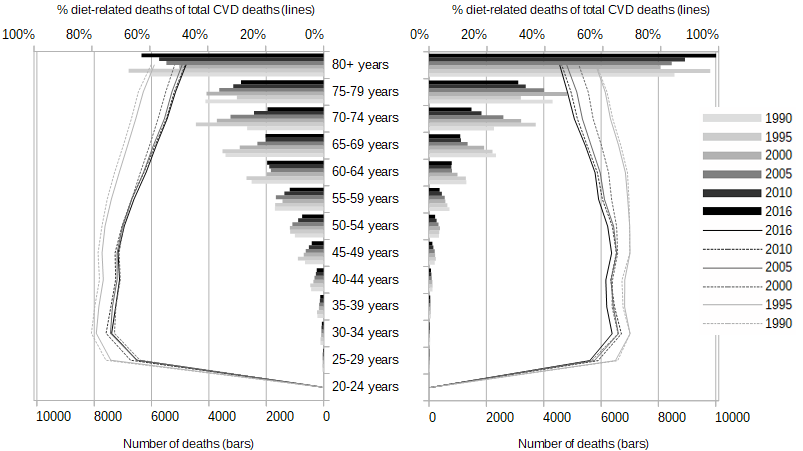


Figure 18 Age- and gender-specific diet-related CVD deaths (bars) and share of diet-related on total CVD deaths (lines) from 1990 to 2016 in Bulgaria

**
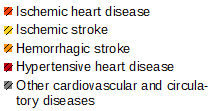
**
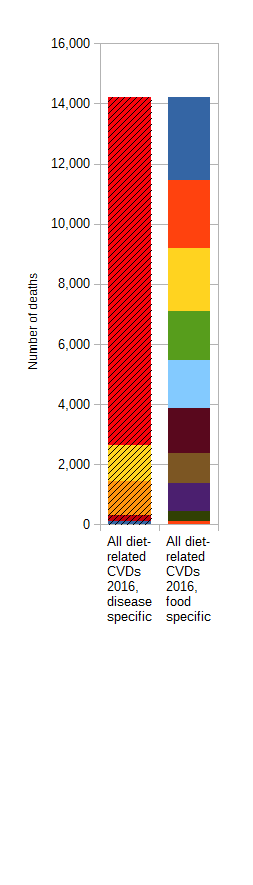
**
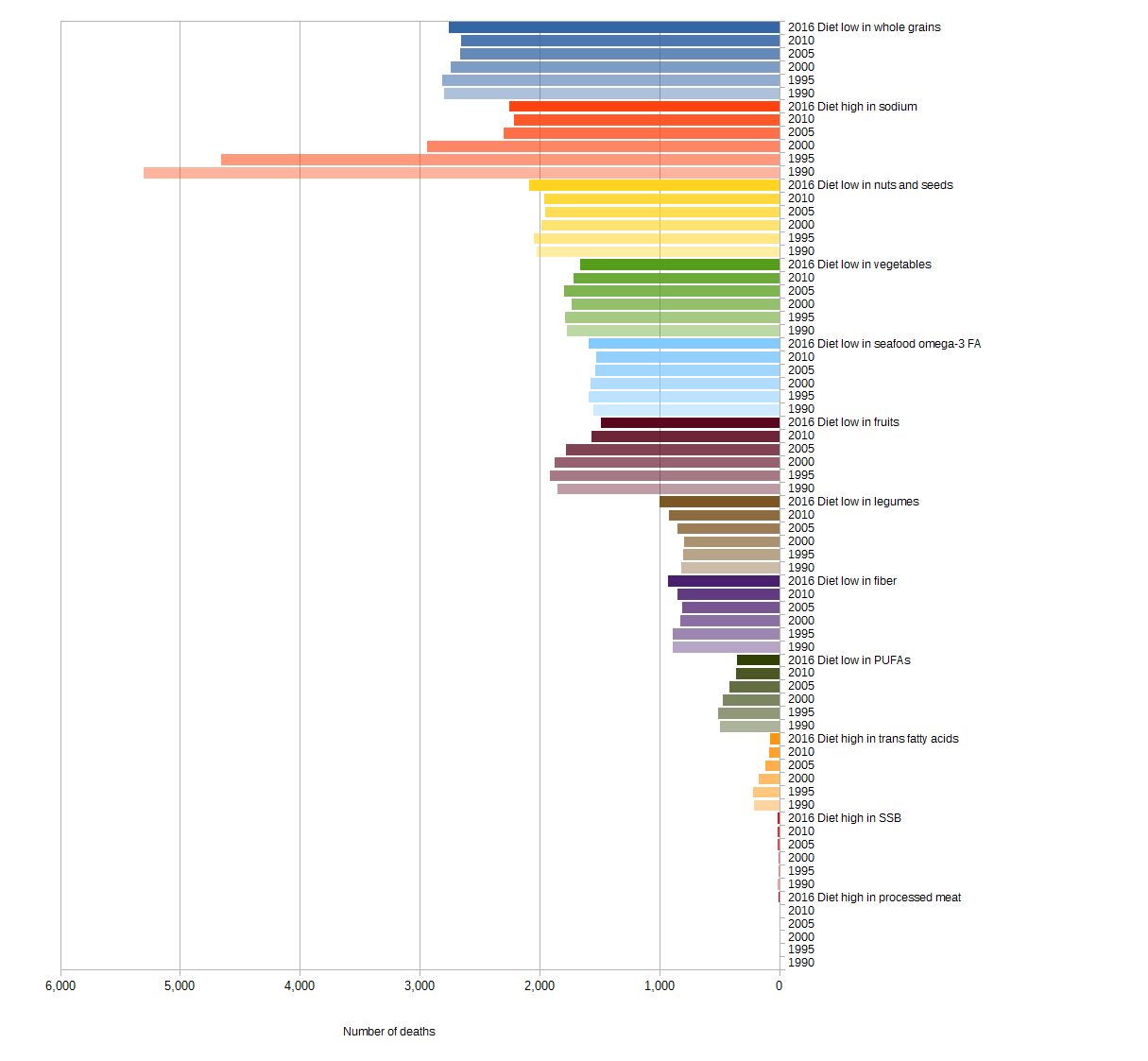
Croatia**

Figure 19 Diet-related CVD deaths from 1990 to 2016 in Croatia

a) Male b) Female


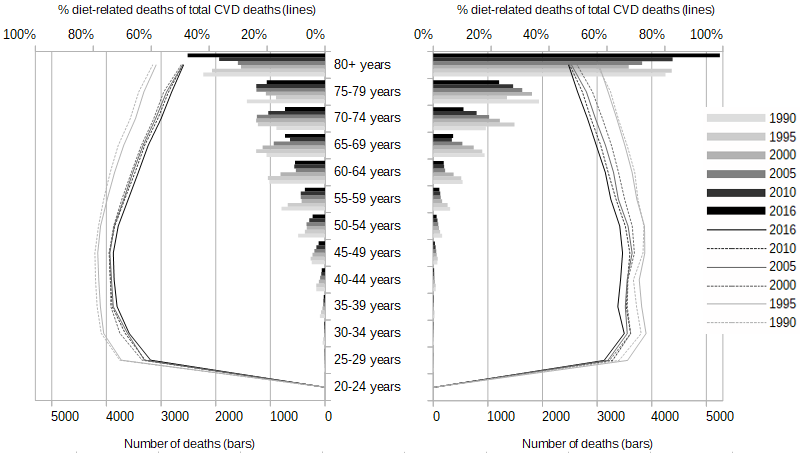


Figure 20 Age- and gender-specific diet-related CVD deaths (bars) and share of diet-related on total CVD deaths (lines) from 1990 to 2016 in Croatia

**
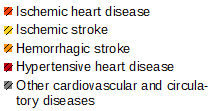
**
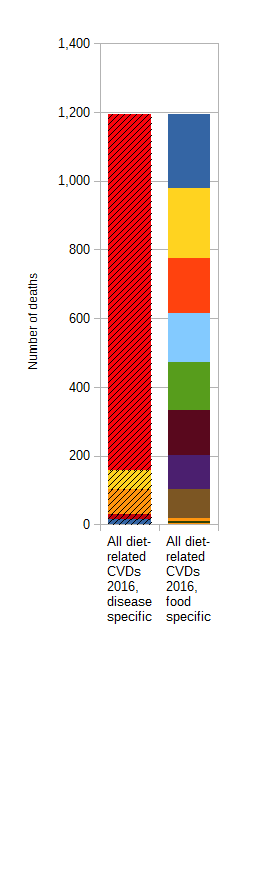
**
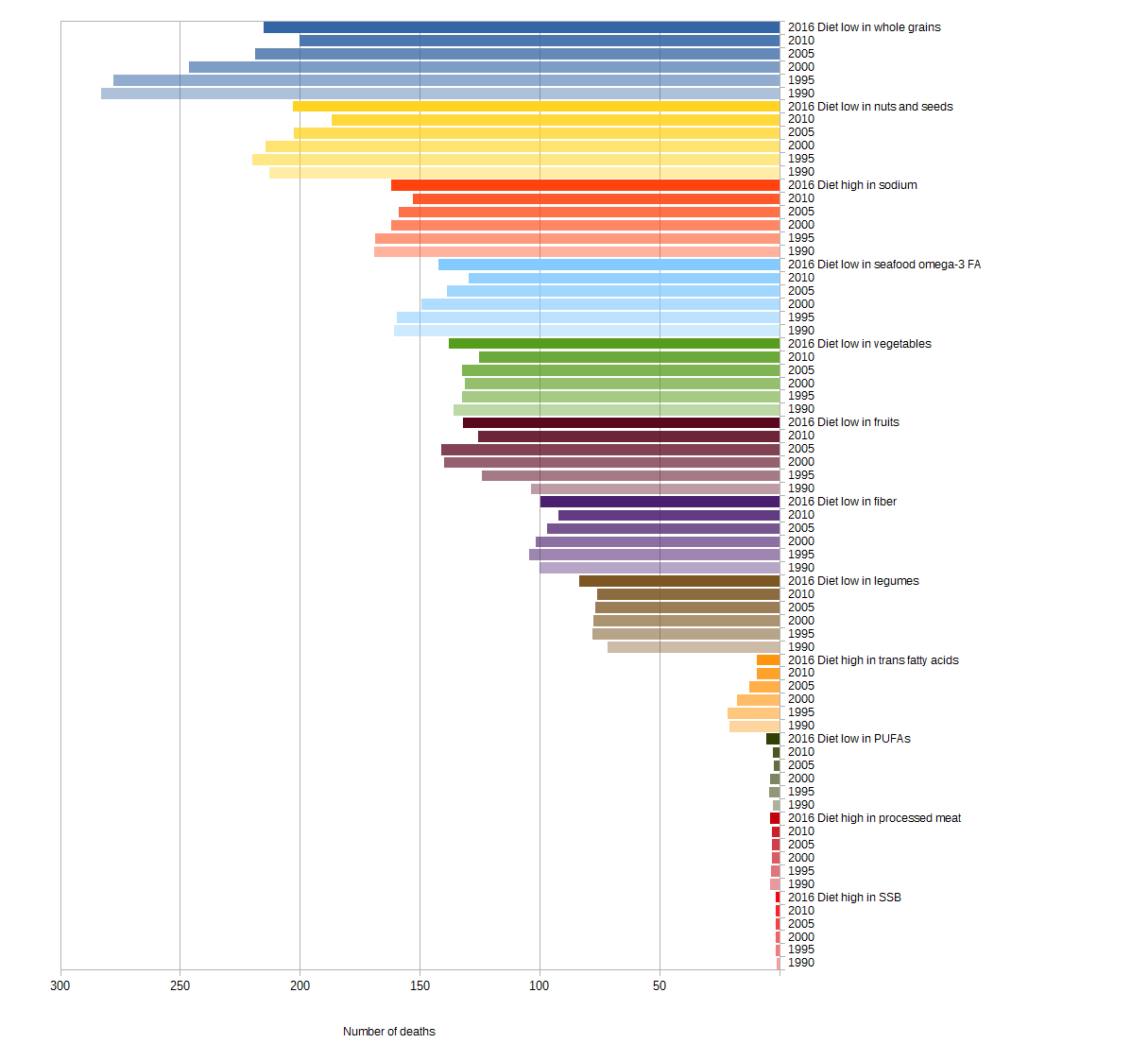
Cyprus**

Figure 21 Diet-related CVD deaths from 1990 to 2016 in Cyprus

a) Male b) Female


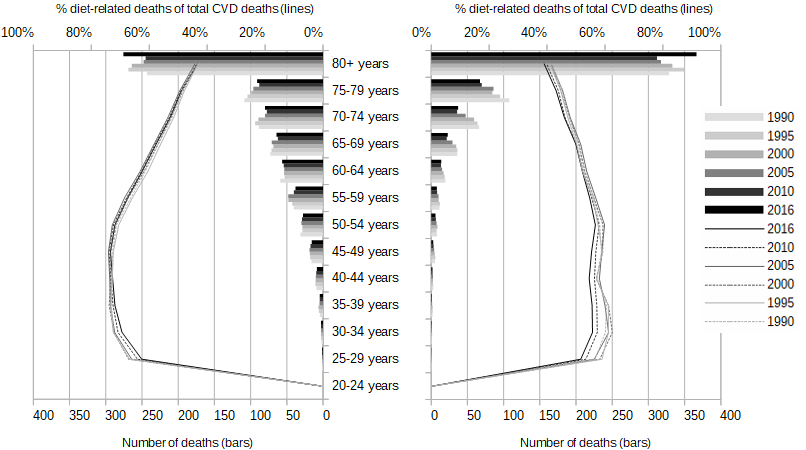


Figure 22 Age- and gender-specific diet-related CVD deaths (bars) and share of diet-related on total CVD deaths (lines) from 1990 to 2016 in Cyprus

**
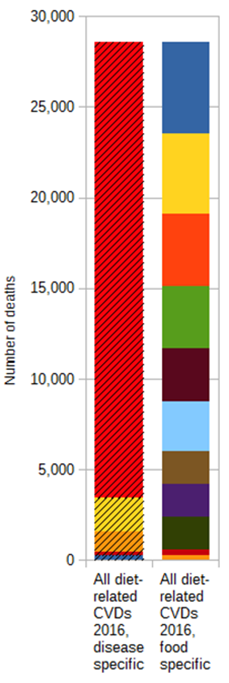

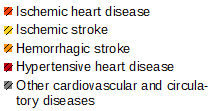
**
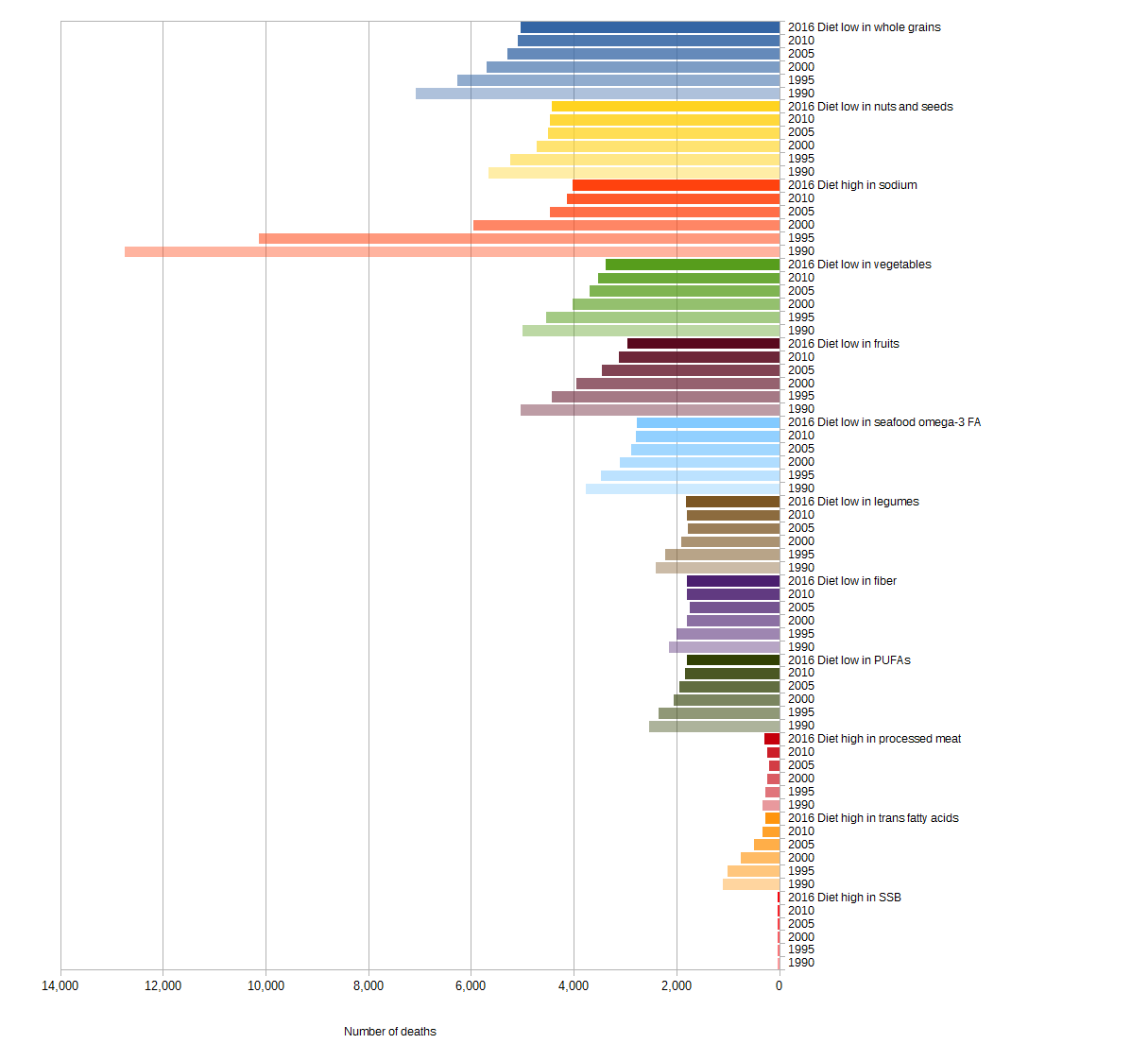
**Czech Republic**

Figure 23 Diet-related CVD deaths from 1990 to 2016 in the Czech Republic

a) Male b) Female


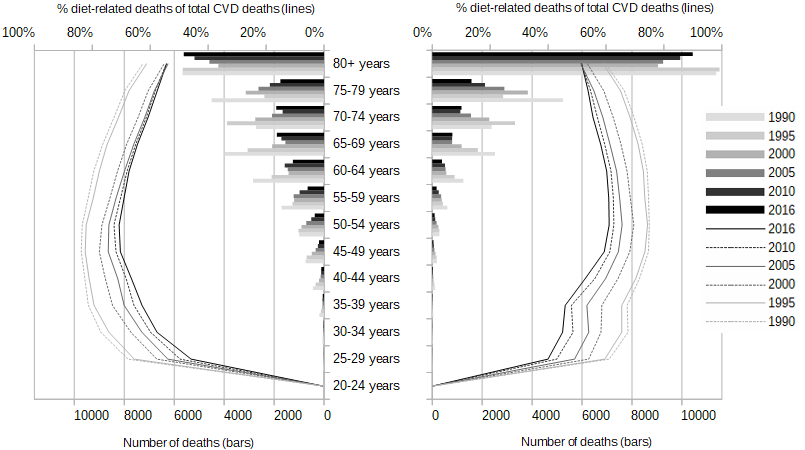


Figure 24 Age- and gender-specific diet-related CVD deaths (bars) and share of diet-related on total CVD deaths (lines) from 1990 to 2016 in the Czech Republic

**
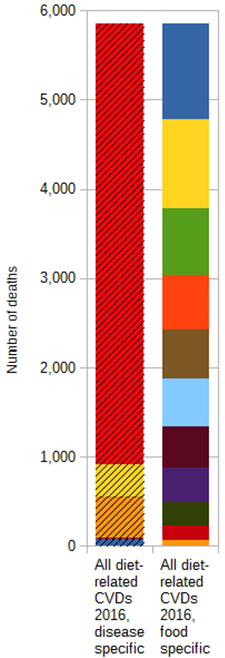

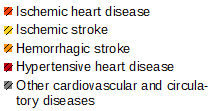

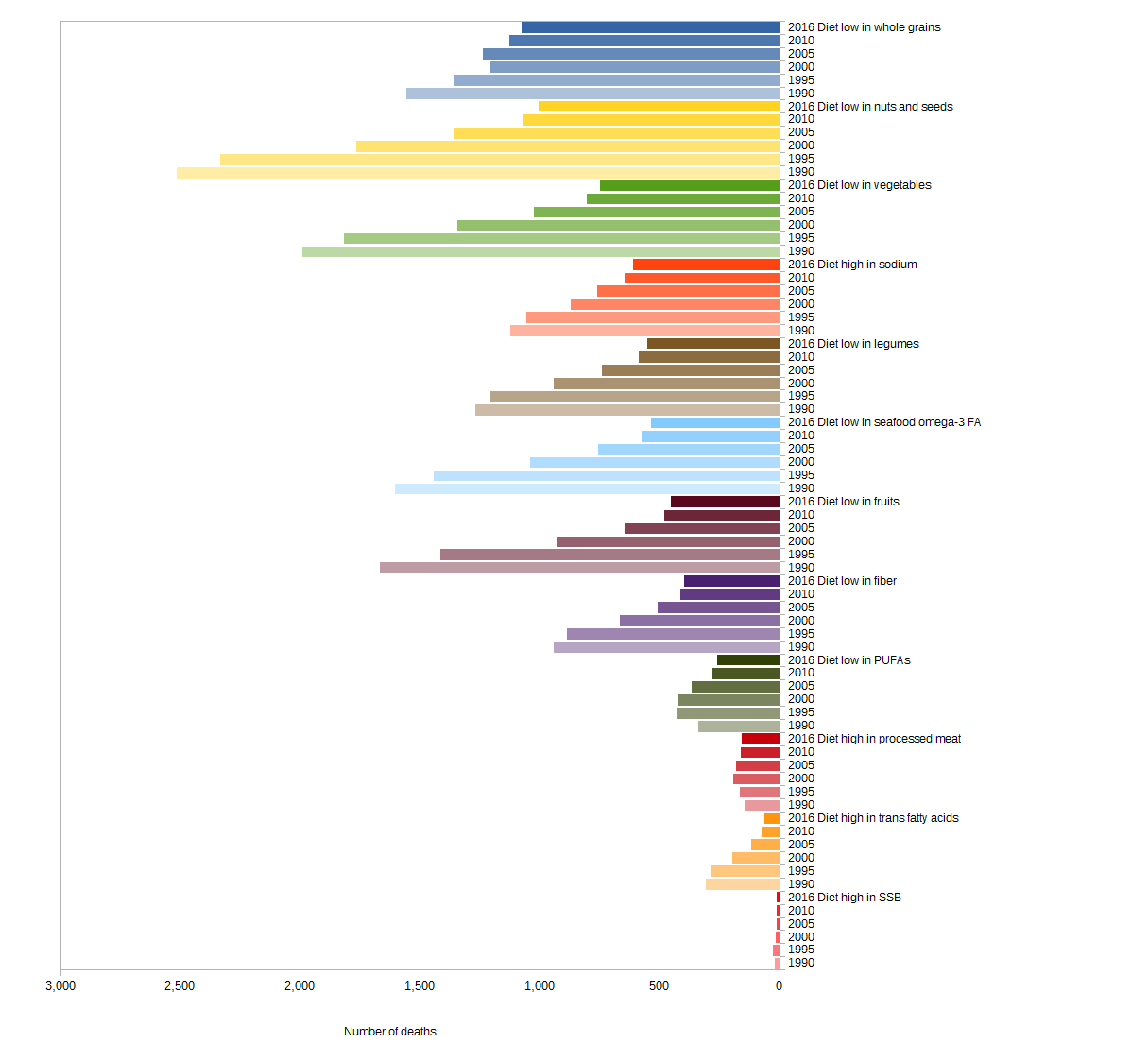
Denmark**

Figure 25 Diet-related CVD deaths from 1990 to 2016 in Denmark

a) Male b) Female


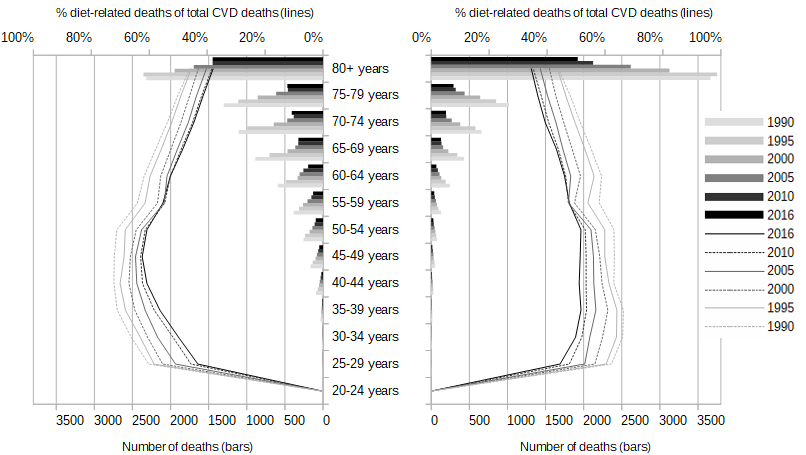


Figure 26 Age- and gender-specific diet-related CVD deaths (bars) and share of diet-related on total CVD deaths (lines) from 1990 to 2016 in Denmark

**
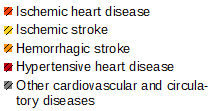

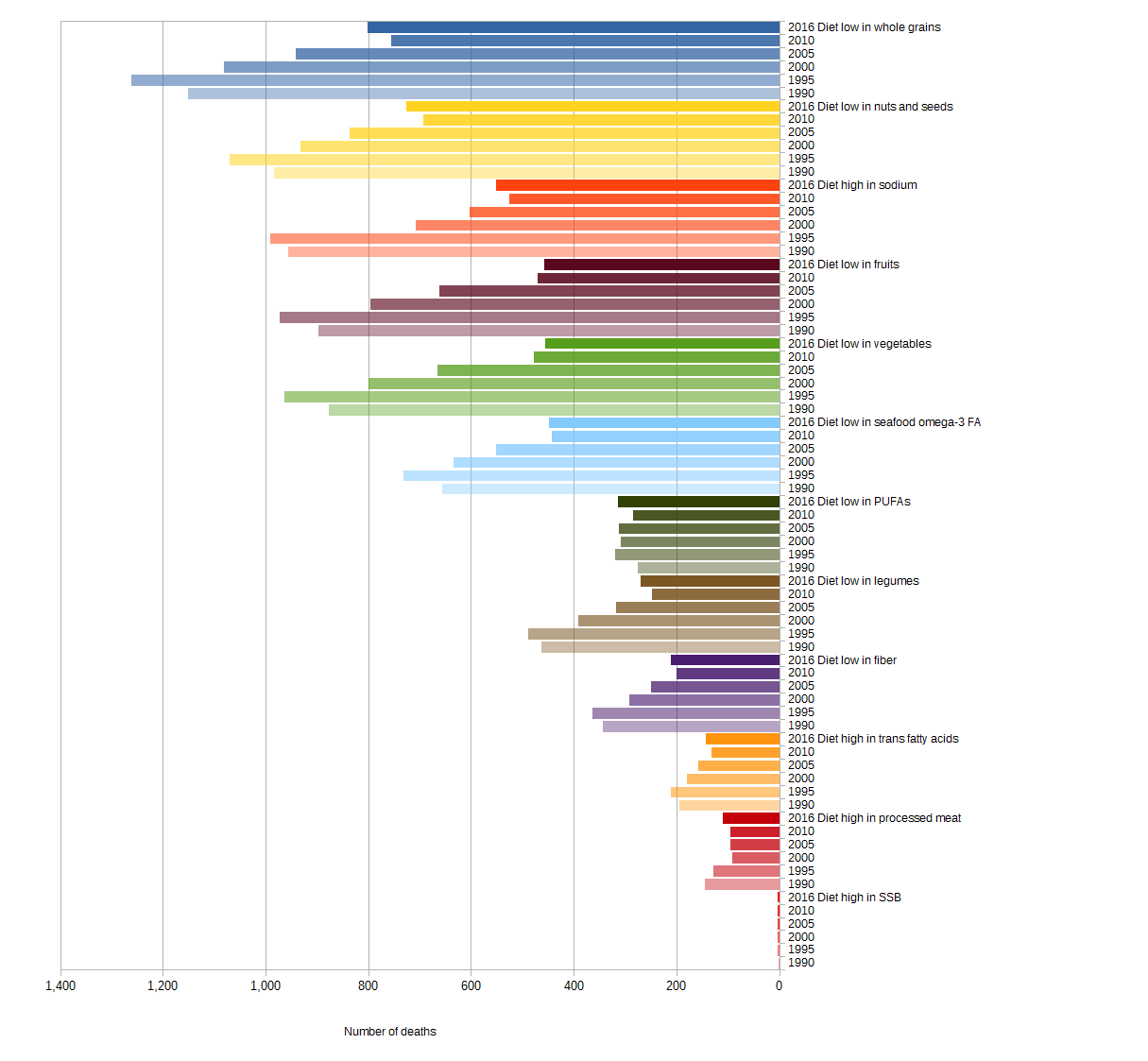

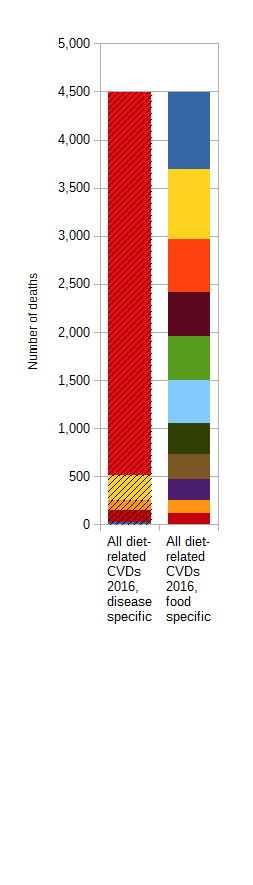
Estonia**

Figure 27 Diet-related CVD deaths from 1990 to 2016 in Estonia

a) Male b) Female


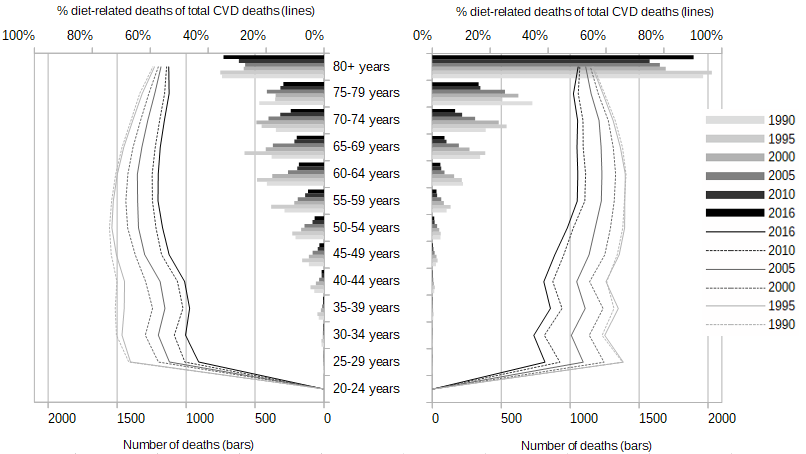


Figure 28 Age- and gender-specific diet-related CVD deaths (bars) and share of diet-related on total CVD deaths (lines) from 1990 to 2016 in Estonia

**
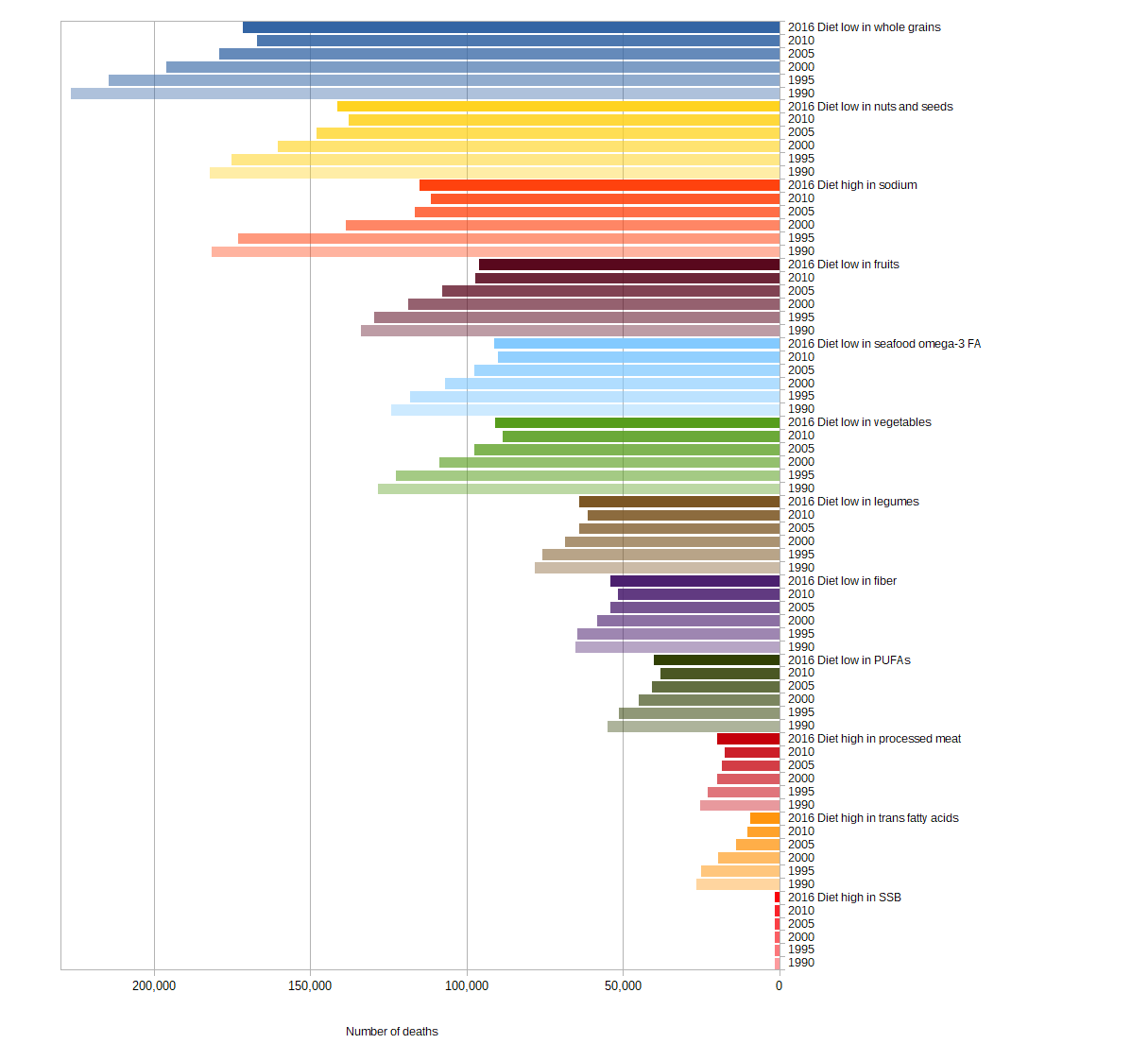

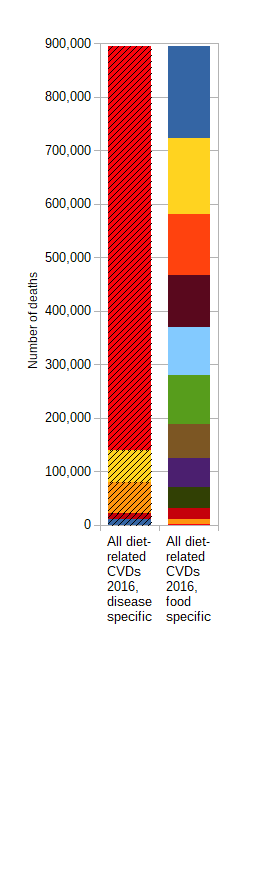

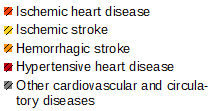
EU-28**

Figure 29 Diet-related CVD deaths from 1990 to 2016 in the EU-28

a) Male b) Female


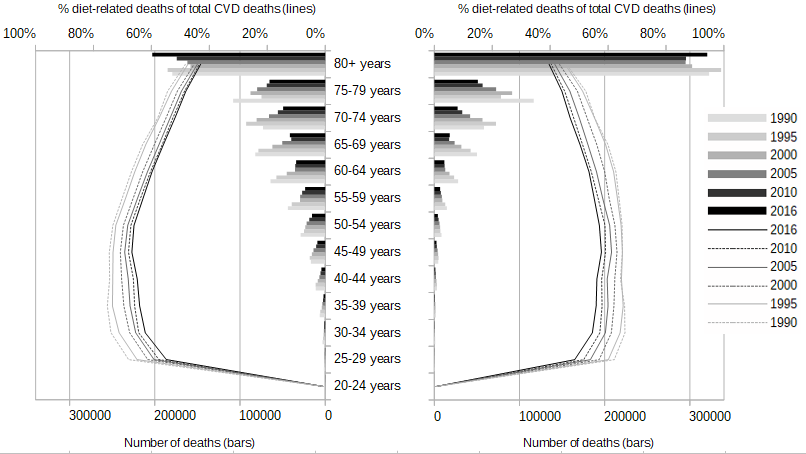


**Figure 30 Age- and gender-specific diet-related CVD deaths (bars) and share of diet-related on total CVD deaths (lines) from 1990 to 2016 in the EU-28**

**
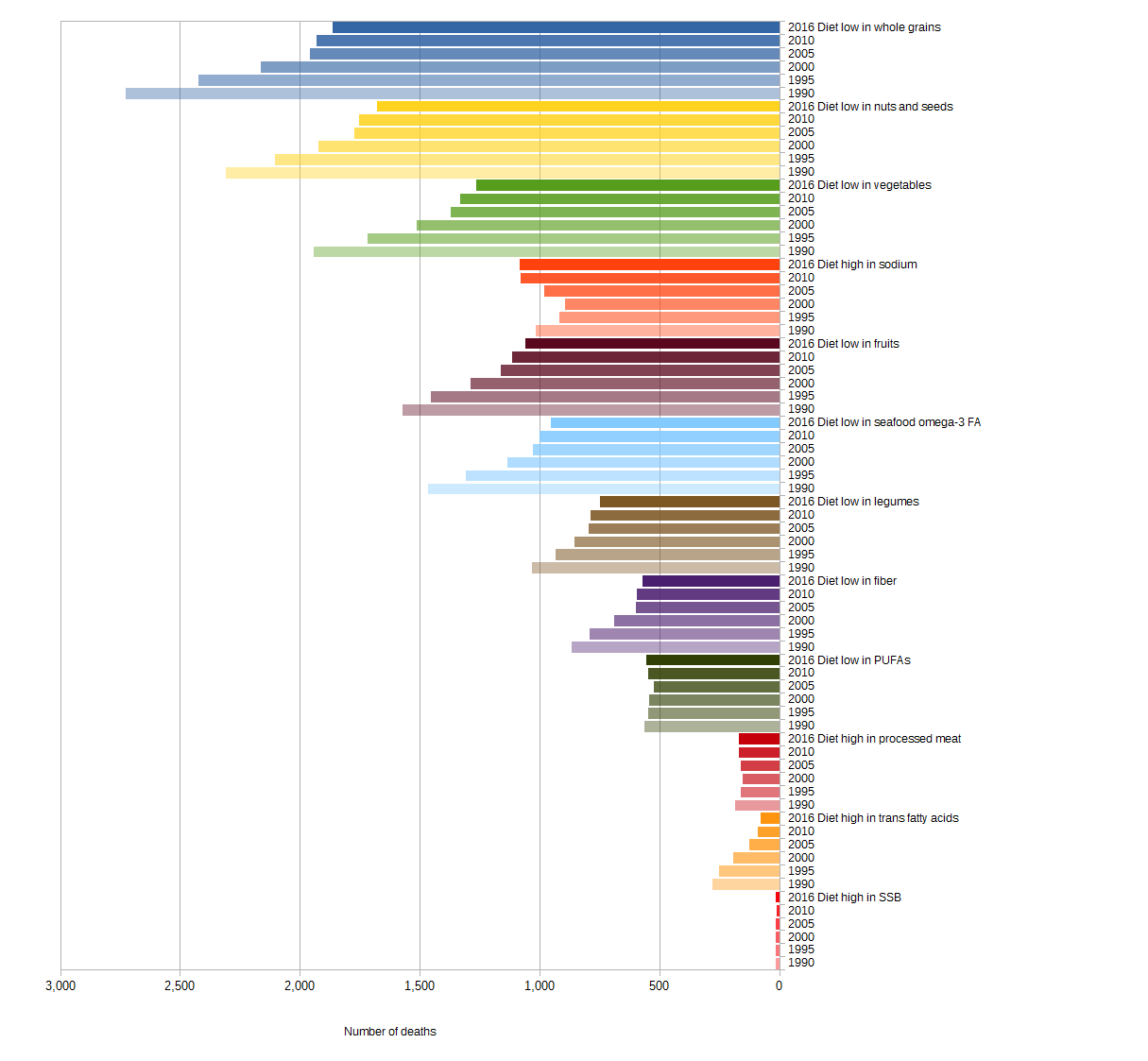

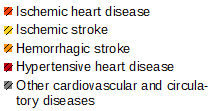

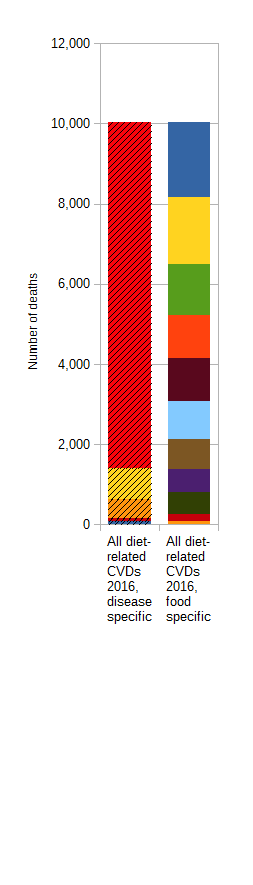
Finland**

Figure 31 Diet-related CVD deaths from 1990 to 2016 in Finland

a) Male b) Female


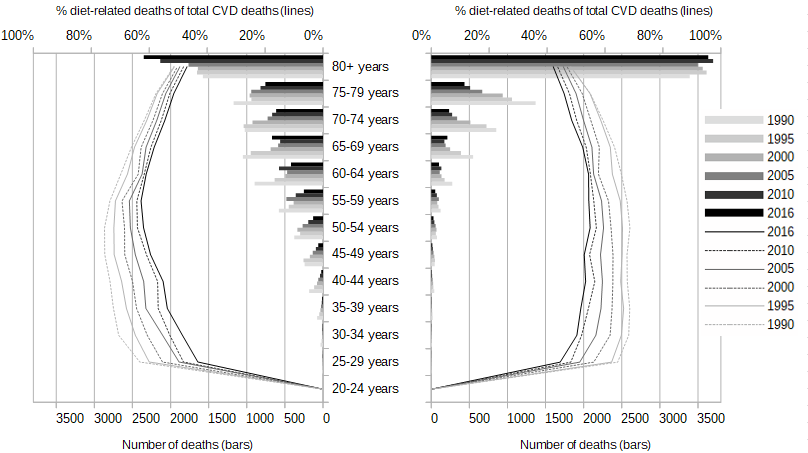


Figure 32 Age- and gender-specific diet-related CVD deaths (bars) and share of diet-related on total CVD deaths (lines) from 1990 to 2016 in Finland

**
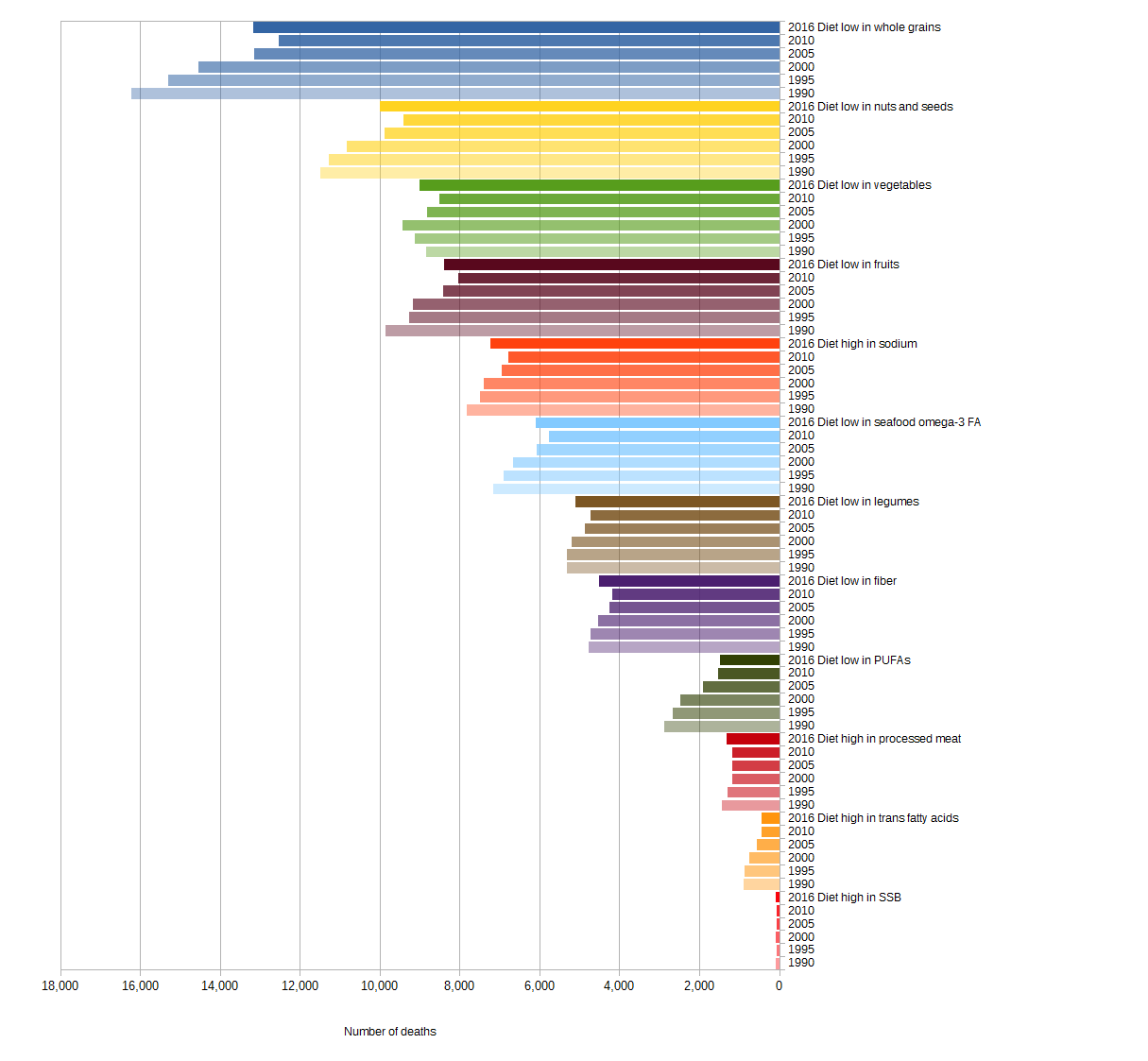

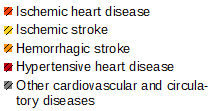
France**


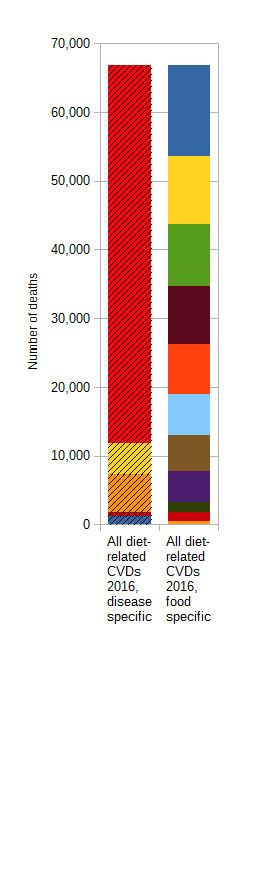


Figure 33 Diet-related CVD deaths from 1990 to 2016 in France

a) Male b) Female


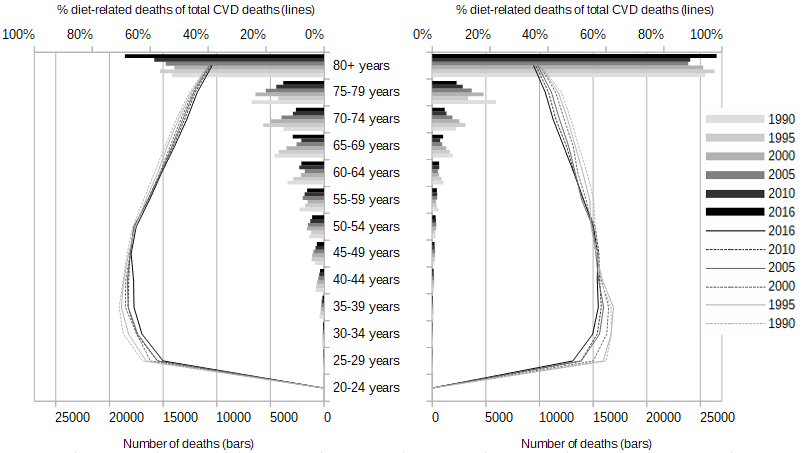


Figure 34 Age- and gender-specific diet-related CVD deaths (bars) and share of diet-related on total CVD deaths (lines) from 1990 to 2016 in France

**
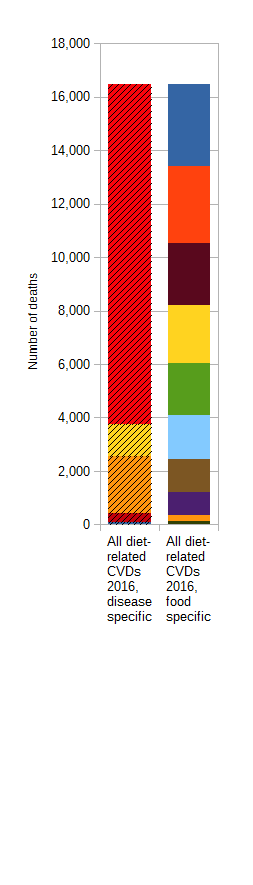

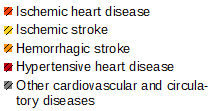

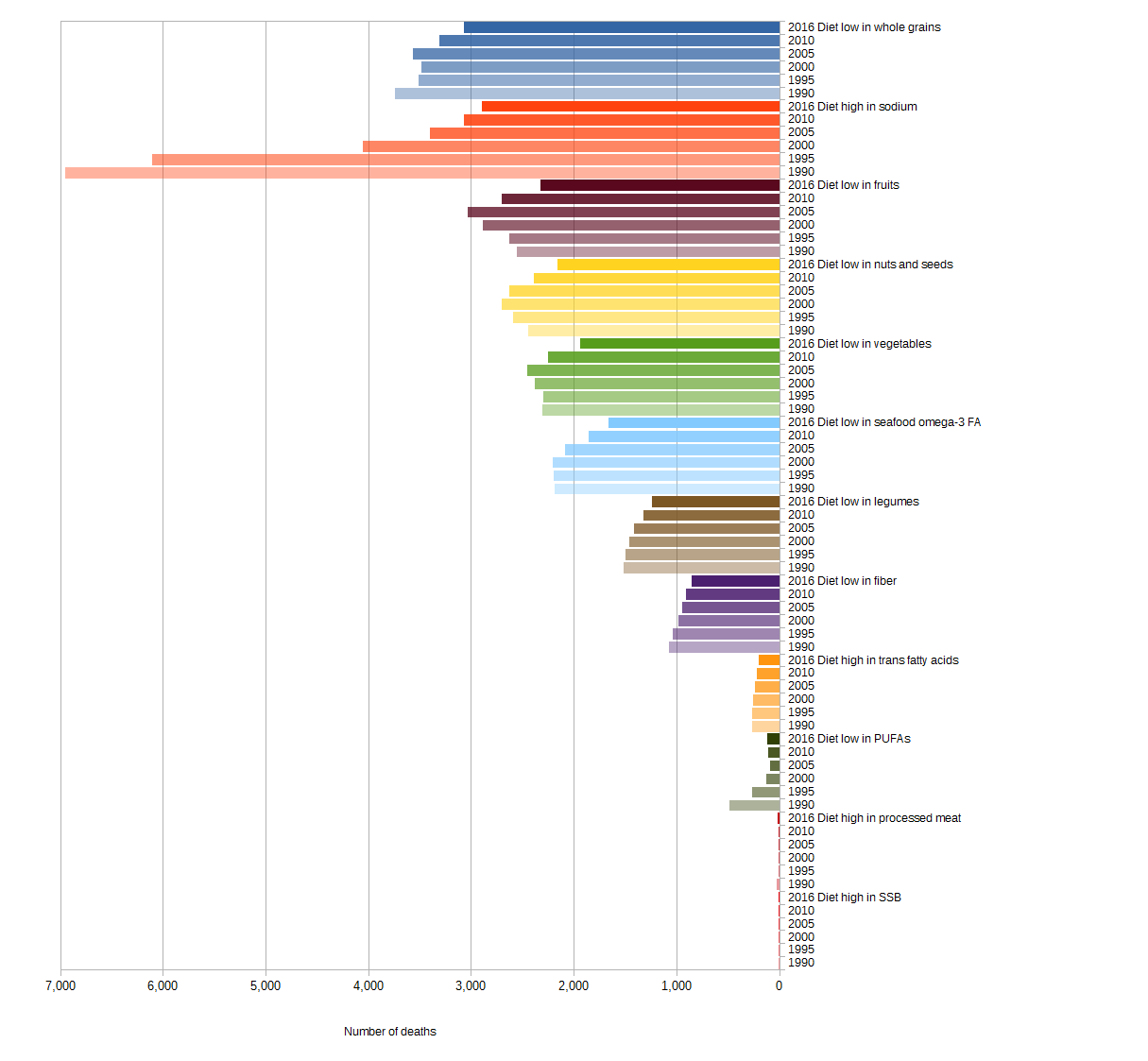
Georgia**

Figure 35 Diet-related CVD deaths from 1990 to 2016 in Georgia

a) Male b) Female


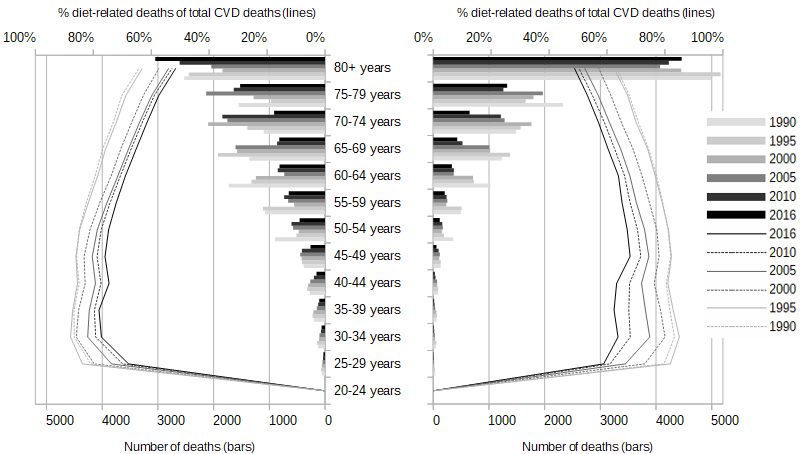


Figure 36 Age- and gender-specific diet-related CVD deaths (bars) and share of diet-related on total CVD deaths (lines) from 1990 to 2016 in Georgia

**
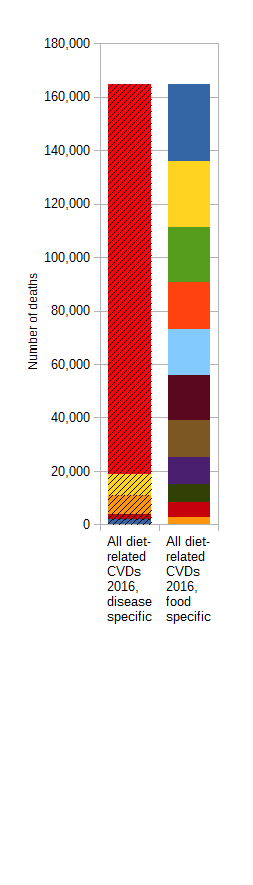

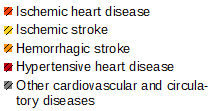

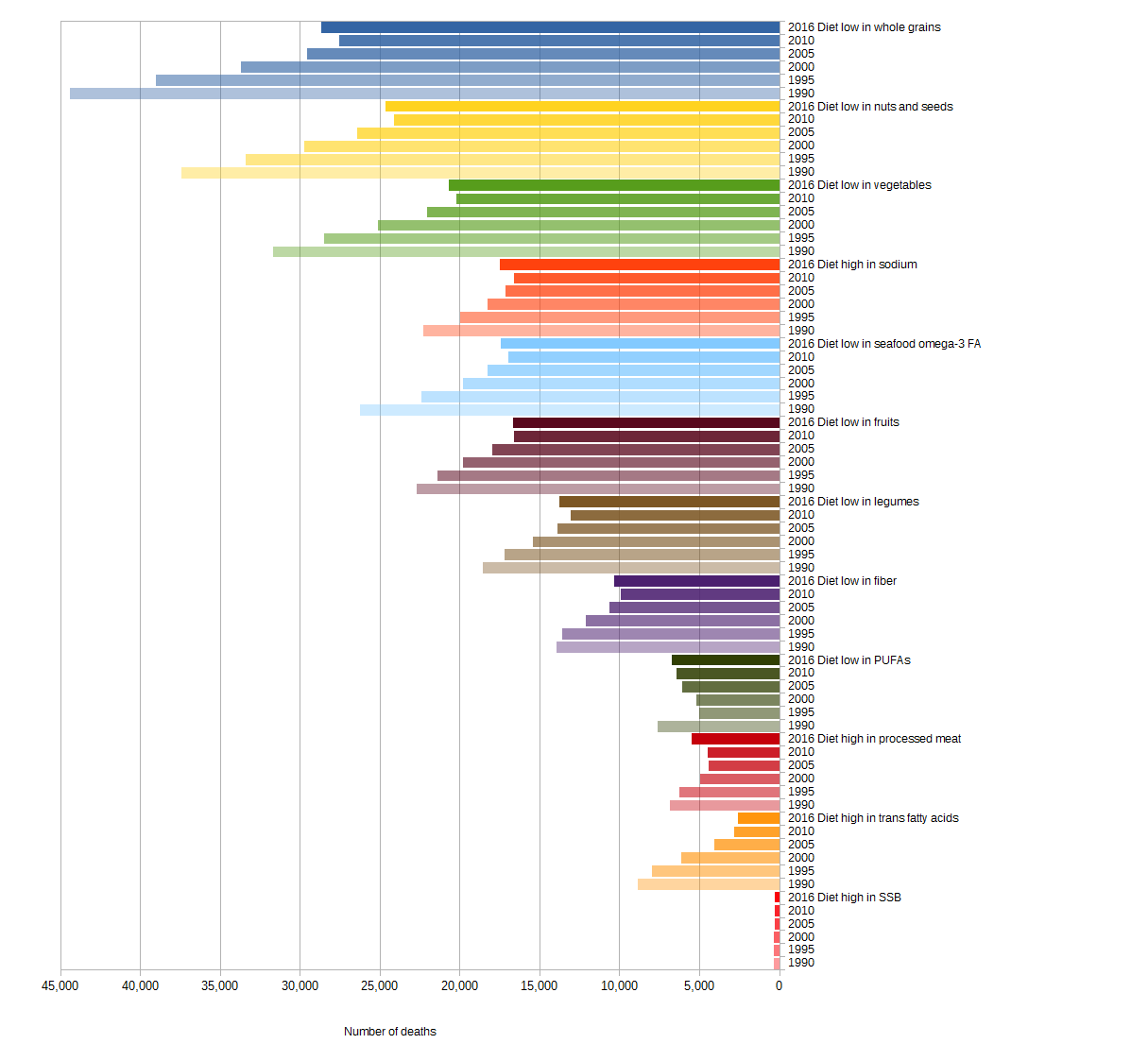
Germany**

Figure 37 Diet-related CVD deaths from 1990 to 2016 in Germany

a) Male b) Female


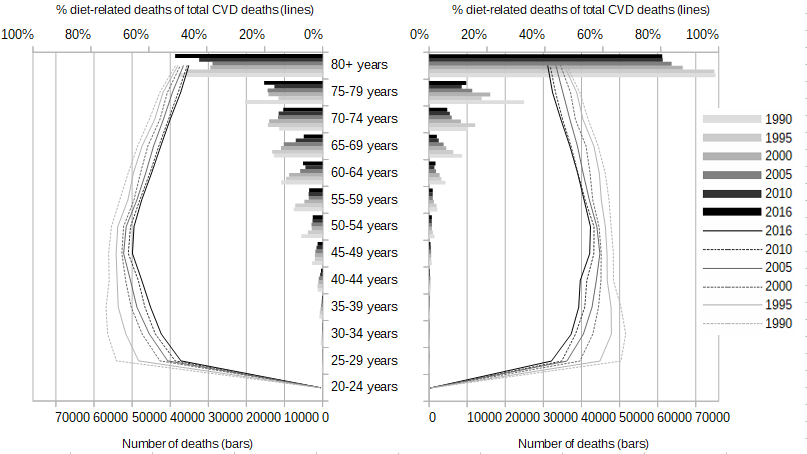


Figure 38 Age- and gender-specific diet-related CVD deaths (bars) and share of diet-related on total CVD deaths (lines) from 1990 to 2016 in Germany

**
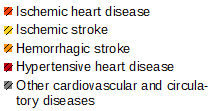

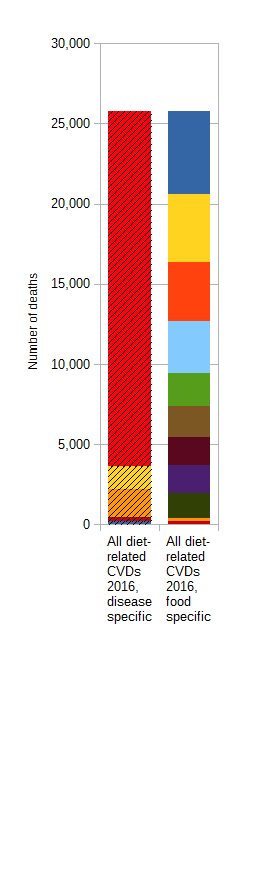

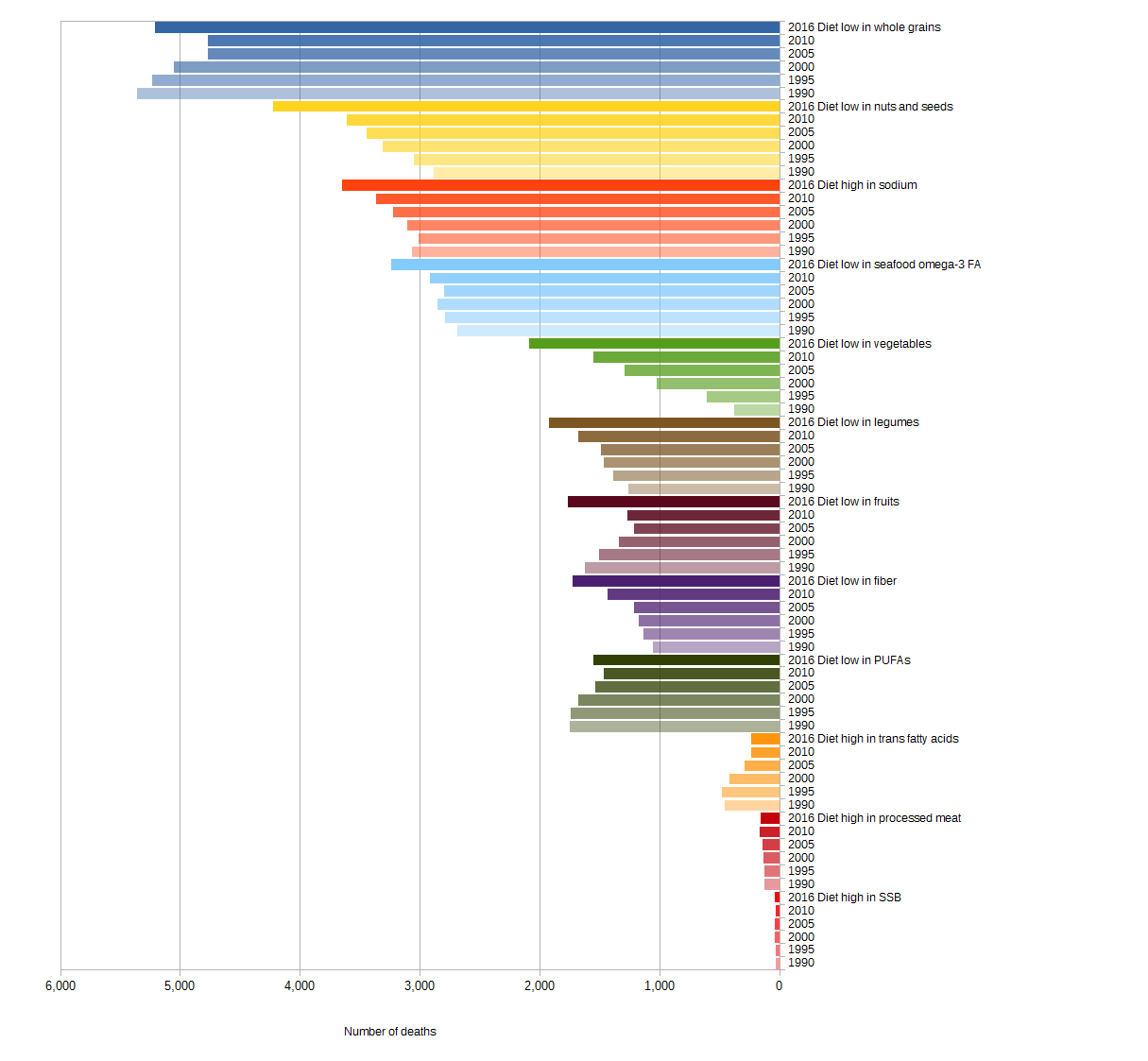
Greece**

Figure 39 Diet-related CVD deaths from 1990 to 2016 in Greece

a) Male b) Female


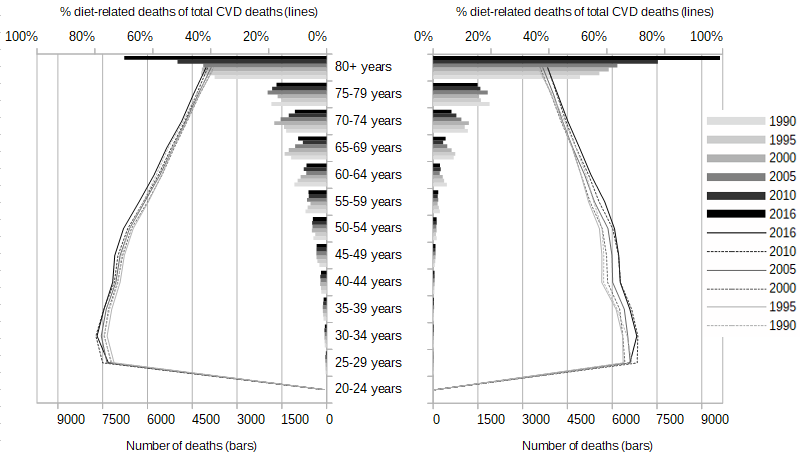


Figure 40 Age- and gender-specific diet-related CVD deaths (bars) and share of diet-related on total CVD deaths (lines) from 1990 to 2016 in Greece

**
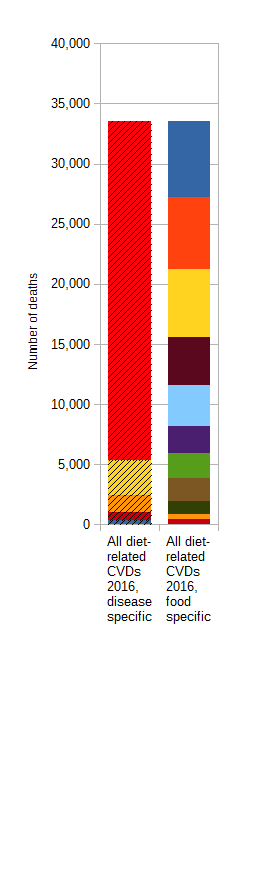

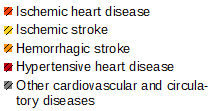

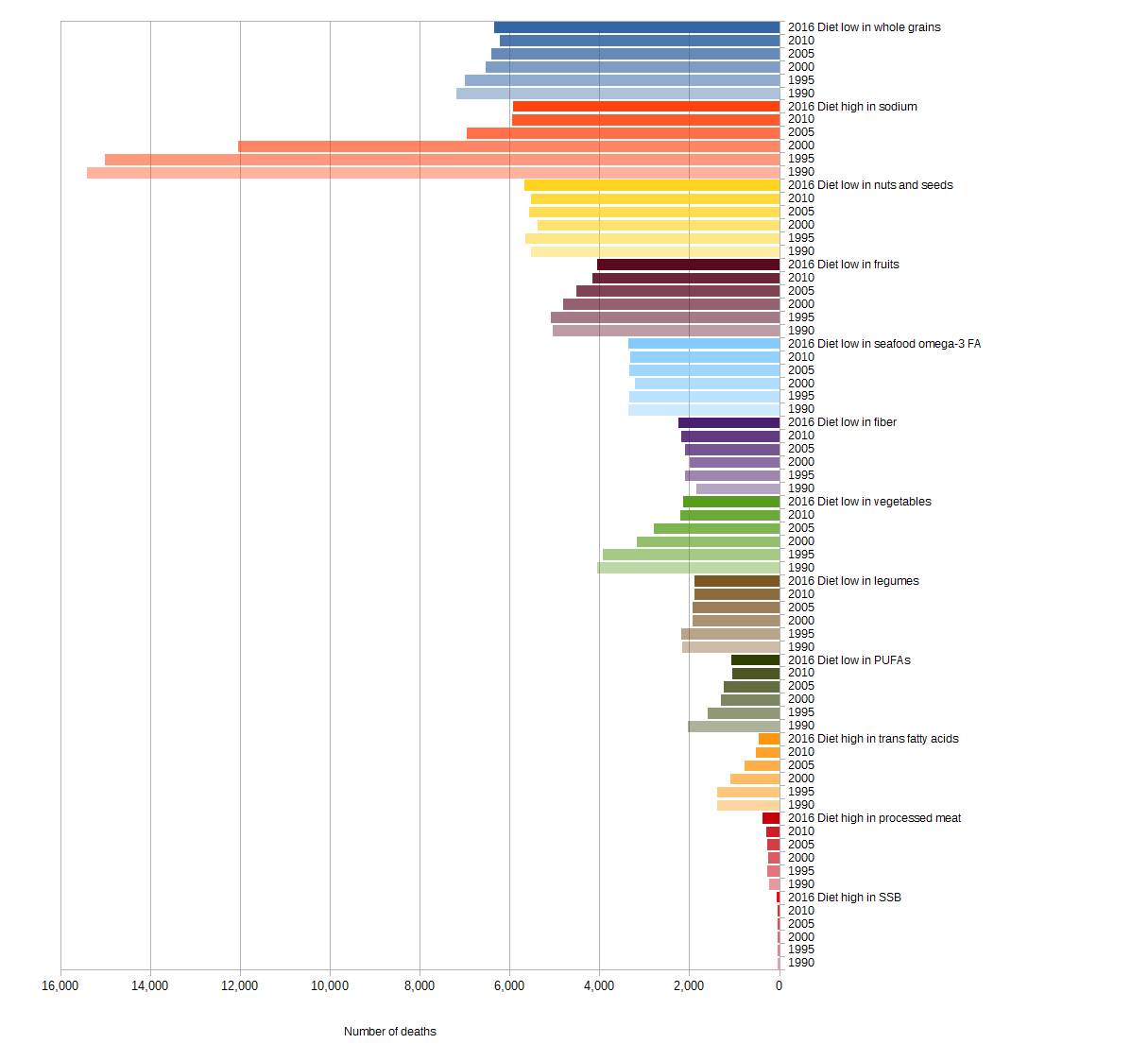
Hungary**

Figure 41 Diet-related CVD deaths from 1990 to 2016 in Hungary

a) Male b) Female


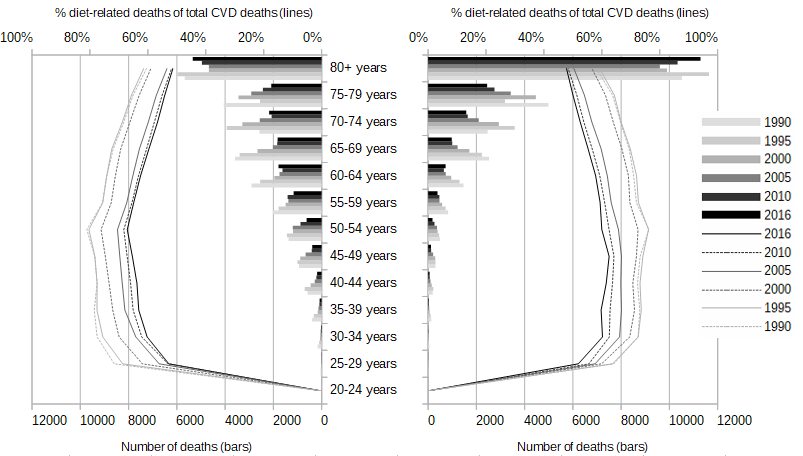


Figure 42 Age- and gender-specific diet-related CVD deaths (bars) and share of diet-related on total CVD deaths (lines) from 1990 to 2016 in Hungary

**
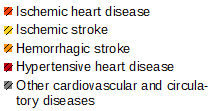

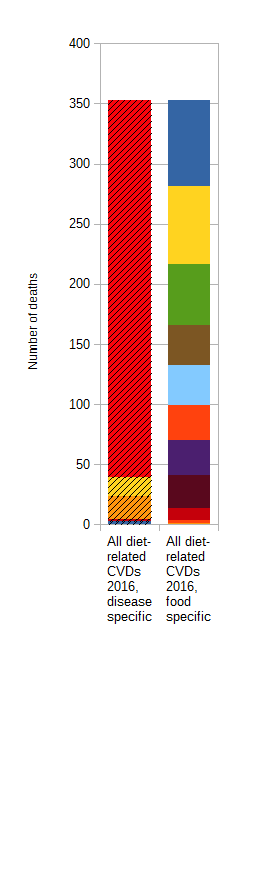

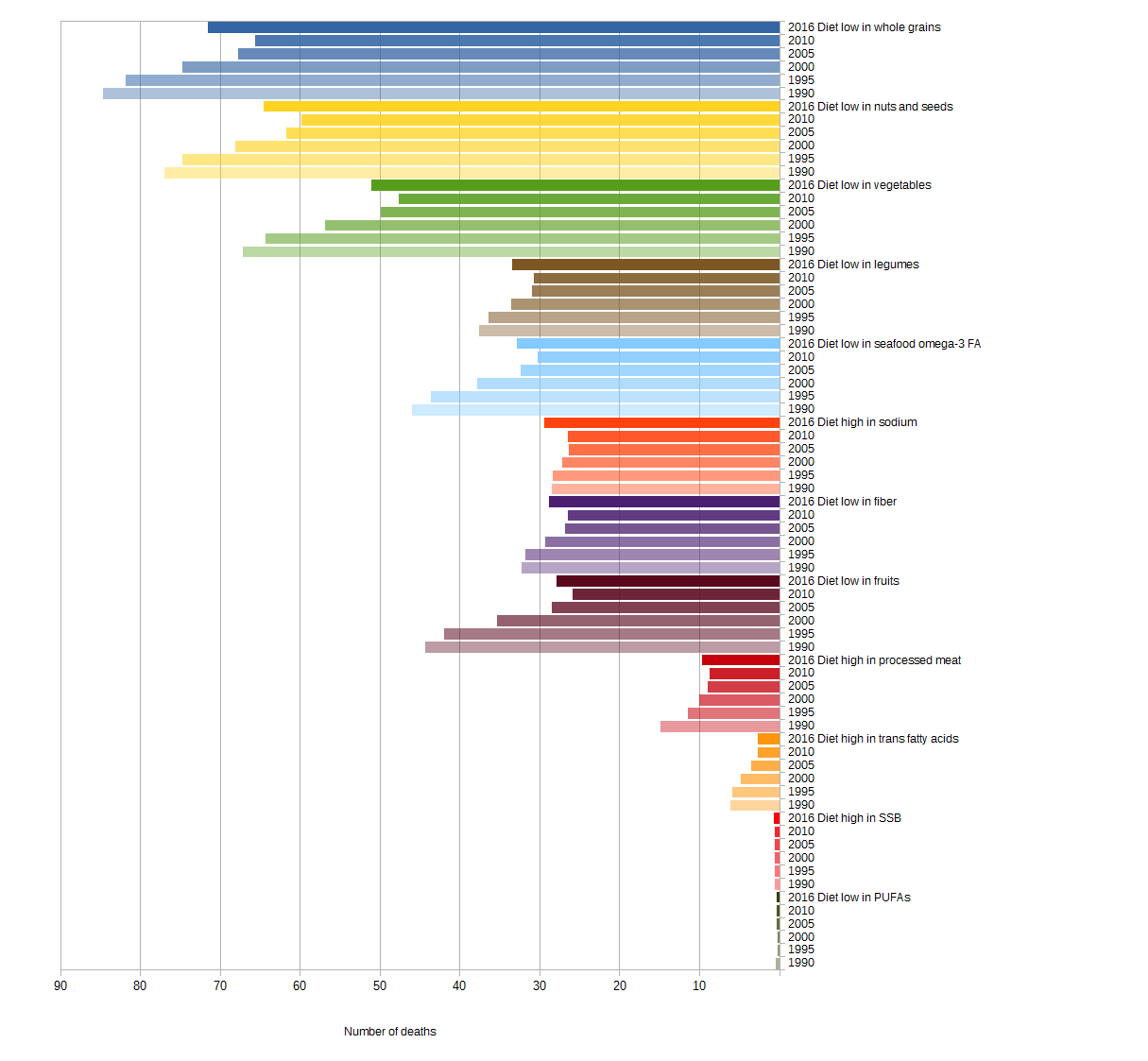
Iceland**

Figure 43 Diet-related CVD deaths from 1990 to 2016 in Iceland

a) Male b) Female


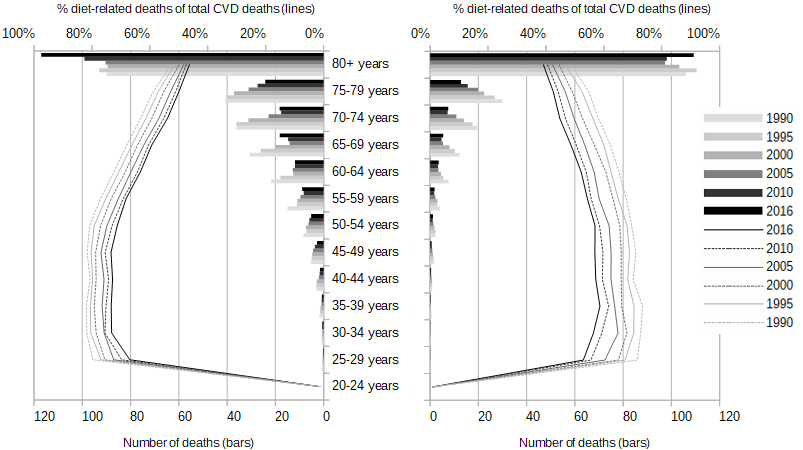


Figure 44 Age- and gender-specific diet-related CVD deaths (bars) and share of diet-related on total CVD deaths (lines) from 1990 to 2016 in Iceland

**
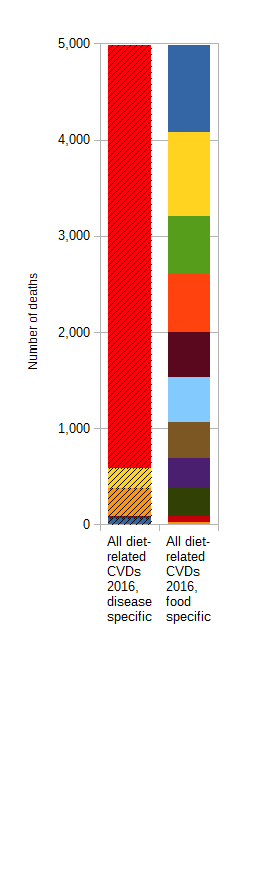

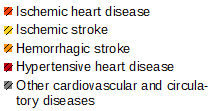

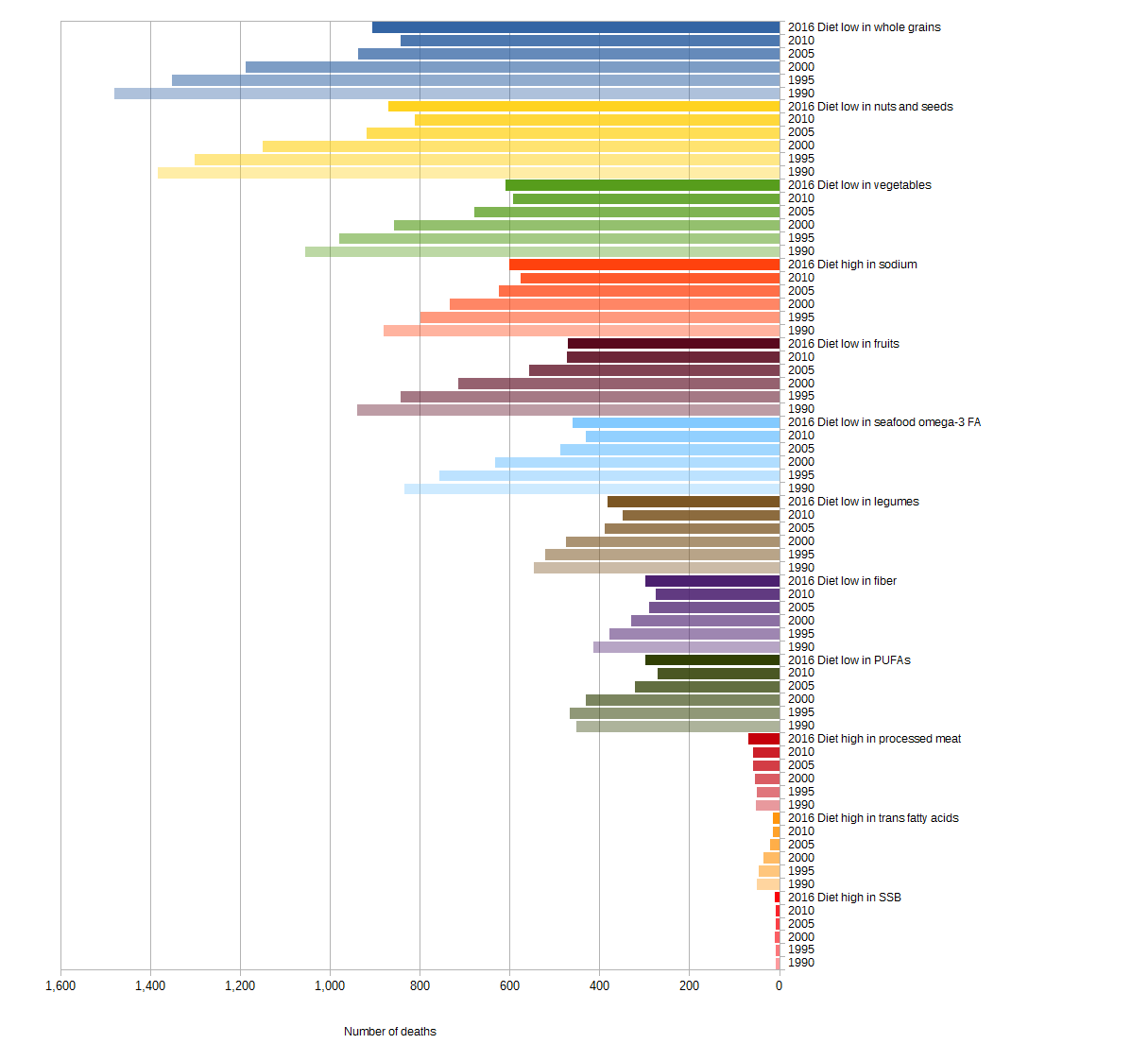
Ireland**

Figure 45 Diet-related CVD deaths from 1990 to 2016 in Ireland

a) Male b) Female


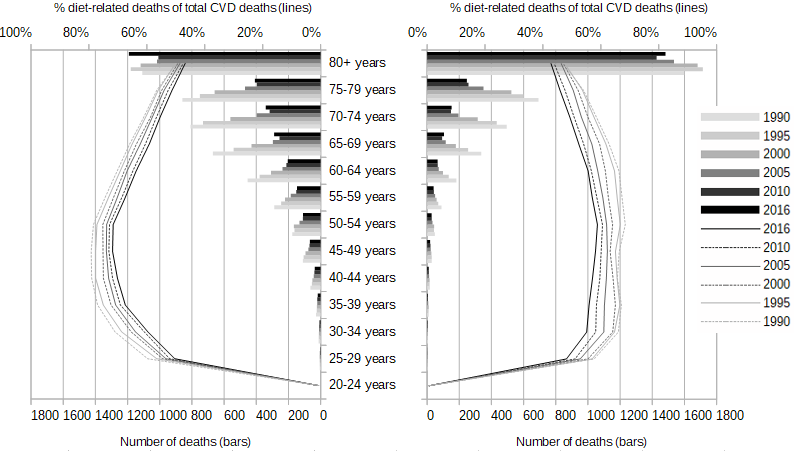


Figure 46 Age- and gender-specific diet-related CVD deaths (bars) and share of diet-related on total CVD deaths (lines) from 1990 to 2016 in Ireland

**
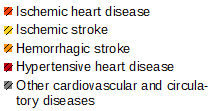

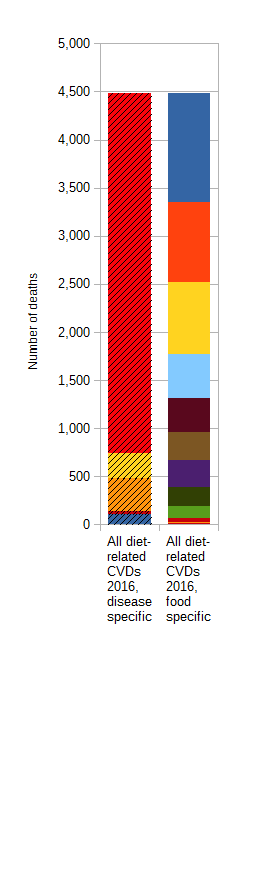

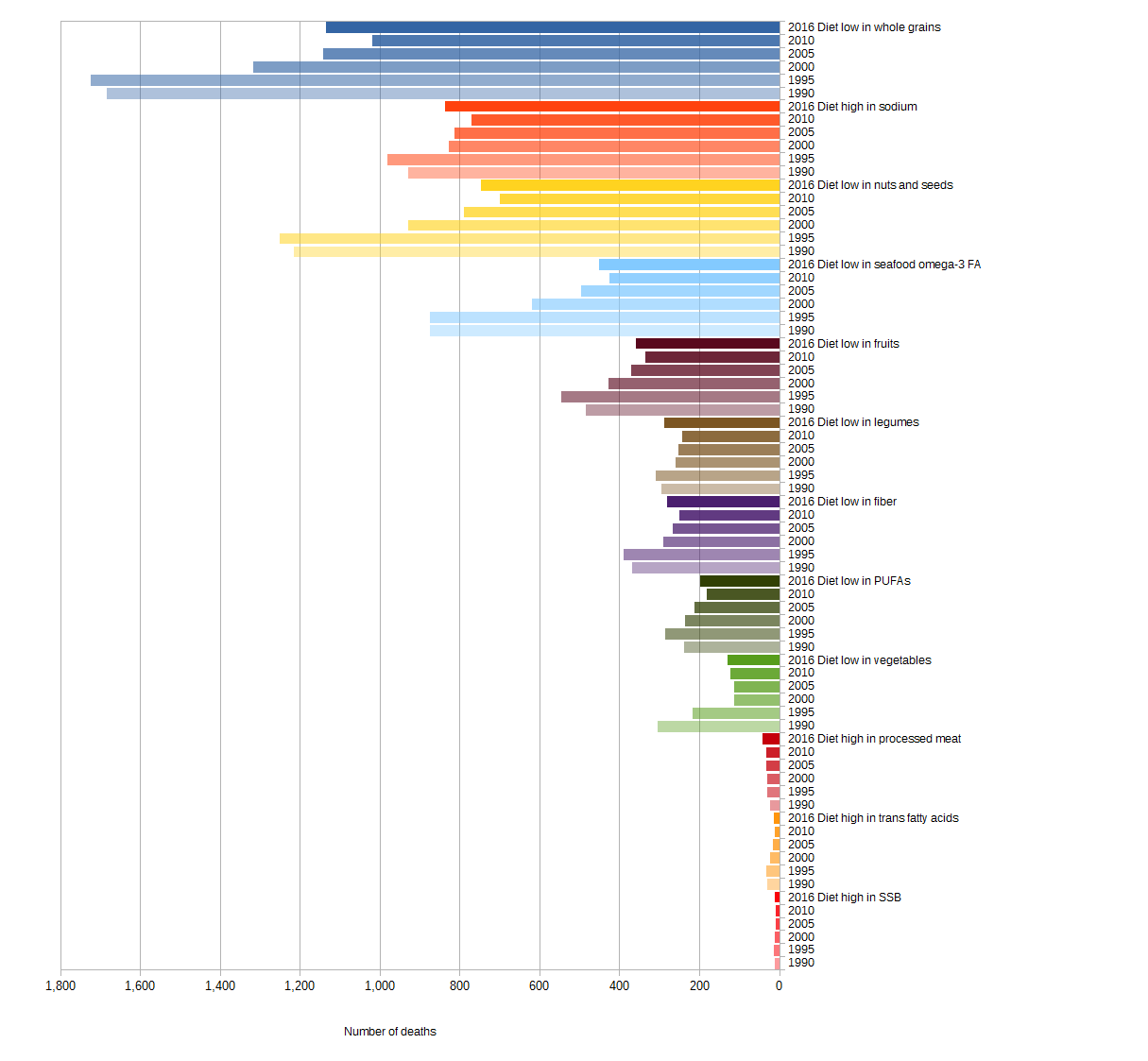
Israel**

Figure 47 Diet-related CVD deaths from 1990 to 2016 in Israel

a) Male b) Female


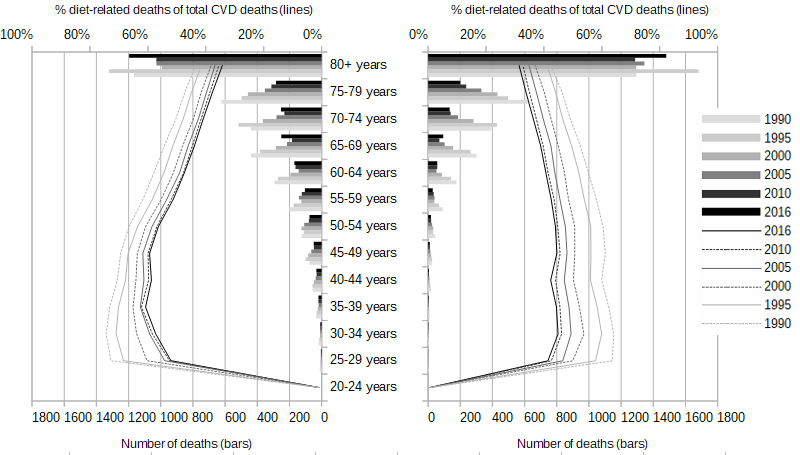


Figure 48 Age- and gender-specific diet-related CVD deaths (bars) and share of diet-related on total CVD deaths (lines) from 1990 to 2016 in Israel

**
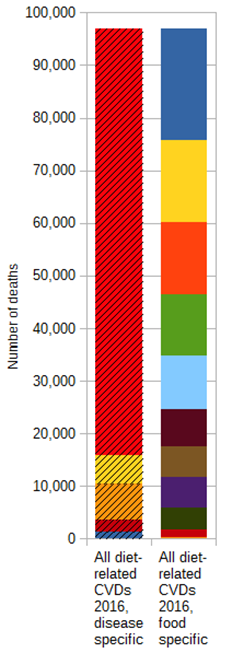

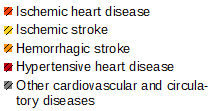

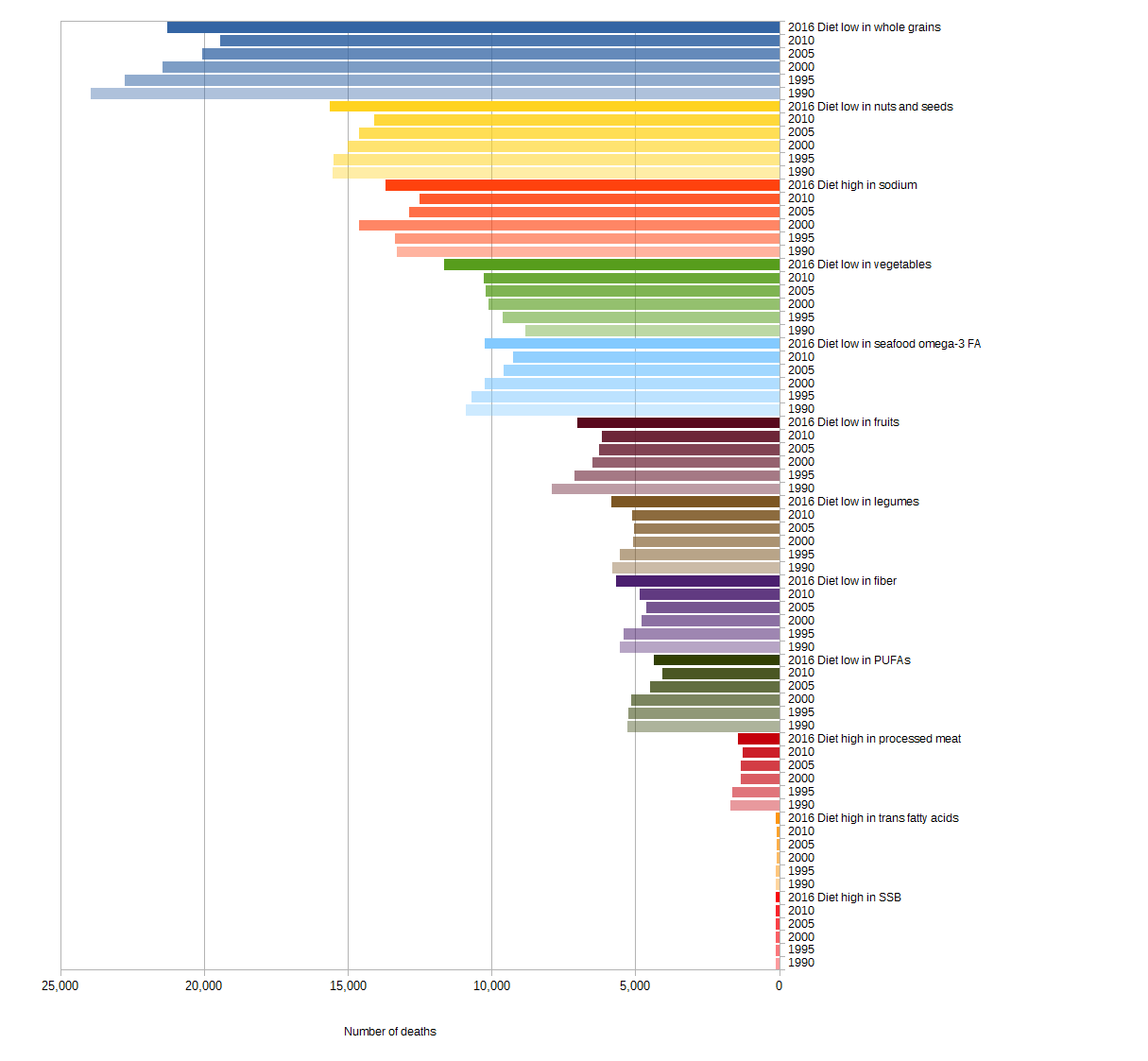
Italy**

Figure 49 Diet-related CVD deaths from 1990 to 2016 in Italy

a) Male b) Female

Figure 50 Age- and gender-specific diet-related CVD deaths (bars) and share of diet-related on total CVD deaths (lines) from 1990 to 2016 in Italy

**Kazakhstan**

Figure 51 Diet-related CVD deaths from 1990 to 2016 in Kazakhstan

a) Male b) Female

Figure 52 Age- and gender-specific diet-related CVD deaths (bars) and share of diet-related on total CVD deaths (lines) from 1990 to 2016 in Kazakhstan

**Kyrgyzstan**

Figure 53 Diet-related CVD deaths from 1990 to 2016 in Kyrgyzstan

a) Male b) Female

Figure 54 Age- and gender-specific diet-related CVD deaths (bars) and share of diet-related on total CVD deaths (lines) from 1990 to 2016 in Kyrgyzstan

**Latvia**

Figure 55 Diet-related CVD deaths from 1990 to 2016 in Latvia

a) Male b) Female

Figure 56 Age- and gender-specific diet-related CVD deaths (bars) and share of diet-related on total CVD deaths (lines) from 1990 to 2016 in Latvia

**Lithuania**

Figure 57 Diet-related CVD deaths from 1990 to 2016 in Lithuania

a) Male b) Female

Figure 58 Age- and gender-specific diet-related CVD deaths (bars) and share of diet-related on total CVD deaths (lines) from 1990 to 2016 in Lithuania

**Luxembourg**

Figure 59 Diet-related CVD deaths from 1990 to 2016 in Luxembourg

a) Male b) Female

Figure 60 Age- and gender-specific diet-related CVD deaths (bars) and share of diet-related on total CVD deaths (lines) from 1990 to 2016 in Luxembourg

**Macedonia**

Figure 61 Diet-related CVD deaths from 1990 to 2016 in Macedonia

a) Male b) Female

Figure 62 Age- and gender-specific diet-related CVD deaths (bars) and share of diet-related on total CVD deaths (lines) from 1990 to 2016 in Macedonia

**Malta**

Figure 63 Diet-related CVD deaths from 1990 to 2016 in Malta

a) Male b) Female

Figure 64 Age- and gender-specific diet-related CVD deaths (bars) and share of diet-related on total CVD deaths (lines) from 1990 to 2016 in Malta

**Moldova**

Figure 65 Diet-related CVD deaths from 1990 to 2016 in Moldova

a) Male b) Female

Figure 66 Age- and gender-specific diet-related CVD deaths (bars) and share of diet-related on total CVD deaths (lines) from 1990 to 2016 in Moldova

**Montenegro**

Figure 67 Diet-related CVD deaths from 1990 to 2016 in Montenegro

a) Male b) Female

Figure 68 Age- and gender-specific diet-related CVD deaths (bars) and share of diet-related on total CVD deaths (lines) from 1990 to 2016 in Montenegro

**Netherlands**

Figure 69 Diet-related CVD deaths from 1990 to 2016 in the Netherlands

a) Male b) Female

Figure 70 Age- and gender-specific diet-related CVD deaths (bars) and share of diet-related on total CVD deaths (lines) from 1990 to 2016 in the Netherlands

**Norway**

Figure 71 Diet-related CVD deaths from 1990 to 2016 in Norway

a) Male b) Female

Figure 72 Age- and gender-specific diet-related CVD deaths (bars) and share of diet-related on total CVD deaths (lines) from 1990 to 2016 in Norway

**Poland**

Figure 73 Diet-related CVD deaths from 1990 to 2016 in Poland

a) Male b) Female

Figure 74 Age- and gender-specific diet-related CVD deaths (bars) and share of diet-related on total CVD deaths (lines) from 1990 to 2016 in Poland

**Portugal**

Figure 75 Diet-related CVD deaths from 1990 to 2016 in Portugal

a) Male b) Female

Figure 76 Age- and gender-specific diet-related CVD deaths (bars) and share of diet-related on total CVD deaths (lines) from 1990 to 2016 in Portugal

**Romania**

Figure 77 Diet-related CVD deaths from 1990 to 2016 in Romania

a) Male b) Female

Figure 78 Age- and gender-specific diet-related CVD deaths (bars) and share of diet-related on total CVD deaths (lines) from 1990 to 2016 in Romania

**Russia**

Figure 79 Diet-related CVD deaths from 1990 to 2016 in Russia

a) Male b) Female

Figure 80 Age- and gender-specific diet-related CVD deaths (bars) and share of diet-related on total CVD deaths (lines) from 1990 to 2016 in Russia

**Serbia**

Figure 81 Diet-related CVD deaths from 1990 to 2016 in Serbia

a) Male b) Female

Figure 82 Age- and gender-specific diet-related CVD deaths (bars) and share of diet-related on total CVD deaths (lines) from 1990 to 2016 in Serbia

**Slovakia**

Figure 83 Diet-related CVD deaths from 1990 to 2016 in Slovakia

a) Male b) Female

Figure 84 Age- and gender-specific diet-related CVD deaths (bars) and share of diet-related on total CVD deaths (lines) from 1990 to 2016 in Slovakia

**Slovenia**

Figure 85 Diet-related CVD deaths from 1990 to 2016 in Slovenia

a) Male b) Female

Figure 86 Age- and gender-specific diet-related CVD deaths (bars) and share of diet-related on total CVD deaths (lines) from 1990 to 2016 in Slovenia

**Spain**

Figure 87 Diet-related CVD deaths from 1990 to 2016 in Spain

a) Male b) Female

Figure 88 Age- and gender-specific diet-related CVD deaths (bars) and share of diet-related on total CVD deaths (lines) from 1990 to 2016 in Spain

**Sweden**

Figure 89 Diet-related CVD deaths from 1990 to 2016 in Sweden

a) Male b) Female

Figure 90 Age- and gender-specific diet-related CVD deaths (bars) and share of diet-related on total CVD deaths (lines) from 1990 to 2016 in Sweden

**Switzerland**

Figure 91 Diet-related CVD deaths from 1990 to 2016 in Switzerland

a) Male b) Female

Figure 92 Age- and gender-specific diet-related CVD deaths (bars) and share of diet-related on total CVD deaths (lines) from 1990 to 2016 in Switzerland

**Tajikistan**

Figure 93 Diet-related CVD deaths from 1990 to 2016 in Tajikistan

a) Male b) Female

Figure 94 Age- and gender-specific diet-related CVD deaths (bars) and share of diet-related on total CVD deaths (lines) from 1990 to 2016 in Tajikistan

**Turkey**

Figure 95 Diet-related CVD deaths from 1990 to 2016 in Turkey

a) Male b) Female

Figure 96 Age- and gender-specific diet-related CVD deaths (bars) and share of diet-related on total CVD deaths (lines) from 1990 to 2016 in Turkey

**Turkmenistan**

Figure 97 Diet-related CVD deaths from 1990 to 2016 in Turkmenistan

a) Male b) Female

Figure 98 Age- and gender-specific diet-related CVD deaths (bars) and share of diet-related on total CVD deaths (lines) from 1990 to 2016 in Turkmenistan

**Ukraine**

Figure 99 Diet-related CVD deaths from 1990 to 2016 in Ukraine

a) Male b) Female

Figure 100 Age- and gender-specific diet-related CVD deaths (bars) and share of diet-related on total CVD deaths (lines) from 1990 to 2016 in Ukraine

**United Kingdom**

Figure 101 Diet-related CVD deaths from 1990 to 2016 in the United Kingdom

a) Male b) Female

Figure 102 Age- and gender-specific diet-related CVD deaths (bars) and share of diet-related on total CVD deaths (lines) from 1990 to 2016 in the United Kingdom

**Uzbekistan**

Figure 103 Diet-related CVD deaths from 1990 to 2016 in Uzbekistan

a) Male b) Female

Figure 104 Age- and gender-specific diet-related CVD deaths (bars) and share of diet-related on total CVD deaths (lines) from 1990 to 2016 in Uzbekistan

1. Murray CJ, Lopez AD. Global mortality, disability, and the contribution of risk factors: Global Burden of

   Disease Study. Lancet 1997; 349: 1436–42. [↑](#footnote-ref-1)
2. GBD 2016 Risk Factors Collaborators. Global, regional, and national comparative risk assessment of 84 behavioural, environmental and occupational, and metabolic risks or clusters of risks, 1990-2016. The Lancet 2017;390:1345–422. [↑](#footnote-ref-2)
3. Vos, T., Allen, C., Arora, M., Barber, R. M., Bhutta, Z. A., Brown, A., ... & Coggeshall, M. (2016). Global, regional, and national incidence, prevalence, and years lived with disability for 310 diseases and injuries, 1990–2015: a systematic analysis for the Global Burden of Disease Study 2015. The Lancet, 388(10053), 1545-1602. [↑](#footnote-ref-3)
4. Danaei G, Singh GM, Paciorek CJ, et al. (2013). The global cardiovascular risk transition: associations of four metabolic risk factors with national income, urbanization, and Western diet in 1980 and 2008. Circulation; 127: 1493–502, 1502e1-8. [↑](#footnote-ref-4)
5. GBD 2016 Risk Factors Collaborators. Global, regional, and national comparative risk assessment of 84 behavioural, environmental and occupational, and metabolic risks or clusters of risks, 1990-2016. The Lancet 2017;390:1345–422. [↑](#footnote-ref-5)
